# Burden of cancer attributable to occupational asbestos exposure in the Americas, 1990–2023: an analysis using the Global Burden of Disease Study 2023

**DOI:** 10.1016/j.lana.2026.101463

**Published:** 2026-04-02

**Authors:** GBD 2023 Americas Occupational Exposure Collaborators, Flavia Araujo Girardi, Michael Brauer, Lisa M. Force, Simon I. Hay, Sandra Spearman, Deborah Carvalho Malta, Maria Teresa Bustamante-Teixeira, Mario Cirio Nogueira, Maximiliano Ribeiro Guerra, Lisa C. Adams, Kamoru Ademola Adedokun, Oluwatobi E. Adegbile, Ali M. Alfalki, Mustafa Alkhawam, Intima Alrimawi, Demelash Areda, Sina Azadnajafabad, Franca Barbic, Abiye Assefa Berihun, Arushee Bhatnagar, Alejandro Botero Carvajal, Carlos A. Castañeda-Orjuela, Vijay Kumar Chattu, Sunghyun Chung, Xiaochen Dai, Wendel Mombaque dos Santos, Osamudiamen Ebohon, Ibrahim Farahat El Bayoumy, Elochukwu Ezenwankwo, Xiangning Fan, Abdelrahman Gamil Gad, Ali Gholamrezanezhad, Muhammad Hamza Ilyas, Mohamed Jalloh, Armaan Jamal, Nathan T. Jibat, Arun Kamireddy, Ramat T. Kamorudeen, Samuel Berchi Kankam, Ibraheem M. Karaye, Khaled Khatab, Farbod Khosravi, Adnan Kisa, Lokesh Manjani, Tomislav Mestrovic, Ali H. Mokdad, Seyed Mohamad Sadegh Mousavi Kiasary, Christopher J.L. Murray, Mahmoud Nassar, Abigia Ashenafi Negash, Meti T. Negassa, Andrew T. Olagunju, Atakan Orscelik, Parinaz Paranjkhoo, Neel Navinkumar Patel, Shrikant Pawar, Farzad Pourghazi, Jagadeesh Puvvula, Mamunur Rashid, Jefferson Antonio Buendia Rodriguez, Sharmistha Roy, Cameron John Sabet, Allen Seylani, Samendra P. Sherchan, Jasvinder A. Singh, Sebastian Straube, Chen-Yang Su, Jabeen Taiba, Aliscia Vieira, Katrin Burkart

**Keywords:** Global burden of disease, Occupational cancer, Asbestos, Epidemiology, Mesothelioma, Lung cancer, Laryngeal cancer, Ovarian cancer

## Abstract

**Background:**

Asbestos remains a leading occupational carcinogen, particularly in countries where its use persists despite known health risks. This study provides a systematic analysis of the burden of cancer attributable to occupational asbestos exposure in the Americas from 1990 to 2023, using estimates from the Global Burden of Disease (GBD) Study 2023. Age-standardised mortality and disability-adjusted life-years (DALYs) attributable to asbestos were analysed for mesothelioma, lung, laryngeal, and ovarian cancers, stratified by sex and region.

**Methods:**

We conducted a descriptive analysis to assess spatiotemporal trends in the burden of cancer attributable to occupational asbestos in the Americas from 1990 to 2023. We analysed trends in age-standardized mortality and DALY rates using segmented joinpoint regression. Age-period-cohort analyses were performed for age-specific mortality and DALY rates. All analyses were stratified by cancer type, sex and GBD regions, with estimated 95% uncertainty intervals (95% UI).

**Findings:**

In 2023, High-income North America had the highest burden of cancer attributable to occupational asbestos, with 5·1 deaths (95% UI 3·9; 6·4) and 84·9 DALYs (65·6; 108·5) per 100,000 population for both sexes. However, the region also experienced the most pronounced decline, with average annual reductions of 2·0% (−2·0; −1·9) in mortality and 2·5% (−2·5; −2·4) in DALYs. Southern Latin America had the second highest rates for cancer attributable to occupational asbestos, with 2·7 deaths (2·1; 3·5) and 53·1 DALYs (40·4; 69·3) for both sexes in 2023, and showed the strongest increase in women with 2·3% (2·2; 2·4) both in mortality and DALYs annually. Age-period-cohort modelling revealed marked increases in burden of cancer attributable to occupational asbestos among women, with mortality and DALY rate ratios (RR) for lung cancer rising to 1·31 (1·20; 1·44) in Tropical and Southern Latin America, and RR for mesothelioma rising to 1·22 (1·06; 1·40) in Southern Latin America.

**Interpretation:**

Our study revealed inequalities in the burden of cancer attributable to occupational asbestos exposure among regions in the Americas, as well as remarkable sex disparities. Although rates were declining in North America, there is growing concern over rising rates of lung cancer and mesothelioma among women in Tropical and Southern Latin America regions, especially in Argentina and Brazil. These disparities likely reflect differences in environmental and industrial regulatory practices, as well as gendered occupational exposure patterns. Also, upward trends in female lung cancer rates may reflect increased smoking among women, while mesothelioma is much more specific to asbestos exposure. Despite regulatory advances, legacy exposures and ongoing asbestos use persist in parts of Latin America, reinforcing the need for stricter occupational health policies and asbestos bans. The findings underscore the shifting epidemiology of asbestos-related cancers and call for targeted prevention efforts, improved surveillance, and gender-responsive occupational protections.

**Funding:**

This study was partially funded by the 10.13039/100000865Bill & Melinda Gates Foundation and “*Coordenação de Aperfeiçoamento de Pessoal de Nível Superior–Brasil* (CAPES)”.

**Research in context**

**Evidence before this study**

To assess the landscape on cancer attributable to occupational asbestos exposure we searched PubMed and Scopus, focusing on publications evaluating epidemiological aspects worldwide and in the Americas. We also searched for asbestos-related regulations and legislation on specific government websites. The bibliographic research was carried out between August and September 2024, using the search terms (“occupational cancer” AND “asbestos”) OR (“occupational asbestos” AND “epidemiology”) and we included papers published in English, Portuguese and Spanish. We excluded studies related to other carcinogens, cancer diagnostic methods or treatment. Many studies described evidences of asbestos as a cause of laryngeal, lung, ovarian cancer and mesothelioma. However, previous studies have focused on the global burden or individual countries, or single cancer types, limiting cross-regional comparisons. Only a few studies addressed mortality and disability-adjusted life years (DALY) rates using an age-period-cohort analysis. Overall, previous studies highlighted higher burden of occupational cancer in high-income countries. The Americas, spanning a wide range of income groups, include some of the largest historical producers of asbestos, and few studies compared the burden of occupational cancer across their regions or discussed differences in asbestos regulations.

**Added value of this study**

Our study added substantial value to existing evidence by providing a comprehensive and internally consistent approach to the burden of cancer attributable to occupational asbestos exposure (specifically lung, laryngeal, and ovarian cancers and mesothelioma) in the Americas. The Global Burden of Disease Study (GBD) is a systematic and inherently consistent study that synthesizes all available data allowing for temporo-spatial comparison. We evaluated mortality and DALY rates and described cancer trends over time to present patterns of burden across regions within the Americas for men and women. To more effectively assess the separate effects of age, period, and birth cohort on estimated rates, we conducted age–period–cohort analyses. Most studies that analyze disease burden longitudinally do not separately consider the effects of the three temporal dimensions. The separate assessment allows us to identify the most affected groups and better target possible interventions. The results offered comprehensive insights into the asbestos-associated occupational cancer burden across different age groups and highlighted notable sex disparities.

**Implications of all the available evidence**

This study serves as a step towards recognizing the broader picture of the impact of asbestos on the health of workers in the Americas, which still maintains high levels of asbestos consumption. The High-income North America region had the highest rates of occupational cancer, but showed decreasing trends over the period and across birth cohorts. In contrast, increasing rates were observed in Tropical and Southern Latin America, especially for lung cancer and mesothelioma in women. Future research should also include analyses of non-occupational asbestos exposure, as this carcinogen poses a significant environmental threat globally.

## Introduction

Asbestos is the name given to a group of mineral fibers that occur naturally in the environment and it is widely used in industrial applications due to their heat resistance and durability, primarily to produce ceiling and floor tiles, brakes, dry wall, roofs, cement and protective clothing.^1^ Asbestos is a cause of non-malignant diseases such as asbestosis and pleural abnormalities,^1^ and was recognized as a carcinogen by the International Agency for Research on Cancer (IARC) in 1973.^2^ All forms of asbestos mineral fibers are carcinogenic, but the amphibole subtype is considered more harmful to human health, as they have longer fibers than the chrysotile subtype and remains retained in the lungs for longer.^1^

Asbestos is considered a risk factor for mesothelioma and laryngeal, lung and ovarian cancers.^3^ Lung cancer is the highest contributor to asbestos-related neoplasms burden,^4^ but mesothelioma is the malignant neoplasm with the best-established causal relationship with asbestos, since the mesothelial cells of the pleura and peritoneum are highly susceptible to its fibers.^1^ Associations with laryngeal and ovarian cancer were also recognized by IARC.^2^ The distribution of asbestos fibers throughout the body after inhalation and how they reach the ovaries remains unclear. The fibers could migrate through the diaphragm or access the lymphatic system and reach the peritoneal cavity. The accumulation of fibers in the ovaries could lead to persistent local inflammation and genetic alterations.^5^

Globally, asbestos remained the leading risk factor for occupational cancer in 2023, with approximately 230,698 attributable deaths and 4·1 million disability-adjusted life-years (DALYs).^6^ High-income countries displayed high burden of cancer attributable to occupational asbestos, but trends in mortality and DALY rates declined between 1990 and 2019.^4^ In contrast, low to middle-income countries showed increasing rates over the same time period.^4^ In the Americas, some countries in Andean, Tropical and Southern Latin America exhibited these upward trends.^4^

Worldwide, asbestos exposure decreased only 0·4% between 1990 and 2023^7^ and there is still considerable use in Asia, Africa and Latin America.^8^ In 2023, Russia led asbestos mine production, followed by Kazakhstan, China and Brazil. While more than 70 countries having banned asbestos today, major producers continue to mine and export asbestos to countries where effective and safe substitutes are still not available.^9^ Within the Americas, phase-outs have been implemented only in the United States of America (US), Canada, Greenland, Honduras, Colombia, Brazil, Argentina, Chile and Uruguay, resulting in continued exposure of many workers in extraction and manufacturing activities.^10^ Furthermore, as the workforce is commonly marked by sex disparities, the burden of cancer attributable to occupational asbestos may impact men and women differently.^8^ Men make up the majority of workers in the mining, construction, and chemical industries. However, there is a misconception that women's work is generally safer, due to the relatively small number of women employed in hazardous activities. As the presence of women in the workforce is growing, there is a need to better assess male and female exposures separately.^11^

Because there is a lack of large-scale, up-to-date studies addressing the burden of cancer attributable to occupational asbestos among workers in the Americas, we conducted this study to better determine estimates and their variations across age-groups and sexes. This manuscript was produced as part of the Global Burden of Disease (GBD) Collaborator Network and in accordance with the GBD Protocol.^12^

## Methods

### Overview

This is a descriptive analysis conducted to assess the burden of cancer attributable to occupational asbestos exposure and its distribution characteristics in the Americas, using data from GBD 2023. GBD 2023 complies with the Guidelines for Accurate and Transparent Health Estimates Reporting (GATHER)^13^ and Preferred Reporting Items for Systematic Reviews and Meta-Analyses (PRISMA) guidelines.^14^ Analyses were completed using Joinpoint Regression Program version 5·3·0^15^ for segmented regression analyses and R Studio version 4·2·2^16^ for age-period-cohort analyses (Epi package version 2·48^17^).

### Cancer types, geographical units, age groups, and time periods

GBD 2023 linked occupational asbestos exposure to mesothelioma and laryngeal, lung (trachea, bronchi and lungs) and ovarian cancers. Risk-outcome pairs were determined according to the World Cancer Research Fund convincing or probable grades of evidence,^18^ based on biologically plausible associations between exposure and disease established from multiple epidemiological studies.^7^

Countries and territories were grouped into 21 regions, based on geographic proximity and epidemiological similarity.^7^ For the present study, we selected six regions that make up the Americas: High-income North America; Central Latin America; Caribbean; Andean Latin America; Tropical Latin America; and Southern Latin America (see the regions and respective countries in Figure S1). We did not include country-level estimates because some countries presented low rates of asbestos-related cancers, thus making age-period-cohort analyses impossible. Furthermore, grouping by region helped to provide a broader perspective. However, we included country-level results in the Supplementary Material and highlighted selected estimates to illustrate the range of burden as well as sharp differences between countries and the relationship between mortality and DALY rates and the socio-demographic index (SDI).

We considered age-standardized mortality and DALY rates for cancers attributable to occupational asbestos exposure from 1990 to 2023, calculated with 95% uncertainty intervals (95% UI) and presented per 100,000 inhabitants. The GBD calculated age-standardized rates using the GBD world population age standard. A standard population age structure was generated by the non-weighted mean of the 2010 to 2035 age-specific proportional distributions for national locations reported by the United Nations World Population Prospects 2012 revision. From GBD 2017 onwards, the world population age standard was updated using the non-weighted mean of the GBD year's age-specific proportional distributions for national locations with populations greater than five million.^19^

Specifically for the age-period-cohort analysis, we included age-specific groups rates for the population aged 50 years and over, to cover the largest number of estimates and avoid age groups with very few estimates. We used numbers of deaths and DALYs and GBD population data to calculate age group-specific rates from 1994 to 2023.

All metrics were stratified by each cancer, sex and region. Due to the low numbers and rates, we excluded estimates of laryngeal cancer in women from cancer-specific regression trend (but kept them in the analyses of all cancers together) and age-period-cohort analyses (Table S1).

### Data sources and processing

Within the GBD framework, asbestos is the only risk factor for mesothelioma. Due to this, GBD uses mesothelioma mortality to directly calculate occupational asbestos exposures. The Asbestos Impact Ratio (AIR) represents the excess deaths due to mesothelioma observed in a population divided by excess deaths due to mesothelioma in a population heavily exposed to asbestos.^7^ The AIR is calculated by the following equation:

$$AIR=\frac{Mort\;c,y,s\;–N\;c,y,s}{Mort*c,y,s\;–N\;c,y,s}$$

where:

*Mort* = Mortality rate due to mesothelioma;

*Mort*∗ = Mortality rate due to mesothelioma in population highly exposed to asbestos;

*N* = Mortality rate due to mesothelioma in population not exposed to asbestos;

c = country; y = year; s = sex.

Mortality rates due to mesothelioma *(Mort)* were estimated using GBD 2023 causes of death results. Mortality rates due to mesothelioma in populations highly exposed to asbestos *(Mort∗)* were calculated based on Goodman et al., which included only occupational settings of exposure.^20^ Conversely, mortality rates due to mesothelioma in populations not exposed to asbestos *(N)* were based on Lin et al., which measured the exposure of general population through asbestos consumption and, therefore, did not consider direct exposure from occupational sources.^21^ Although most exposure is occupational, it is possible that our estimation approach includes some contribution from non-occupational sources.

GBD 2023 estimated occupational asbestos exposures for the population aged 15 years and over. Asbestos exposure prevalence created using the AIR was used to estimate population attributable fractions (PAFs), which were calculated and computed for each risk–outcome pair. PAFs represent the proportion of disease burden in a population that is attributable to a specific risk factor or exposure. The PAF for the asbestos-mesothelioma risk–outcome pair was calculated directly through the mortality rates. PAFs for other cancers attributable to asbestos were calculated using the exposure determined by the AIR and the respective relative risks by the equation PAF = (relative risk-1)/relative risk^7^. Relative risks were obtained from a systematic review of published meta-analyses, last updated in GBD 2016.^22^

Finally, estimates of attributable burden, such as deaths and DALYs associated with the outcome, were calculated for each combination of age-group, sex, location, and year. DALY is a measure that combines the years lost due to premature death (YLL–Years of Life Lost) and the years lived with disability (YLD–Years Lived with Disability). Detailed methods are available in GBD 2023 Supplementary Material.^7^

### Trend analysis

We analysed trends in age-standardized mortality and DALY rates for cancer attributable to occupational asbestos exposure using a segmented joinpoint regression model. This method identifies trend change points over time, testing whether the observed temporal trends are best explained by single or multiple line segments. We used permutation tests to identify up to six joinpoints and the outputs were log-transformed. Autocorrelated error models were specified to account for first-order temporal correlation in the residuals. Homoscedasticity was assessed through graphical inspection of residuals versus fitted values and time; and formally using White's heteroscedasticity test. Models considered heteroscedastic by White's test were re-run in the Joinpoint software, considering the standard errors.^23^ Annual percentage changes (APC) in mortality and DALY rates were calculated for each time segment between two joinpoints, quantifying trends and statistical significance. Average annual percentage change (AAPC) was used as a summary measure of the trend over the study period, computed as a weighted average of the APCs from the joinpoint model.^24^ We used one-year periods from 1990 to 2023 and the Empirical Quantile method to estimate APC and AAPC 95% confidence intervals. Both APC and AAPC significance was determined by p-value <0·05.

### Age-period-cohort analyses

We performed age-period-cohort analyses to identify different contributions of age, period and birth cohort to mortality and DALY rates. Age effects can be defined as changes resulting from the aging process of each individual. Period effects result from external factors that affect all age groups equally at a given point in time. And cohort effects result from the exposure of a group of individuals from the same generation over time, as they age together. Since the relationship between age, period and cohort is linear and correlated, it is impossible to estimate their effects separately, which is known as the identification problem.^25^ In this study, to address the identification problem, we used a parametrization method based on deviations, curves and drifts, proposed by Holford^26^ and adapted by Carstensen,^27^ widely accepted and used. This method limits the analysis of effects to their linear combinations and curvatures. Curvatures are the estimable functions of the parameters and remain constant, regardless of the parameterization used. The linear trend of the effects is divided into two components: the first is the linear effect of age; the other is called drift, which is the linear effect of period and cohort. This method has the disadvantage of requiring constraints. Therefore, the results vary depending on the assumptions used to construct those constraints.

Age-groups were formed from 50 years old, with 5-year increments, and up to the last group aged 80 and over. We used 5-year intervals between 1994 and 2023, as the method requires the same number of years in each interval. Birth cohorts from 1914 to 1969 were established subtracting the age of the period (birth cohort = period—age). Thus, the analysis was composed of seven age-groups, six periods and twelve birth cohorts, stratified by sex, region and each cancer attributable to occupational asbestos exposure. Each group of age, period and cohort were named by the first value in the group: 50–54 years age group was identified as 50 years and so on; 1994–98 period was identified as 1994 and so on; and 1914–18 birth-cohort was identified as 1914 and so on. Medians of male lung cancer data from the High-income North America region were chosen as the reference for models for all cancers and regions in both sexes, for presenting the highest estimated numbers. Therefore, 2004–08 was chosen as a reference period and 1934–38 was the reference birth cohort.

To select the best model, comparisons were performed between two models at a time, where *p* values < 0·05 were considered significant, as follows: (1) age and age-drift models: if significant, it represents a non-linear effect of age; (2) age-drift and age-cohort models: if significant, it represents a non-linear cohort effect; (3) age-cohort and age-period-cohort models: if significant, it represents a non-linear period effect, in the presence of cohort; (4) age-period-cohort and age-period models: if significant, it represents a non-linear cohort effect, in the presence of period; and (5) age-period and age-drift models: if significant, it represents a non-linear period effect^28^ (Table S2). The outputs were presented as estimated age-specific rates of mortality and DALYs and rate ratios (RR), which can be interpreted as relative risks, for each period and cohort according to their respective reference categories. Age effects were expressed by age-specific rates represented as longitudinal curves that showed age-associated natural history of mortality and DALYs. Period effects were expressed as RR of mortality and DALYs to track progress over time. And cohort effects were expressed as RR of mortality and DALYs to track changes in rates for different birth cohorts.^28^ RR significance was determined by p-value <0·05.

### Ethics approval

The Global Burden of Diseases, Injuries, and Risk Factors Study used de-identified data, and the waiver of informed consent was reviewed and approved by the University of Washington Institutional Review Board (study number 9060).

### Role of the funding source

This study was partially funded by the Bill & Melinda Gates Foundation and the “*Coordenação de Aperfeiçoamento de Pessoal de Nível Superior–Brasil* (CAPES)”–Finance Code 001.

The funders of this study had no role in study design, data collection, data analysis, data interpretation, or the writing of the report. The lead and senior authors had full access to the data in the study and final responsibility for the decision to submit for publication.

## Results

Rates for cancer in both sexes attributable to occupational asbestos exposure were higher in High-income North America in 2023, with 5·1 deaths (95% UI 3·9; 6·4) and 84·9 DALYs (65·6; 108·5) per 100,000 population. The US reported the highest estimated numbers, followed by Canada, which had higher rates, with 12·6 (9·2; 16·3) deaths and 204·3 (145·1; 269·3) DALYs for lung cancer in men in 2023. Greenland had the highest rates for all cancers assessed, but numbers were very low (Tables S3 and S4). However, the region showed the most downward trend from 1990 to 2023, with average annual decreases of 2·0% (2·0; −1·9) in mortality and 2·5% (−2·5; −2·4) in DALYs. Southern Latin America had the second highest rates in 2023, with 2·7 deaths (2·1; 3·5) and 53·1 DALYs (40·4; 69·3), followed by Tropical Latin America, with 1·7 deaths (1·4; 2·2) and 32·9 DALYs (26·6; 40·8). Rates in the Caribbean and Central Latin America showed decreasing trends or stability during the period. Higher asbestos-attributable mortality and DALY rates were generally observed in countries with higher SDI (Figures S2, S3 and S4).

**Fig. 1:**
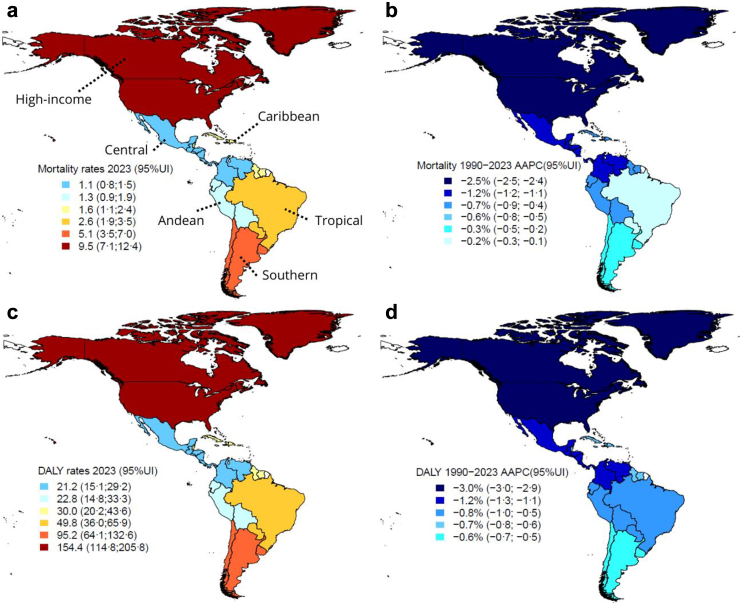
**Age-standardised rates (2023) and average annual percentage change (1990–2023) of (a, b) mortality and (c, d) disability-adjusted life years (DALY) for cancer in men attributable to occupational asbestos exposure by regions in the Americas.** Mortality: age-standardized mortality rates per 100,000; DALY: age-standardized DALY rates per 100,000; AAPC: average annual percentage change; 95% UI: 95% uncertainty interval. High-income: High-income North America; Central: Central Latin America; Andean: Andean Latin America; Tropical: Tropical Latin America; Southern: Southern Latin America. Cancer in men: laryngeal and lung cancers and mesothelioma. Elaborated by the authors (2025).

**Fig. 2:**
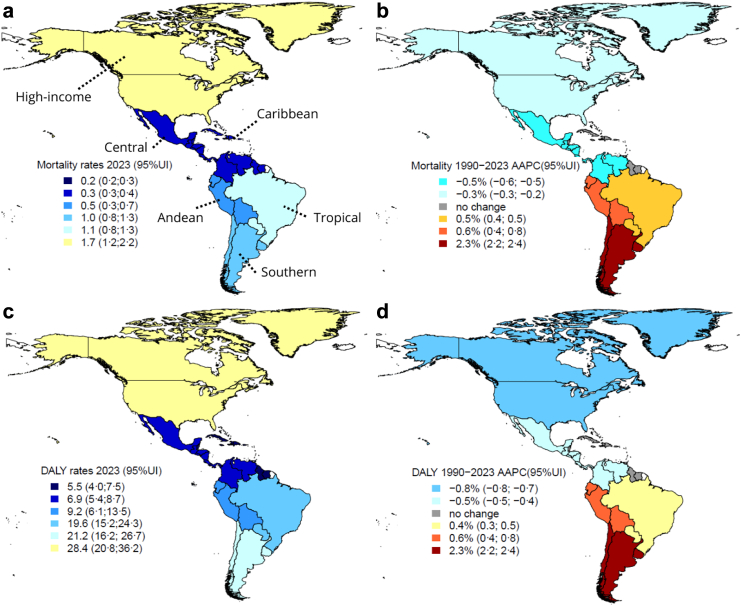
**Age-standardised rates (2023) and average annual percentage change (1990–2023) of (a, b) mortality and (c, d) disability-adjusted life years (DALY) for cancer in women attributable to occupational asbestos exposure by regions in the Americas.** Mortality: age-standardized mortality rates per 100,000; DALY: age-standardized DALY rates per 100,000; AAPC: average annual percentage change; 95% UI: 95% uncertainty interval. High-income: High-income North America; Central: Central Latin America; Andean: Andean Latin America; Tropical: Tropical Latin America; Southern: Southern Latin America. Cancer in women: ovarian, laryngeal and lung cancers and mesothelioma. Elaborated by the authors (2025).

**Fig. 3:**
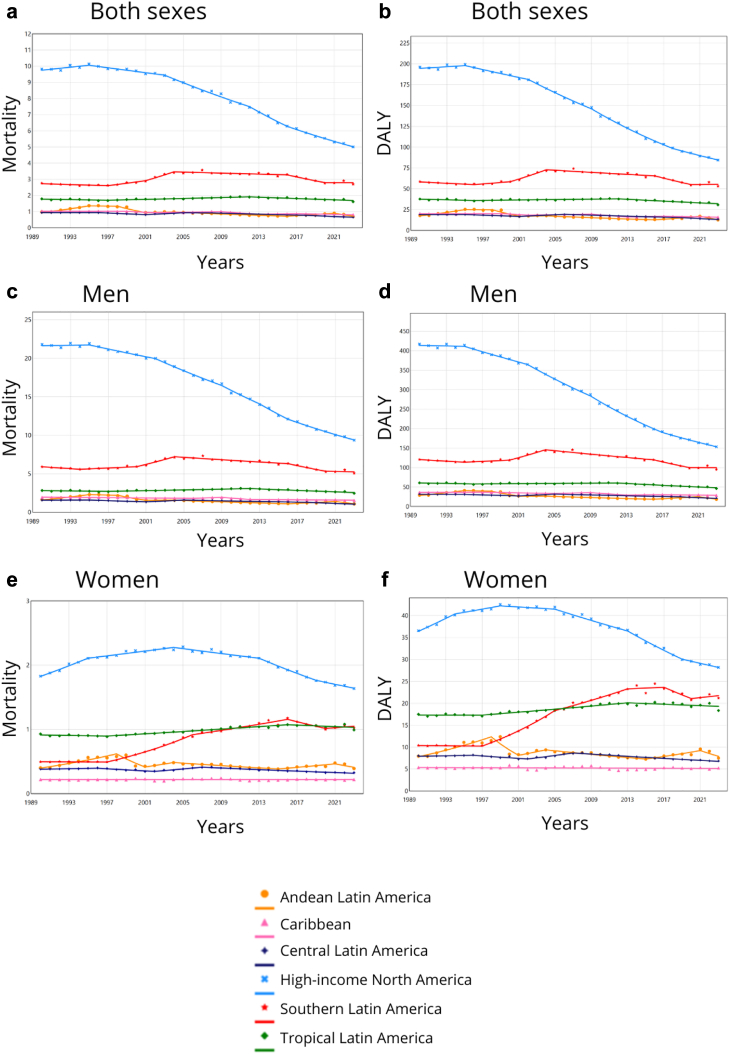
**Trends from joinpoint segmented regression analyses in age-standardized mortality and disability-adjusted life years (DALY) rates for cancer attributable to occupational asbestos exposure in the Americas by sex and regions, 1990–2023.** Mortality: age-standardized mortality rates per 100,000 for (a) both sexes, (c) men, and (e) women; DALY: age-standardized DALY rates per 100,000 for (b) both sexes, (d) men, and (f) women. Points: observed values; continuous lines: estimated values. Cancer in men: laryngeal and lung cancers and mesothelioma. Cancer in women: ovarian, laryngeal and lung cancers and mesothelioma. Elaborated by the authors (2025).

Considering all types of cancer attributable to occupational asbestos, upward trends were observed only in women in Andean (although with lower mortality and DALY estimates), Tropical and Southern Latin America, most notably in the latter, which showed a 2·3% (2·2; 2·4) increase in mortality and DALY rates per year. In Tropical Latin America, rates in women increased between 1997 and 2013, showing a slight annual increase of 1·1% (0·9; 1·3) for mortality and 1·0% (0·8; 1·4) for DALYs. Southern Latin America showed more pronounced increases, with rates peaking between 1997 and 2006, with an annual increase of 7·3% (7·0; 7·6) for mortality and 7·2% (1·5; 7·6) for DALYs. Slower increases occurred between 2006 and 2016, with 2·3% (2·0; 2·7) for mortality, and between 2006 and 2013, with 2·6% (2·3; 7·6) for DALYs (Fig. 1, Fig. 2, Fig. 3 and Table 1, Tables S1 and S5).

**Table 1:** Mortality and disability-adjusted life years (DALY) numbers and rates for cancer attributable to occupational asbestos exposure, by sex and regions in 1990 and 2023, and trends from 1990 to 2023 in the Americas.

	Mortality										DALY									
	1990				2023				1990–2023		1990				2023				1990–2023	
	Number		Rate		Number		Rate		AAPC (%)		Number		Rate		Number		Rate		AAPC (%)	
	95% UI		95% UI		95% UI		95% UI		95% UI		95% UI		95% UI		95% UI		95% UI		95% UI	
High-income North America																				
Both sexes	**37,041·1**		**9·8**		**37,645·7**		**5·1**		***−2·0∗***		**721,635·9**		**196·2**		**623,686·3**		**84·9**		***−2·5∗***	
	27,884·6	46,059·9	7·4	12·3	29,317·1	47,781·1	3·9	6·4	−2·0	−1·9	536,624·6	914,414·1	145·3	249·2	481,846·9	796,787·6	65·6	108·5	−2·5	−2·4
Male	**32,956·4**		**21·8**		**30,778·0**		**9·5**		***−2·5∗***		**644,583·9**		**417·2**		**511,254·7**		**154·4**		***−3·0∗***	
	24,016·6	42,252·5	15·9	28·1	23,156·1	40,472·2	7·1	12·4	−2·5	−2·4	463,263·1	835,815·8	299·6	541·7	378,852·1	681,121·8	114·8	205·8	−3·0	−2·9
Female	**4084·7**		**1·8**		**6867·7**		**1·7**		***−0·3∗***		**77,052·0**		**36·6**		**112,431·6**		**28·4**		***−0·8∗***	
	2970·6	5101·5	1·3	2·3	4827·7	8936·6	1·2	2·2	−0·3	−0·2	57,394·8	96,499·5	27·3	46·1	81,436·3	144,510·8	20·8	36·2	−0·8	−0·7
Central Latin America																				
Both sexes	**708·7**		**0·9**		**1728·8**		**0·7**		***−1·1∗***		**15,354·6**		**19·3**		**35,849·6**		**13·4**		***−1·1∗***	
	525·7	924·8	0·7	1·2	1323·3	2275·5	0·5	0·9	−1·1	−1·0	11,557·9	19,936·4	14·4	25·1	27,873·4	46,380·7	10·4	17·3	−1·2	−1·0
Male	**555·0**		**1·6**		**1257·5**		**1·1**		***−1·2∗***		**11,949·4**		**31·7**		**25,821·1**		**21·2**		***−1·2∗***	
	378·6	764·8	1·1	2·2	889·7	1742·8	0·8	1·5	−1·2	−1·1	8346·3	16,442·0	21·9	43·7	18,509·3	35,415·0	15·1	29·2	−1·3	−1·1
Female	**153·8**		**0·4**		**471·3**		**0·3**		***−0·5∗***		**3405·2**		**8·0**		**10,028·5**		**6·9**		***−0·5∗***	
	121·9	188·2	0·3	0·5	367·9	599·2	0·3	0·4	−0·6	−0·5	2728·9	4149·2	6·4	9·8	7868·0	12,791·7	5·4	8·7	−0·5	−0·4
Caribbean																				
Both sexes	**279·9**		**1·1**		**478·6**		**0·8**		***−0·7∗***		**5562·0**		**21·1**		**9459·6**		**16·7**		***−0·7∗***	
	198·7	384·3	0·8	1·5	338·5	686·0	0·6	1·2	−0·8	−0·6	4067·0	7473·7	15·4	28·4	6725·9	13,272·1	11·9	23·4	−0·8	−0·6
Male	**248·7**		**2·0**		**408·3**		**1·6**		***−0·6∗***		**4793·8**		**38·1**		**7829·2**		**30·0**		***−0·7∗***	
	163·4	352·6	1·3	2·9	271·2	597·8	1·1	2·4	−0·8	−0·5	3179·9	6662·8	25·3	53·2	5269·1	11,391·0	20·2	43·6	−0·8	−0·6
Female	**31·3**		**0·2**		**70·3**		**0·2**		0·0		**768·3**		**5·6**		**1630·4**		**5·5**		−0·1	
	24·0	41·5	0·2	0·3	51·1	96·1	0·2	0·3	−0·2	0·2	594·0	996·5	4·3	7·2	1199·5	2236·0	4·0	7·5	−0·3	0·1
Andean Latin America																				
Both sexes	**177·5**		**1·0**		**533·5**		**0·9**		***−0·3∗***		**3459·3**		**18·0**		**9845·3**		**15·4**		***−0·4∗***	
	118·3	244·2	0·7	1·4	377·3	729·0	0·6	1·2	−0·5	−0·2	2350·7	4786·7	12·2	24·9	6994·4	13,364·5	11·0	20·9	−0·6	−0·2
Male	**138·1**		**1·7**		**369·2**		**1·3**		***−0·7∗***		**2641·6**		**29·3**		**6686·5**		**22·8**		***−0·8∗***	
	87·3	202·3	1·0	2·4	239·3	536·8	0·9	1·9	−0·9	−0·4	1620·7	3886·4	18·4	42·9	4384·9	9766·3	14·8	33·3	−1·0	−0·5
Female	**39·4**		**0·4**		**164·3**		**0·5**		*0·6∗*		**817·7**		**8·0**		**3158·8**		**9·2**		*0·6∗*	
	25·2	59·4	0·3	0·6	108·6	244·2	0·3	0·7	0·4	0·8	538·9	1235·4	5·2	12·0	2078·1	4606·6	6·1	13·5	0·4	0·8
Tropical Latin America																				
Both sexes	**1431·6**		**1·8**		**4504·4**		**1·7**		***−0·1∗***		**34,093·3**		**37·7**		**87,985·5**		**32·9**		***−0·4∗***	
	1086·5	1826·7	1·4	2·3	3548·1	5617·6	1·4	2·2	−0·2	−0·03	26,028·4	43,616·9	28·7	48·0	71,073·3	109,031·7	26·6	40·8	−0·5	−0·3
Male	**1048·5**		**2·8**		**2910·4**		**2·6**		***−0·2∗***		**25,891·1**		**60·9**		**58,761·3**		**49·8**		***−0·8∗***	
	712·7	1426·6	1·9	3·9	2072·4	3886·9	1·9	3·5	−0·3	−0·1	17,574·6	35,188·4	41·4	82·6	42,627·9	77,780·3	36·0	65·9	−1·0	−0·5
Female	**383·1**		**0·9**		**1594·0**		**1·1**		*0·5∗*		**8202·1**		**17·5**		**29,224·3**		**19·6**		*0·4∗*	
	314·2	464·1	0·8	1·1	1171·8	2026·4	0·8	1·3	0·4	0·5	6895·8	9944·2	14·6	21·2	22,523·6	36,267·2	15·2	24·3	0·3	0·5
Southern Latin America																				
Both sexes	**1266·1**		**2·8**		**2650·8**		**2·7**		0·1		**27,522·5**		**58·7**		**50,650·3**		**53·1**		***−0·2∗***	
	866·9	1735·2	1·9	3·8	2016·3	3430·8	2·1	3·5	−0·04	0·2	18,708·5	37,575·2	40·0	80·0	38,471·6	66,101·0	40·4	69·3	−0·3	−0·04
Male	**1136·4**		**5·9**		**2072·9**		**5·1**		***−0·3∗***		**24,806·2**		**121·0**		**39,435·8**		**95·2**		***−0·6∗***	
	732·0	1607·8	3·8	8·4	1405·0	2854·8	3·5	7·0	−0·5	−0·2	15,950·5	34,798·5	78·1	169·8	26,436·2	55,002·8	64·1	132·6	−0·7	−0·5
Female	**129·7**		**0·5**		**577·9**		**1·0**		*2·3∗*		**2716·3**		**10·4**		**11,214·5**		**21·2**		*2·3∗*	
	102·3	168·4	0·4	0·6	434·5	741·0	0·8	1·3	2·2	2·4	2161·8	3480·9	8·3	13·3	8555·2	14,135·3	16·2	26·7	2·2	2·4

Number: number of deaths and DALYs for all ages. Rate: age-standardized rate per 100,000; AAPC: average annual percentage change of rates from 1990 to 2023; 95% UI: 95% uncertainty interval; ∗significance: AAPC <> 0 and p-value <0·05. Bold-italic: shows downward trends; Italic: shows upward trends. Male: laryngeal and lung cancers and mesothelioma. Female: ovarian, laryngeal and lung cancers and mesothelioma. Elaborated by the authors (2025).

Among the cancers attributable to occupational asbestos, lung cancer had the highest age-standardized mortality and DALY rates in all regions in 2023, followed by mesothelioma, ovarian and laryngeal cancer (Table S1). For lung cancer in men, only decreasing trends were observed between 1990 and 2023, but in Tropical and Southern Latin America these reductions were milder, respectively, 0·3% (−0·4; −0·2) and 0·4% (−0·6; −0·3) in mortality and 0·8% (−0·9; −0·7) and 0·7% (−0·8; −0·6) in DALY rates per year (Table S5). In Southern Latin America, Uruguay had the highest rates, with 6·4 (4·0; 9·3) deaths and 121·0 (75·0; 177·9) DALYs, followed by Argentina, with 5·1 (3·2; 7·2) deaths and 93·8 (58·4; 135·9) DALYs in 2023 (Tables S3 and S4). Upward trends were observed for lung cancer in women in the Caribbean, Andean, Tropical and Southern Latin America. Rates grew the fastest in the Southern Latin America, 2·8% (2·7; 2·9) for mortality and 2·7% (2·6; 2·8) for DALYs from 1990 to 2023, and showed a more marked increase between 1998 and 2005 of 8·0% (0·5; 8·7) in mortality and between 2000 and 2005 of 8·5% (6·8; 9·9) in DALYs per year (Table S5). Argentina had the highest rates for lung cancer in women, with 0·7 (0·5; 1·1) for mortality and 14·5 (9·3; 20·8) for DALYs. Brazil ranked second, with 0·7 (0·4; 0·9) for mortality and 10·9 (7·0; 14·9) for DALYs (Tables S3 and S4).

Men presented upward trends only for mesothelioma, with an annual increase of 0·8% (0·6; 0·9) in mortality and 0·6% (0·5; 0·8) in DALYs in Southern Latin America, 0·6% (0·3; 1·1) for both mortality and DALYs in Andean Latin America, and 0·2% (0·1; 0·3) only for mortality in Central and Tropical Latin America (Table S6). In Southern and Andean Latin America, the highest rates for mesothelioma in men in 2023 were observed in Argentina (mortality: 0·6 [0·5; 0·6]; DALY: 13·2 [11·6; 15·1]), Bolivia (mortality: 0·5 [0·3; 0·7]; DALY: 11·1 [6·3; 16·1]) and Uruguay (mortality: 0·5 [0·4; 0·5]; DALY: 11·3 [9·7; 13·1]) (Tables S3 and S4). Among women, increasing age-standardized mortality and DALY rates for mesothelioma were observed in Andean and Southern Latin America, but grew faster in the latter, averaging 2·3% (2·2; 2·5) in both mortality and DALYs per year. Also, the region showed steeper increases of 8·2% (7·5; 9·1) in mortality and 8·3% (7·5; 9·3) in DALYs per year from 1997 to 2005 (Table S5). Argentina had the highest mortality and DALY rates for mesothelioma in women, respectively 0·3 (0·2; 0·3) and 7·0 (5·9; 8·2) (Tables S3 and S4).

Laryngeal cancer rates in men attributable to occupational asbestos declined in all regions except the Caribbean, which showed a stable trend from 1990 to 2023. The less pronounced reductions were observed in Tropical Latin America, with an annual decrease of only 0·3% (−0·4; −0·2) in mortality and 0·5% (−0·6; −0·5) in DALYs (Table S6).

Ovarian cancer attributable to occupational asbestos showed upward trends in Central, Caribbean, Andean and Southern Latin America. Notable increasing rates were observed in the Southern region, at 1·1% (1·0; 1·3) per year in both mortality and DALYs, with the highest rates observed in Argentina (mortality: 0·2 [0·1; 0·4]; DALY: 4·7 [2·2; 7·6]) (Tables S3 and S4). Andean Latin America showed more marked increases, with 2·5% (2·3; 2·6) in mortality and 2·4% (2·3; 2·6) in DALYs per year, but the estimated numbers and rates were lower (Tables S1 and S5).

Mortality and DALY rates for cancer attributable to occupational asbestos exposure increased with age, but decreasing trends were observed in most regions over the period and across birth cohorts. In contrast, the Southern Latin America region showed increasing trends in women for more recent periods and birth cohorts (Figures S5, S6 and S7).

**Fig. 4:**
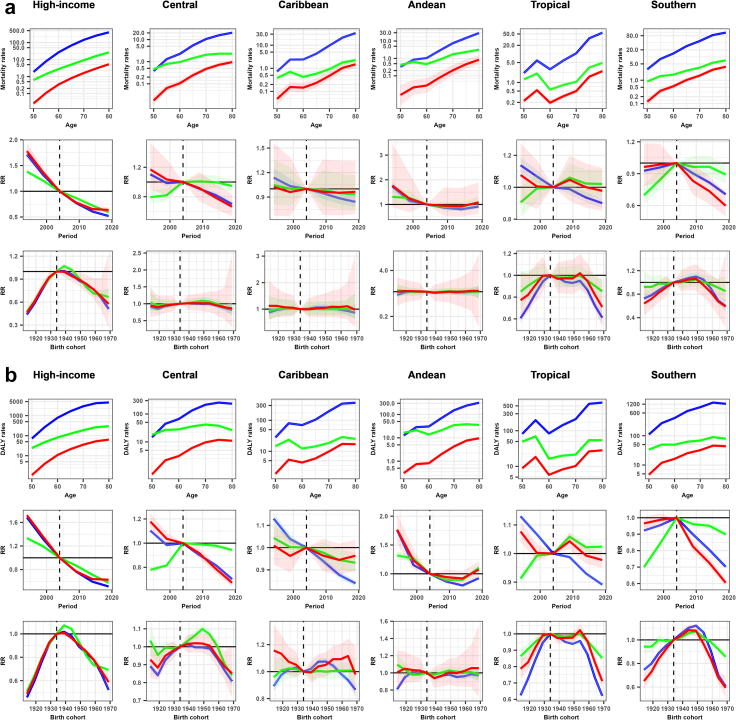
**Age-period-cohort model-derived estimates of (a) mortality and (b) disability-adjusted life years (DALY) age-specific groups rates for each cancer in men attributable to occupational asbestos exposure between 1994 and 2023 by regions in the Americas.** Red: laryngeal cancer; blue: lung (trachea, bronchi and lungs) cancer; green: mesothelioma. Shaded areas correspond to uncertainty interval 95%. High-income: High-income North America; Central: Central Latin America; Andean: Andean Latin America; Tropical: Tropical Latin America; Southern: Southern Latin America. Mortality rates: age-specific groups rates on a logarithmic scale; DALY rates: age-specific groups rates on a logarithmic scale; RR: rate ratio. Age: age effects on mortality and DALY rates; Period: period effects expressed as RR of mortality and DALY rates; Birth cohort: cohort effects expressed as RR of mortality and DALY rates. Elaborated by the authors (2025).

**Fig. 5:**
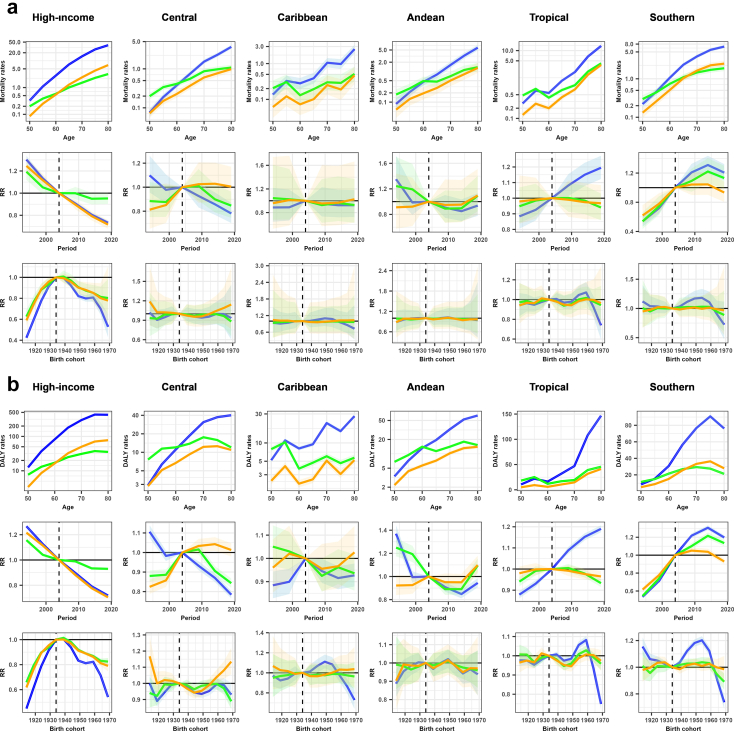
**Age-period-cohort model-derived estimates of (a) mortality and (b) disability-adjusted life years (DALY) age-specific groups rates for each cancer in women attributable to occupational asbestos exposure between 1994 and 2023 by regions in the Americas.** Orange: ovarian cancer; blue: lung (trachea, bronchi and lungs) cancer; green: mesothelioma. Shaded areas correspond to uncertainty interval 95%. High-income: High-income North America; Central: Central Latin America; Andean: Andean Latin America; Tropical: Tropical Latin America; Southern: Southern Latin America. Mortality rates: age-specific groups rates on a logarithmic scale; DALY rates: age-specific groups rates on a logarithmic scale; RR: rate ratio. Age: age effects on mortality and DALY rates; Period: period effects expressed as RR of mortality and DALY rates; Birth cohort: cohort effects expressed as RR of mortality and DALY rates. Elaborated by the authors (2025).

Age-period-cohort modelling revealed decreasing lung cancer mortality and DALY rates for men in all regions. High-income North America showed the strongest declines in RR, dropping from 0·78 (95% UI 0·73; 0·84) to 0·51 (0·51; 0·52) since 2009 and from 0·98 (0·97; 0·98) to 0·51 (0·46; 0·57) since the 1939 birth cohort onwards. Conversely, RR for lung cancer mortality and DALYs in women increased up to 1·18 (1·11; 1·25) and 1·31 (1·20; 1·44), respectively, in Tropical and Southern Latin America since 2009 (Figs. 4 and 5; Tables S7 and S8).

Increases in the RR for mesothelioma in men were only observed for DALYs, with 1·06 (1·01; 1·10) in Andean Latin America in 2019–23. For women, RR for mesothelioma mortality and DALYs increased by 1·22 (1·06; 1·40) in 2014–18 in Southern Latin America (Figs. 4 and 5; Tables S7 and S8).

The most decreasing RR for laryngeal cancer in men were observed in High-income North America, Central, and Southern Latin America, ranging from 0·60 (0·50; 0·72) to 0·67 (0·64; 0·70) in 2019–23. Decreases in RR for the youngest birth cohort (1969) were observed most notably for DALYs in High-income North America, Tropical and Southern Latin America, ranging from 0·59 (0·52; 0·66) to 0·71 (0·67; 0·75) (Fig. 4 and Table S7).

The High-income North America region has seen reductions from 0·90 (0·86; 0·94) to 0·70 (0·70; 0·71) in RR for mortality and DALYs for ovarian cancer since 2009. A slight increase in RR for DALYs was observed in Central Latin America, 1·04 (1·01; 1·08) in 2014–18, and in Southern Latin America, 1·05 (1·02; 1·09) in 2009–13 (Fig. 5 and Table S8).

## Discussion

Despite decades of regulation, asbestos continues to exert a significant burden in parts of the Americas, especially among populations previously considered at lower risk, such as women. Lung cancer had the highest mortality and DALY rates in all regions in 2023, followed by mesothelioma, ovarian and laryngeal cancer. High-income North America showed the strongest declines for cancer in men and women between 1990 and 2023 but rates remained the highest among all regions in 2023 in the Americas. Conversely, pronounced increases in mortality and DALY rates were observed for lung cancer and mesothelioma in women in Tropical and Southern Latin America. Slightly higher mortality and DALY rates were observed for mesothelioma in men in Andean, Central and Southern Latin America, and for ovarian cancer in Central and Southern Latin America.

Although the current study showed a decreasing burden of cancer attributable to occupational asbestos exposure in High-income North America, the United States (U.S.) had the highest numbers of mortality and DALY in 2023 in the Americas, followed by Canada. The burden of occupational cancer is generally higher in countries with a higher SDI.^4^ The U.S. started banning some processes using asbestos in 1973. In 1989, the Asbestos Ban and Phase-Out Rule introduced ten-year bans on the import, processing, manufacturing, and distribution of asbestos-containing products. However, the regulation was overturned in 1991. As a result, over 300,000 tons of asbestos were used, mostly from Canadian mines, and large quantities of asbestos-containing construction products and other items were incorporated into the U.S. infrastructure. Finally, an effective phase-out of all types of asbestos was proclaimed only in 2024. Greenland and Canada banned asbestos use in 2010 and 2018 respectively^10^^,^^29^(Figure S5). Approximately 235,000 workers were estimated to have been exposed to occupational asbestos in Canada in 2016. Most were employed in the construction and building maintenance sectors, reflecting a shift from high historical exposures, such as mining, to risks associated with asbestos-containing materials in older buildings.^30^ Mortality rates from mesothelioma are increasing in most industrialized countries, but the rate of increase has slowed only in the few that began reducing asbestos use decades ago. Indeed, continued reporting of mesothelioma deaths in people under 55 years suggests persistent occupational and environmental exposures.^31^ Countries where asbestos has been banned can exhibit a sustained epidemic of asbestos-related diseases, as large amounts of asbestos remain as a legacy of past construction practices in structures such as schools, homes, commercial buildings, and industries. It's important to identify and mark these locations to prevent the exposure of workers and residents during maintenance or demolition work. Deterioration, erosion, or breakage of materials can release asbestos fibers into the air, soil, and water, exposing an entire community. The handling of asbestos-containing waste must also be regulated through specialized training and licensing programs.^32^ Moreover, cumulative exposures^33^ and the long latency period for mesothelioma (more than 15 years with a median of 32 years) and lung cancer attributable to asbestos (10–40 years)^1^ may still contribute for higher mortality and DALY rates in the future. Despite an overall decrease in asbestos exposure, there is still an increasing burden mainly due to population growth and aging.^34^ This trend underscores the need for ongoing surveillance and tailored healthcare resources for aging populations with historical exposure.

Our study showed some increasing trends in cancer attributable to occupational asbestos in Tropical and Southern Latin America. Because of more strict regulations, European countries began to buy asbestos in South American industries, which exponentially increased the production in that region in the last 30 years.^35^ Also, South Africa exported amphiboles, which have a higher carcinogenic potential, to Latin American countries between 1980 and 2003, primarily to Argentina, Brazil, Colombia, and Mexico.^36^^,^^37^ Brazil became the most influential asbestos producer in South America in the 1970s.^38^ The country banned the use of amphiboles in 1995, but chrysotile continued to be used until 2017,^39^ when it was also banned by decision of the Federal Supreme Court. However, the extraction of chrysotile asbestos still occurs in the state of Goiás under the protection of a state law, although for export purposes only^40^ (Figure S8). Even so, Brazil ranked fourth in world asbestos mining in 2023, with a production of 190 thousand tons.^9^ Although there was a decline in mortality rates and DALYs from laryngeal and lung cancer attributable to occupational exposure to asbestos in Brazil between 1990 and 2019, increases in burden were observed in North and Northeast regions of the country.^41^^,^^42^

In Southern Latin America, Argentina, Chile and Uruguay banned asbestos between 2001 and 2002, but some industries continued using asbestos in Argentina until 2014.^10^ In the other regions evaluated, only Colombia and Honduras (the latter with some exceptions) prohibited the use of asbestos.^10^ For decades, Colombia was one of the most important asbestos producers in the region, with a national production and consumption of 25,200 tons in 2012. After a series of demands from the public society, asbestos ban legislation was passed in 2019^38^ (Figure S8).

The current study found higher rates of cancer attributable to occupational asbestos in men, although with mostly decreasing trends. However, increasing rates of lung cancer and mesothelioma were observed in women in Tropical and Southern Latin America. Men are more commonly found in occupations that are high risk for asbestos exposure, such as construction, mining and demolition. However, disadvantages for female workers range from biological susceptibility to workplace inequalities and environmental exposures. Women's vulnerability to harmful agents can vary based on their reproductive cycles, and chemical exposures in female workers are increasing dramatically and are often underestimated.^8^ Overall, women are the majority in textile industries, including the ones that produce protective clothing with asbestos materials.^8^ In addition, personal safety equipment has been traditionally designed for the male body and therefore may fit female workers poorly, leading to reduced protection and increased risk of chemical exposure.^8^ Targeted occupational health interventions for female workers and increased surveillance are needed in industries where women are now significantly exposed.

Mesothelioma cases with environmental exposure to asbestos or with unknown exposure are more frequent among women.^43^ Although asbestos is considered primarily an occupational hazard, environmental contamination through the air can reach many kilometers from a mining site, which could explain the correlation of mesothelioma incidence in women with areas with high asbestos mining and consumption.^44^ In the domestic context, exposures were reported through water tanks and asbestos roof tiles.^44^ In addition, family members of the worker could be exposed to considerable amounts of asbestos fibers brought home on their work clothes, which are often cleaned by the wife or daughters, with mesothelioma incidence being described in such cases.^45^

The asbestos ban has been a slow process for many countries. Multiple commercial interests, such as the low price and easy accessibility of asbestos, high demand from the construction sector, and the industry's fierce propaganda, are still delaying the total ban of asbestos.^32^ In this sense, we highlight the concept of Gilmore et al. (2023) on the commercial determinants of health, which states that industry interests are placed above public health and political agents align with the industry to interfere with environmental and occupational safety legislation.^46^ Also, occupational exposure to carcinogens disproportionately affects certain groups of workers, such as the young and elderly, migrants, women and informal workers, who may face greater exposure due to ineffective or non-existent protective measures.^8^ Fortunately, safer and cost-effective alternative materials have been adopted by countries where asbestos has been banned, such as polyvinyl alcohol fibers and concrete fabricated with cement, sand and gravel. In Brazil, fiber-cement is made from polypropylene and cellulose fibers. Roof tiles can also be fabricated using plant fibers, such as jute, hemp, sisal, palm nut, coconut, and wood pulp.^32^

Most previous studies analyzed the occupational cancer burden longitudinally or did not focus on a specific carcinogen. The main strength of our study lies in age-period-cohort analyses of mortality and DALY rates. Such effects can be confounded and their separate assessment provides a clearer understanding of disparities in cancer burden among age-groups through time, which allows better targeting of potential interventions. However, our findings should be interpreted in light of certain limitations. Firstly, the association of other risk factors in the workplace, such as smoking and other chemical products, may make it difficult to establish a causal relationship between cancer and asbestos alone. Future studies that assess other risk factors and possible synergistic effects would be useful for a better understanding of the relationship between asbestos and cancer. Moreover, for smokers, the effect of interaction with asbestos exposure is more than additive for lung cancer.^47^ Upward trends in female lung cancer rates observed in our study may also reflect increased smoking among women,^48^ while mesothelioma is much more specific to asbestos exposure.^2^ Exposure risks depend not only on the sources of asbestos production, but also on the individual and collective protection measures adopted in the work environment,^8^ which can significantly impact the development of occupational cancer. Other important limitations may affect the burden estimate, such as the long cancer latency periods attributable to asbestos^1^ and some diagnostic difficulties such as with ovarian cancer. Studies have identified asbestos fibers in ovaries, but the association between asbestos exposure and ovarian cancer has still been reported as conflicting, both due to the small number of women exposed to asbestos and the possibility of misclassifying peritoneal mesothelioma as ovarian cancer. However, cases of misclassification have decreased in recent years due to the development of new immunohistochemical diagnostic methods.^49^^,^^50^ Female participation in the labor market and therefore chemical exposures are increasing, but are often underreported, especially in the informal sector and in low and middle-income countries.^8^ It is also important to mention that our study did not report race or ethnicity, since estimates of occupational carcinogens are not stratified by these covariates. All these limitations, associated with wide variations in the quality of national data, may lead to underreporting of cancer attributable to occupational asbestos.^51^

### Conclusions

Our study stands out as a comprehensive approach to the burden of cancer attributable to occupational asbestos exposure in the Americas. The results showed decreasing trends in cancer rates that were most evident in North America, and highlighted regional and sex inequalities, with higher cancer rates for women in South America. Although the use of asbestos has been banned in some countries, mining activities still occur due to legal disputes and commercial interests. Therefore, it is essential to strengthen occupational health regulatory measures through stricter enforcement of asbestos bans, standardized occupational health protocols, and also harmonization of regional labor policies across Latin America.

Despite regulatory advances, legacy exposures and ongoing asbestos use persist in parts of Latin America, reinforcing the need for stricter occupational health policies and asbestos bans. The findings underscore the shifting epidemiology of asbestos-related cancers and call for targeted prevention efforts, improved surveillance, and gender-responsive occupational protections.^35^

Finally, our conclusions not only outline the impact of occupational asbestos exposure in the Americas, but also provide support for future studies to assess its environmental repercussion on the general population. The increase in mesothelioma and lung cancer rates among women in Tropical and Southern Latin America is a sentinel health event. It strongly suggests that the burden of disease is no longer confined to the workplace and has expanded to include significant para-occupational and environmental exposures.

## Contributors

Please see Appendix 1 for more detailed information about individual author contributions to the research, divided into the following categories: managing the overall research enterprise; writing the first draft of the manuscript; primary responsibility for applying analytical methods to produce estimates; primary responsibility for seeking, cataloguing, extracting, or cleaning data; designing or coding figures and tables; providing data or critical feedback on data sources; developing methods or computational machinery; providing critical feedback on methods or results; drafting the manuscript or revising it critically for important intellectual content; and managing the estimation or publications process.

## Editor note

The Lancet Group takes a neutral position with respect to territorial claims in published maps, figures, tables, and institutional affiliations.

## Data sharing statement

This study follows the Guidelines for Accurate and Transparent Health Estimates Reporting (GATHER). To download citations and metadata for the input data sources used in the GBD 2023 analyses presented in this study, please visit the GBD 2023 Sources Tool (https://ghdx.healthdata.org/gbd-2023/sources).

## AI use

No artificial intelligence tool was used in the preparation of any part of the manuscript.

## Declaration of interests

**GBD 2023 Americas Occupational Exposure to Asbestos Declarations:** X Fan reports leadership or fiduciary role in other board, society, committee or advocacy group, paid or unpaid as Director of the Occupational Medicine Specialists of Canada (until December 2025) and Unpaid volunteer role with national specialty society for occupational medicine in Canada; Other financial or non-financial interests an occupational physician in a subnational ministry of labour in Canada (province of Alberta). Contributions to this work are personal and do not constitute official work of the Government of Alberta. L M Force reports support for the present manuscript from The Gates Foundation; Grants or licenses from St. Jude Children's Research Hospital, St. Baldrick's Foundation, Conquer Cancer Foundation, NIH Loan Repayment Program; and Leadership or fiduciary role in other board, society, committee or advocacy group, unpaid from the Lancet Oncology International Advisory Board. J A Singh reports consulting fees from ROMTech, Atheneum, Clearview healthcare partners, Yale, Hulio, Horizon Pharmaceuticals/DINORA, ANI/Exeltis, USA Inc., Frictionless Solutions, Schipher, Crealta/Horizon, Medisys, Fidia, PK Med, Two labs Inc., Adept Field Solutions, Clinical Care options, Putnam associates, Focus forward, Navigant consulting, Spherix, MedIQ, Jupiter Life Science, UBM LLC, Trio Health, Medscape, WebMD, and Practice Point communications; the National Institutes of Health; and the American College of Rheumatology]; Payment or honoraria for lectures, presentations, speakers bureaus, manuscript writing or educational events from Simply Speaking; Support for attending meetings and/or travel from OMERACT; Participation on the FDA Arthritis Advisory Committee; Leadership or fiduciary role in other board, society, committee or advocacy group, paid or unpaid as Past steering committee member of the OMERACT, an international organization that develops measures for clinical trials and receives arm's length funding from 12 pharmaceutical companies; Stock or stock options in Atyr pharmaceuticals, Atai life sciences, Kintara therapeutics, Intelligent Biosolutions, Acumen pharmaceutical, TPT Global Tech, Vaxart pharmaceuticals, Atyu biopharma, Adaptimmune Therapeutics, GeoVax Labs, Pieris Pharmaceuticals, Enzolytics Inc., Seres Therapeutics, Tonix Pharmaceuticals Holding Corp., Aebona Pharmaceuticals, and Charlotte's Web Holdings, Inc and previously owned stock options in Amarin, Viking and Moderna pharmaceuticals; outside the submitted work. S Straube reports grants or contracts from the Workers' Compensation Board (Alberta) and the Alberta Medical Association, grants paid to institution; Payment or honoraria for lectures, presentations, speakers bureaus, manuscript writing or educational events from the Occupational Medicine Specialists of Canada; Leadership or fiduciary role in other board, society, committee or advocacy group, paid or unpaid from Alberta Medical Association as Past President of the Section of Occupational Medicine and President of the Section of Occupational Medicine, SN Comprehensive Clinical Medicine as an editorial board member, and M.S.I. Foundation as a board member; outside the submitted work.

## Appendix A Supplementary data

Appendix 1

PubMed
