# Supplementary material for: Burden of cancer attributable to occupational asbestos exposure in the Americas, 1990–2023: an analysis using the Global Burden of Disease Study 2023
Source: Lancet Reg Health Am. 2026 Apr 2;58:101463. doi: 10.1016/j.lana.2026.101463 (PMC13085095; doi:10.1016/j.lana.2026.101463)
Supplement: Supplementary Material [file mmc1.pdf]

SUPPLEMENTARY MATERIAL

LIST OF FIGURES

LIST OF FIGURES	Page
**Figure S1.** GBD 2023 regions and respective countries in the Americas.	1
**Figure S2.** Socio-demographic index (SDI) quintiles (a,b) and ranges in age-standardised (c,d) mortality and (e,f) disability-adjusted life years (DALY) rates for cancer in men attributable to occupational asbestos exposure by countries in the Americas, 1990 and 2023.	2
**Figure S3.** Socio-demographic index (SDI) quintiles (a,b) and ranges in age-standardised (c,d) mortality and (e,f) disability-adjusted life years (DALY) rates for cancer in women attributable to occupational asbestos exposure by countries in the Americas, 1990 and 2023.	3
**Figure S4.** Linear regression for country-level Socio-demographic index (SDI) values and age-standardised mortality and disability-adjusted life years (DALY) rates for cancer in both sexes attributable to occupational asbestos exposure in the Americas, 2023.	4
**Figure S5.** Mortality and disability-adjusted life years (DALY) age-specific groups rates for cancer in men and women attributable to occupational asbestos exposure, within each period by age-groups in the regions of the Americas, 1994-2023.	5
**Figure S6.** Mortality and disability-adjusted life years (DALY) age-specific groups rates for cancer in men and women attributable to occupational asbestos exposure, within each age-group by period in the regions of the Americas, 1994-2023.	6
**Figure S7.** Mortality and disability-adjusted life years (DALY) age-specific groups rates for cancer in men and women attributable to occupational asbestos exposure, within each age-group by birth cohorts in the regions of the Americas, 1994-2023.	7
**Figure S8.** Country-level asbestos policy timelines and mortality and disability-adjusted life years (DALYs) numbers and rates for cancer in both sexes attributable to occupational asbestos exposure in 1990 and 2023 in the regions of the Americas.	8

LIST OF TABLES

LIST OF TABLES	Page
**Table S1.** Mortality and disability-adjusted life years (DALYs) numbers and rates for cancer attributable to occupational asbestos exposure, by sex and regions, in 2023 in the Americas.	11
**Table S2.** Fit for the age-period-cohort models on mortality and disability-adjusted life years (DALY) age-specific groups rates for each cancer attributable to occupational asbestos exposure by sex and regions in the Americas, 1994 to 2023.	14

Table S3. Mortality numbers and rates for cancer attributable to occupational asbestos exposure, by sex, cancer type and country, in 1990 and 2023 in the Americas. 17

Table S4. Disability-adjusted life years (DALY) numbers and rates for cancer attributable to occupational asbestos exposure, by sex, cancer type and country, in 1990 and 2023 in the Americas. 25

Table S5. Trends in age-standardized mortality and disability-adjusted life years (DALYs) rates for cancer in women attributable to occupational asbestos exposure between 1990 and 2023 by regions in the Americas. 33

Table S6. Trends in age-standardized mortality and disability-adjusted life years (DALYs) rates for cancer in men attributable to occupational asbestos exposure between 1990 and 2023 by regions in the Americas. 38

Table S7. Results from the age-period-cohort analysis of mortality and disability-adjusted life years (DALY) age-specific groups rates for each cancer in men attributable to occupational asbestos exposure in the Americas regions from 1994 to 2023. 44

Table S8. Results from the age-period-cohort analysis of mortality and disability-adjusted life years (DALY) age-specific groups rates for each cancer in women attributable to occupational asbestos exposure in the Americas regions from 1994 to 2023. 49

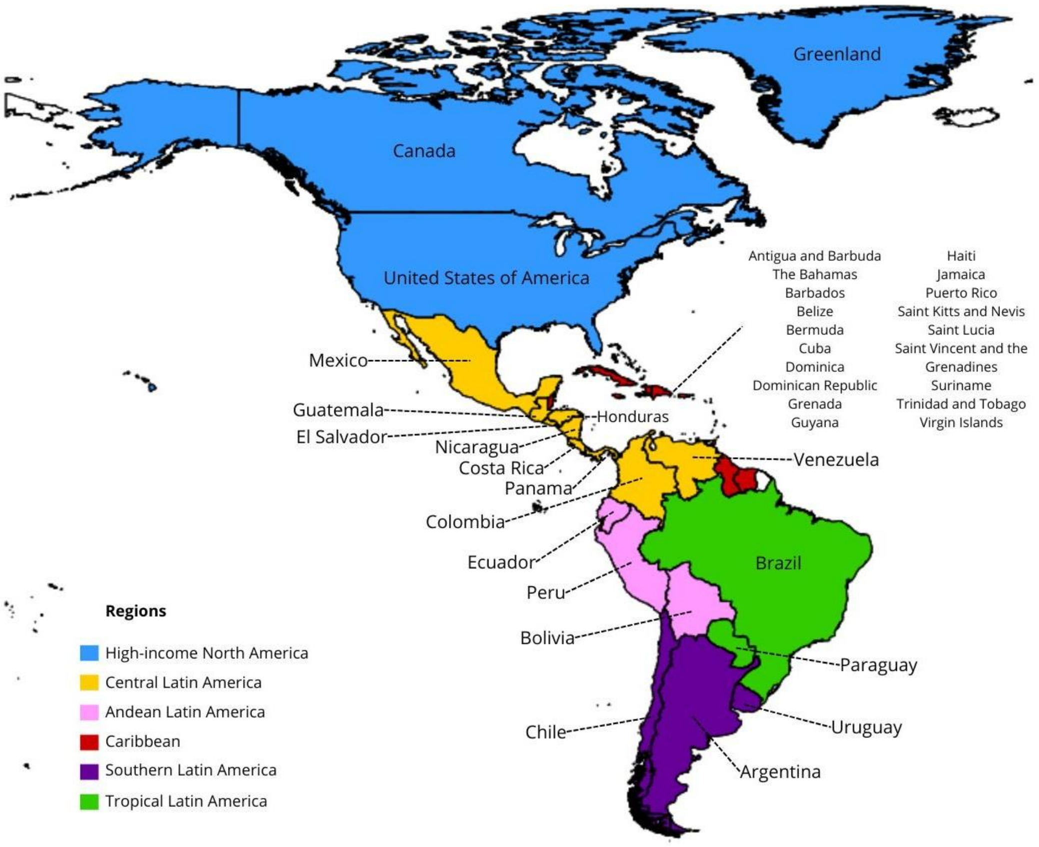

Figure S1. GBD 2023 regions and respective countries in the Americas.
Elaborated by the authors (2025).

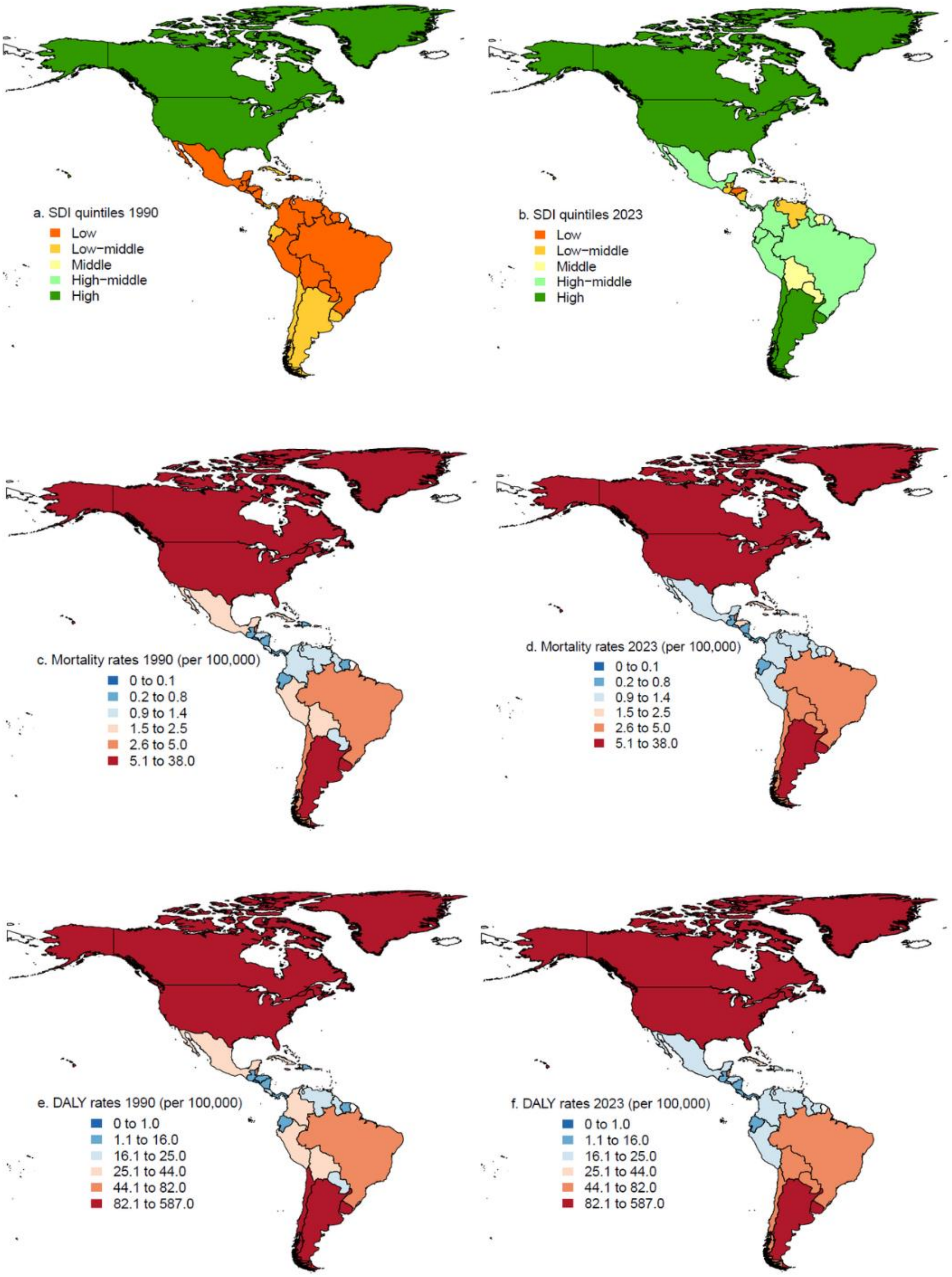

Figure S2. Socio-demographic index (SDI) quintiles (a,b) and ranges in age-standardised (c,d) mortality and (e,f) disability-adjusted life years (DALY) rates for cancer in men attributable to occupational asbestos exposure by countries in the Americas, 1990 and 2023.

SDI quintiles are based on GBD 2023 cutoffs. Low SDI: 0 to 53.16; Low-middle SDI: 53.16 to 63.05; Middle SDI: 63.05 to 67.67; High-middle SDI: 67.67 to 71.88; and High SDI: 71.88 to 100.

Mortality: age-standardized mortality rates per 100,000; DALY: age-standardized DALY rates per 100,000; rates are presented in ranges.

Cancer in men: laryngeal and lung cancers and mesothelioma.

Elaborated by the authors (2025).

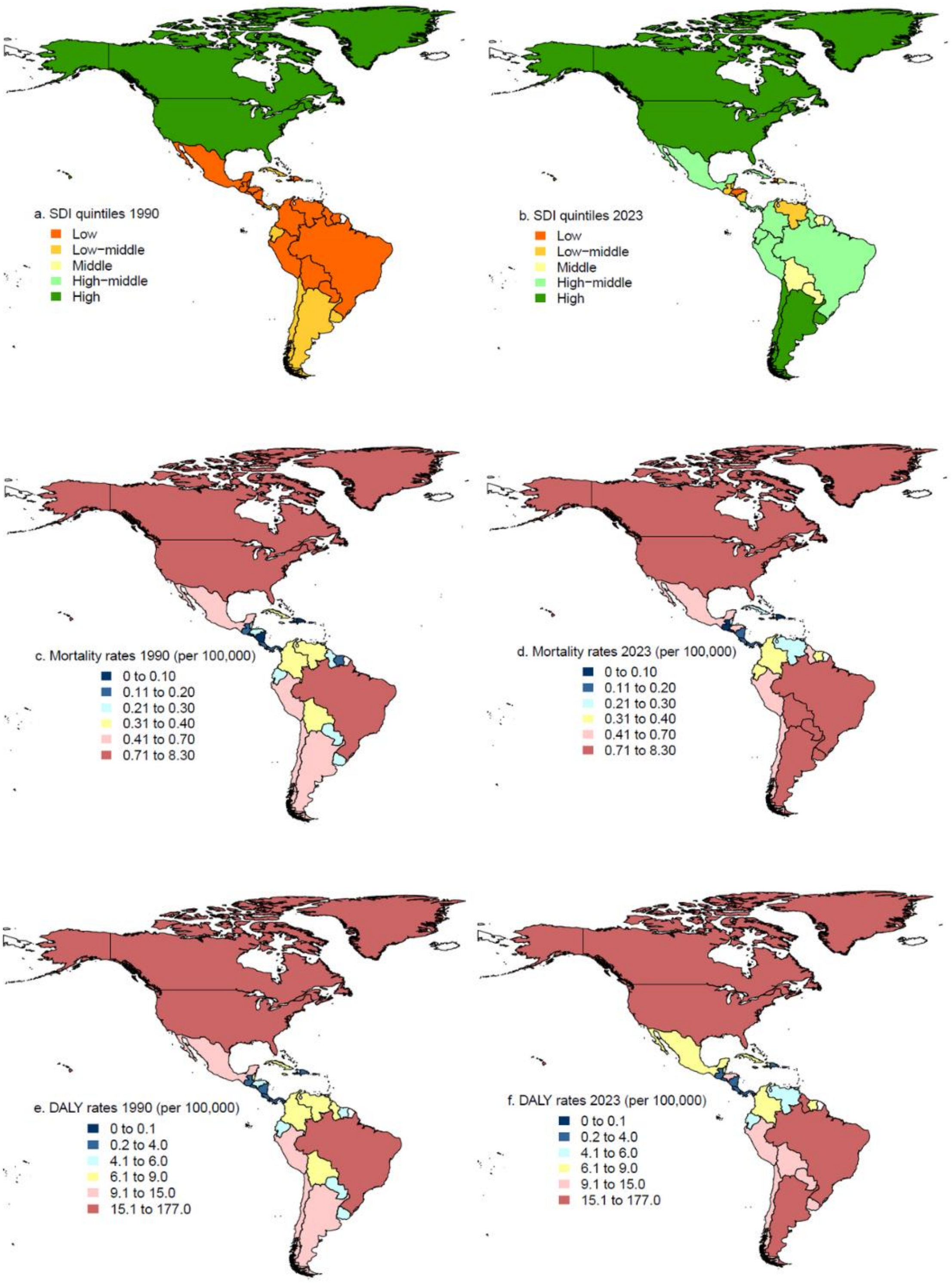

Figure S3. Socio-demographic index (SDI) quintiles (a,b) and ranges in age-standardised (c,d) mortality and (e,f) disability-adjusted life years (DALY) rates for cancer in women attributable to occupational asbestos exposure by countries in the Americas, 1990 and 2023.

SDI quintiles are based on GBD 2023 cutoffs. Low SDI: 0 to 53.16; Low-middle SDI: 53.16 to 63.05; Middle SDI: 63.05 to 67.67; High-middle SDI: 67.67 to 71.88; and High SDI: 71.88 to 100.

Mortality: age-standardized mortality rates per 100,000; DALY: age-standardized DALY rates per 100,000; rates are presented in ranges.

Cancer in women: laryngeal, lung and ovarian cancers and mesothelioma.

Elaborated by the authors (2025).

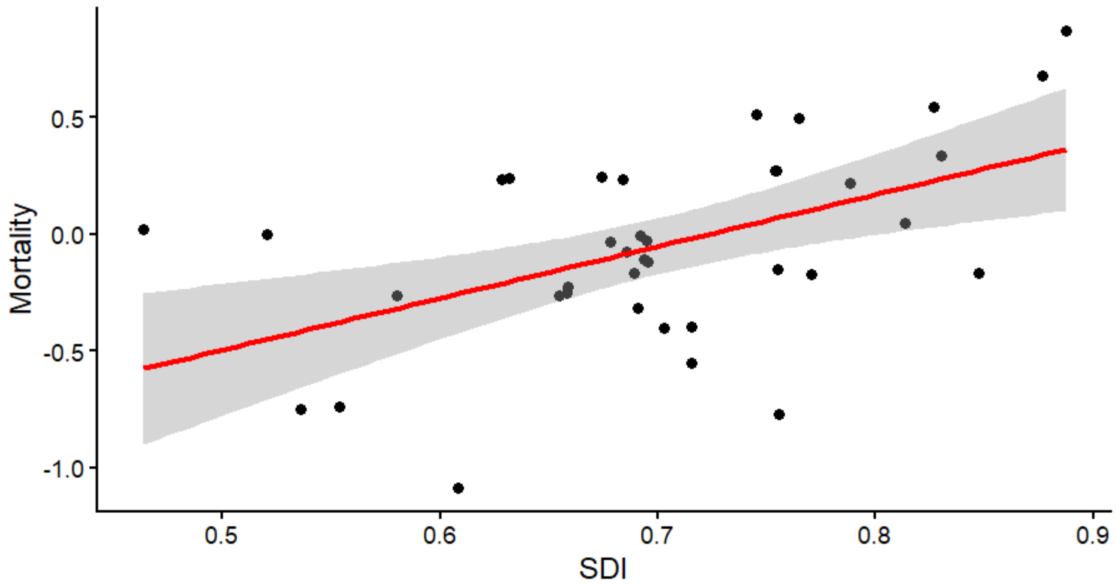

Figure S4. Linear regression for country-level Socio-demographic index (SDI) values and age-standardised mortality and disability-adjusted life years (DALY) rates for cancer in both sexes attributable to occupational asbestos exposure in the Americas, 2023.

Mortality: age-standardized mortality rates per 100,000; DALY: age-standardized DALY rates per 100,000.

Cancer: laryngeal, lung and ovarian cancers and mesothelioma.

Elaborated by the authors (2025).

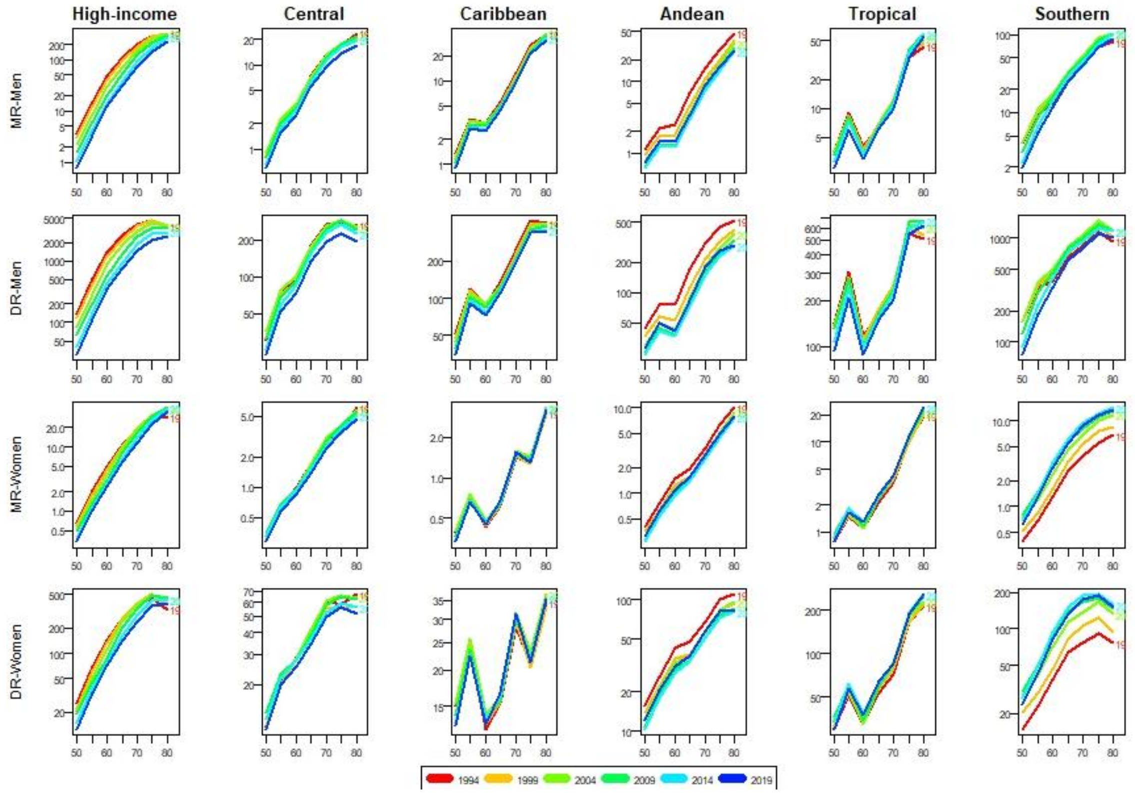

Figure S5. Mortality and disability-adjusted life years (DALY) age-specific groups rates for cancer in men and women attributable to occupational asbestos exposure, within each period by age-groups in the regions of the Americas, 1994-2023.

High-income: High-income North America; Central: Central Latin America; Andean: Andean Latin America; Tropical: Tropical Latin America; Southern: Southern Latin America.

MR: age-specific groups mortality rates per 100,000; DR: age-specific groups DALY rates per 100,000.

Cancer in men: laryngeal and lung cancers and mesothelioma.

Cancer in women: ovarian and lung cancers and mesothelioma.

Elaborated by the authors (2025).

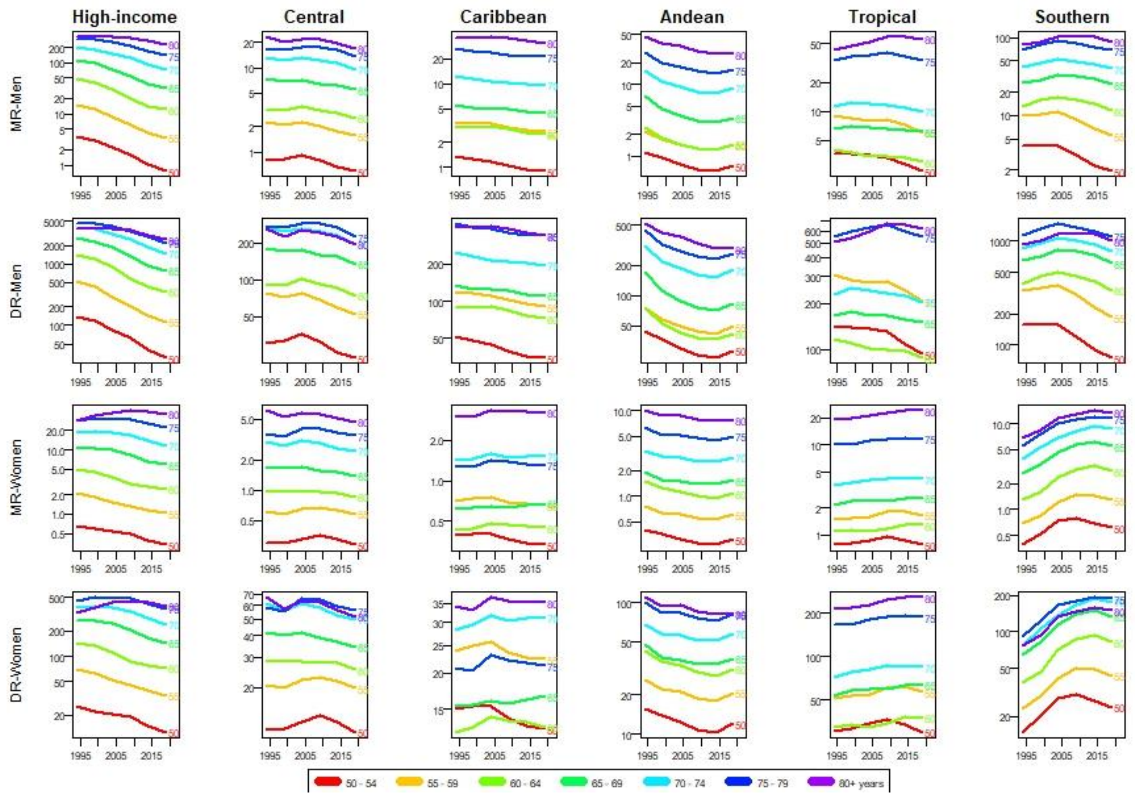

Figure S6. Mortality and disability-adjusted life years (DALY) age-specific groups rates for cancer in men and women attributable to occupational asbestos exposure, within each age-group by period in the regions of the Americas, 1994-2023.

High-income: High-income North America; Central: Central Latin America; Andean: Andean Latin America; Tropical: Tropical Latin America; Southern: Southern Latin America.

MR: age-specific groups mortality rates per 100,000; DR: age-specific groups DALY rates per 100,000.

Cancer in men: laryngeal and lung cancers and mesothelioma.

Cancer in women: ovarian and lung cancers and mesothelioma.

Elaborated by the authors (2025).

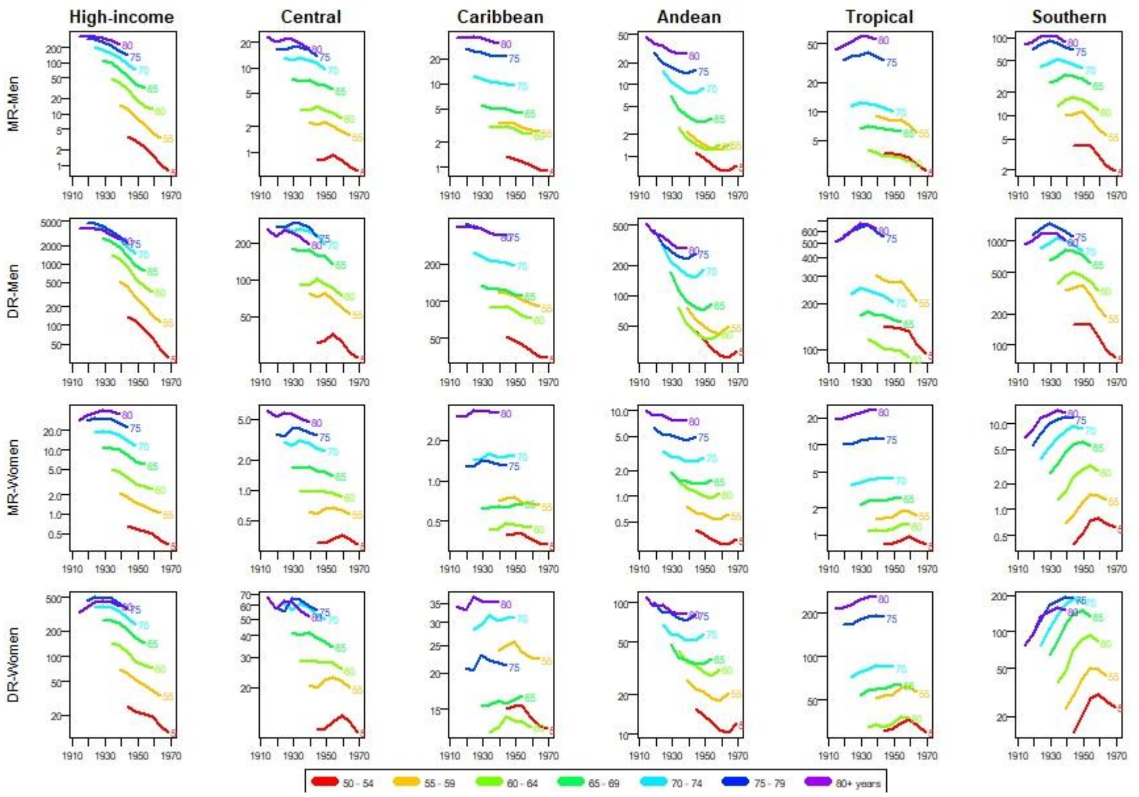

Figure S7. Mortality and disability-adjusted life years (DALY) age-specific groups rates for cancer in men and women attributable to occupational asbestos exposure, within each age-group by birth cohorts in the regions of the Americas, 1994-2023.

High-income: High-income North America; Central: Central Latin America; Andean: Andean Latin America; Tropical: Tropical Latin America; Southern: Southern Latin America.

MR: age-specific groups mortality rates per 100,000; DR: age-specific groups DALY rates per 100,000.

Cancer in men: laryngeal and lung cancers and mesothelioma.

Cancer in women: ovarian and lung cancers and mesothelioma.

Elaborated by the authors (2025).

HIGH-INCOME NORTH AMERICA

Canada

Ban on the use of asbestos **2018**

1990	Number (95%UI)	Rate (95%UI)
Deaths	**4,182.2** (3,184.2;5,182.0)	**12.3** (9.4;15.3)
DALYs	**83,969.9** (62,688.0;105,726.1)	**249.1** (184.9;313.8)

2023	Number (95%UI)	Rate (95%UI)
Deaths	**6,428.2** (4,987.3;7,967.5)	**7.4** (5.8;9.1)
DALYs	**105,036.1** (80,996.0;131,597.2)	**125.3** (96.5;157.7)

Greenland

Ban on the use of asbestos **2010**

1990	Number (95%UI)	Rate (95%UI)
Deaths	**1.9** (1.1;3.2)	**7.4** (4.4;12.9)
DALYs	**40.2** (22.7;68.3)	**143.5** (81,6;242.7)

2023	Number (95%UI)	Rate (95%UI)
Deaths	**12.1** (8.2;16.6)	**22.2** (15.1;30.3)
DALYs	**225.8** (147.4;318.9)	**371.8** (255.0;513.9)

USA

Ban of some processes using asbestos **1973-1978**

Asbestos Ban and Phase-Out Rule **1989**

Asbestos Ban and Phase-Out Rule is overturned **1991**

Effective phase-out of all types of asbestos **2024**

1990	Number (95%UI)	Rate (95%UI)
Deaths	**32,856.2** (24,791.3;41,262.1)	**9.6** (7.2;12.1)
DALYs	**637,609.5** (475,201.3;812,693.1)	**190.6** (141.4;243.5)

2023	Number (95%UI)	Rate (95%UI)
Deaths	**31,204.8** (24,130.8;39,951.1)	**4.7** (3.7;6.1)
DALYs	**518,415.2** (398,980.2;660,816.4)	**79.7** (61.3;101.7)

CENTRAL LATIN AMERICA

Colombia

Restrictions on the use of asbestos **2007 - 2009**

Ban on the use of asbestos **2019**

1990	Number (95%UI)	Rate (95%UI)
Deaths	**125.6** (92.4;162.0)	**0.8** (0.6;1.0)
DALYs	**2,865.5** (2,125.8;3,692.3)	**16.8** (12.4;21.7)

2023	Number (95%UI)	Rate (95%UI)
Deaths	**401.3** (285.8;564.1)	**0.7** (0.5;1.0)
DALYs	**8,228.5** (5,817.9;11,489.6)	**13.9** (9.8;19.3)

Costa Rica

1990	Number (95%UI)	Rate (95%UI)
Deaths	**9.3** (6.3;13.2)	**0.6** (0.4;0.9)
DALYs	**159.7** (110.1;225.8)	**9.8** (6.8;13.9)

2023	Number (95%UI)	Rate (95%UI)
Deaths	**23.5** (15.7;33.3)	**0.4** (0.3;0.6)
DALYs	**406.3** (275.1;579.7)	**6.8** (4.6;9.7)

El Salvador

1990	Number (95%UI)	Rate (95%UI)
Deaths	**0.7** (0.4;1.1)	**0.03** (0.01;0.04)
DALYs	**14.7** (7.3;24.0)	**0.5** (0.3;0.8)

2023	Number (95%UI)	Rate (95%UI)
Deaths	**5.0** (3.1;7.6)	**0.08** (0.05;0.13)
DALYs	**96.9** (59.7;155.2)	**1.6** (1.0;2.5)

Guatemala

Ban on the use of asbestos **2019**

1990	Number (95%UI)	Rate (95%UI)
Deaths	**6.4** (4.8;8.4)	**0.2** (0.2;0.3)
DALYs	**145.0** (107.8;184.4)	**4.4** (3.3;5.7)

2023	Number (95%UI)	Rate (95%UI)
Deaths	**18.2** (13.1;25.7)	**0.2** (01;0.3)
DALYs	**378.4** (265.5;524.5)	**3.4** (2.4;4.7)

Honduras

Ban on the use of asbestos, with some exceptions **2004**

1990	Number (95%UI)	Rate (95%UI)
Deaths	**8.9** (5.0;13.7)	**0.5** (0.3;0.8)
DALYs	**195.3** (110.1;293.0)	**10.2** (5.7;15.5)

2023	Number (95%UI)	Rate (95%UI)
Deaths	**61.7** (40.0;89.2)	**1.0** (0.6;1.5)
DALYs	**1,161.0** (790.0;1,656.8)	**17.4** (11.6;24.8)

Mexico

1990	Number (95%UI)	Rate (95%UI)
Deaths	**497.8** (369.9;646.2)	**1.3** (0.9;1.7)
DALYs	**10,620.9** (7,995.9;13,639.0)	**25.9** (19.3;33.4)

2023	Number (95%UI)	Rate (95%UI)
Deaths	**1,036.1** (820.3;1,302.9)	**0.8** (0.6;1.0)
DALYs	**21,714.8** (17,334.2;27,017.0)	**15.8** (12.6;19.8)

Nicaragua

Year	Measure	Number (95%UI)	Rate (95%UI)
1990	Deaths	**1.8** (1.2;2.6)	**0.1** (0.1;0.2)
1990	DALYs	**39.6** (27.3;55.3)	**2.7** (1.8;3.8)
2023	Deaths	**8.3** (6.0;11.4)	**0.2** (0.1;0.2)
2023	DALYs	**189.5** (136.0;264.2)	**3.8** (2.7;5.3)

Panama

Year	Measure	Number (95%UI)	Rate (95%UI)
1990	Deaths	**5.5** (3.9;7.9)	**0.4** (0.3;0.6)
1990	DALYs	**100.4** (73.0;142.5)	**7.1** (5.1;10.1)
2023	Deaths	**12.9** (8.8;18.3)	**0.3** (0.2;0.4)
2023	DALYs	**224.7** (155.2;322.6)	**4.9** (3.4;7.0)

Venezuela

Year	Measure	Number (95%UI)	Rate (95%UI)
1990	Deaths	**52.7** (38.0;70.5)	**0.6** (0.5;0.9)
1990	DALYs	**1,213.6** (880.9;1,630.7)	**13.5** (9.7;18.0)
2023	Deaths	**161.9** (104.9;247.1)	**0.5** (0.4;0.8)
2023	DALYs	**3,449.5** (2,215.3;5,220.5)	**10.9** (7.1;16.6)

CARIBBEAN

Cuba

Year	Measure	Number (95%UI)	Rate (95%UI)
1990	Deaths	**134.7** (88.9;189.7)	**1.3** (0.8;1.8)
1990	DALYs	**2,545.1** (1,749.8;3,516.7)	**24.1** (16.7;33.3)
2023	Deaths	**197.6** (130.4;299.1)	**0.9** (0.6;1.4)
2023	DALYs	**3,721.0** (2,475.8;5,551.1)	**18.0** (11.9;27.0)

Dominican Republic

Year	Measure	Number (95%UI)	Rate (95%UI)
1990	Deaths	**11.7** (7.2; 18.4)	**0.3** (0.2;0.5)
1990	DALYs	**228.4** (144.3;354.5)	**6.2** (3.9;9.8)
2023	Deaths	**51.3** (31.0;85.4)	**0.6** (0.3;0.9)
2023	DALYs	**939.6** (558.9;1505.8)	**9.8** (5.8;15.9)

Haiti

Year	Measure	Number (95%UI)	Rate (95%UI)
1990	Deaths	**20.9** (12.5;35.8)	**0.7** (0.4;1.3)
1990	DALYs	**501.9** (312.2;856.3)	**15.4** (9.4;27.0)
2023	Deaths	**66.3** (41.4;108.5)	**1.1** (0.6;1.8)
2023	DALYs	**1,642.7** (1,049.2;2,581.6)	**22.0** (13.8;35.8)

Puerto Rico

Year	Measure	Number (95%UI)	Rate (95%UI)
1990	Deaths	**51.0** (36.1;71.7)	**1.3** (0.9;1.9)
1990	DALYs	**1,000.7** (707.1;1,379.9)	**26.7** (18.8;36.8)
2023	Deaths	**65.0** (43.3;95.3)	**0.7** (0.5;1.0)
2023	DALYs	**1,086.1** (719.4;1,586.6)	**13.0** (8.9;18.6)

ANDEAN LATIN AMERICA

Bolivia

Year	Measure	Number (95%UI)	Rate (95%UI)
1990	Deaths	**23.9** (15.0;38.8)	**0.9** (0.6;1.5)
1990	DALYs	**493.6** (321.5;777.0)	**16.7** (10.5;26.6)
2023	Deaths	**149.8** (95.9;230.0)	**1.7** (1.1;2.7)
2023	DALYs	**3,018.5** (1,936.9;4,613.5)	**32.7** (21.2;49.8)

Ecuador

Year	Measure	Number (95%UI)	Rate (95%UI)
1990	Deaths	**20.8** (15.6;27.0)	**0.5** (0.3;0.6)
1990	DALYs	**396.7** (306.0;512.0)	**8.1** (6.2;10.4)
2023	Deaths	**83.4** (62.1;111.2)	**0.5** (0.4;0.6)
2023	DALYs	**1,406.0** (1,053.6;1,842.4)	**8.0** (6.0;10.5)

Peru

2014: Ban on the import of crocidolite asbestos

Year	Measure	Number (95%UI)	Rate (95%UI)
1990	Deaths	**132.8** (84.5;188.7)	**1.3** (0.8;1.8)
1990	DALYs	**2,569.0** (1,695.3;3,625.6)	**23.0** (14.8;32.4)
2023	Deaths	**300.3** (201.8;440.2)	**0.8** (0.6;1.2)
2023	DALYs	**5,420.9** (3,635.3;7,777.6)	**14.8** (10.0;21.2)

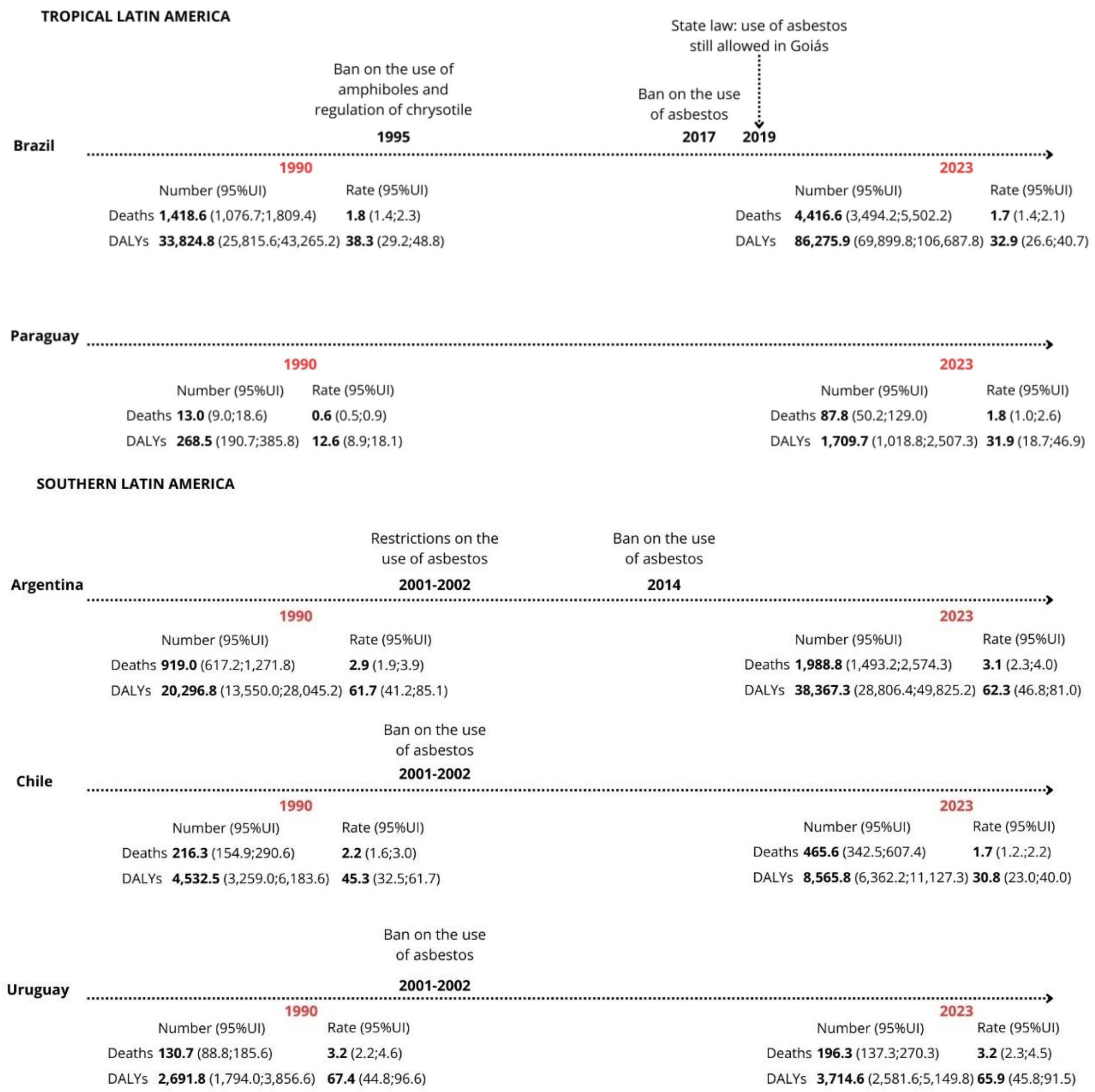

Figure S8. Country-level asbestos policy timelines and mortality and disability-adjusted life years (DALYs) numbers and rates for cancer in both sexes attributable to occupational asbestos exposure in 1990 and 2023 in the regions of the Americas.

Caribbean: only the countries with the highest numbers and rates are displayed.

Number: deaths and DALYs for all ages; Rates: mortality and DALY age-standardized rates per 100,000.

Cancer in both sexes attributable to occupational asbestos exposure: laryngeal, lung and ovarian cancers and mesothelioma.

Elaborated by the authors (2025).

	Mortality - 2023						DALY - 2023					
	**Number**	95%UI		**Rate**	95%UI		**Number**	95%UI		**Rate**	95%UI	
**High-income North America**												
**Both sexes**												
All cancers	**37,645·7**	29,317·1	47,781·1	**5·1**	3·9	6·4	**623,686·3**	481,846·9	796,787·6	**84·9**	65·6	108·5
Larynx	**471·1**	265·4	695·5	**0·1**	0·0	0·1	**8,319·0**	4,735·7	12,454·5	**1·1**	0·6	1·7
Lung	**33,208·6**	25,075·1	43,489·4	**4·5**	3·4	5·8	**547,143·6**	404,178·9	719,723·7	**74·2**	54·6	97·7
Ovary	**919·0**	434·9	1,441·1	**0·1**	0·1	0·2	**15,259·2**	7,222·0	23,833·8	**2·1**	1·0	3·3
Mesothelioma	**3,047·0**	2,683·1	3,419·9	**0·4**	0·4	0·5	**52,964·5**	47,434·8	59,059·8	**7·6**	6·8	8·4
**Male**												
All cancers	**30,778·0**	23,156·1	40,472·2	**9·5**	7·1	12·4	**511,254·7**	378,852·1	681,121·8	**154·4**	114·8	205·8
Larynx	**447·0**	243·9	664·4	**0·1**	0·1	0·2	**7,873·3**	4,276·5	11,884·5	**2·4**	1·3	3·6
Lung	**28,031·8**	20,390·9	37,723·2	**8·6**	6·2	11·6	**464,344·8**	330,946·7	630,758·9	**139·9**	99·9	190·3
Mesothelioma	**2,299·2**	2,005·9	2,630·9	**0·7**	0·6	0·8	**39,036·6**	34,211·7	44,311·2	**12·2**	10·6	13·8
**Female**												
All cancers	**6,867·7**	4,827·7	8,936·6	**1·7**	1·2	2·2	**112,431·6**	81,436·3	144,510·8	**28·4**	20·8	36·2
Larynx	**24·1**	12·4	38·3	**0·0**	0·0	0·0	**445·8**	225·5	718·4	**0·1**	0·1	0·2
Lung	**5,176·8**	3,260·6	7,051·6	**1·2**	0·8	1·7	**82,798·8**	52,788·9	112,541·1	**20·5**	13·2	28·0
Ovary	**919·0**	434·9	1,441·1	**0·2**	0·1	0·3	**15,259·2**	7,222·0	23,833·8	**3·9**	1·8	6·0
Mesothelioma	**747·9**	595·5	893·0	**0·2**	0·2	0·2	**13,927·9**	11,484·5	16,598·7	**3·8**	3·2	4·6
**Central Latin America**												
**Both sexes**												
All cancers	**1,728·8**	1,323·3	2,275·5	**0·7**	0·5	0·9	**35,849·6**	27,873·4	46,380·7	**13·4**	10·4	17·3
Larynx	**42·3**	21·8	65·2	**0·0**	0·0	0·0	**817·9**	415·2	1,285·2	**0·3**	0·2	0·5
Lung	**1,126·7**	746·0	1,624·9	**0·4**	0·3	0·6	**21,341·4**	13,997·1	31,173·7	**8·1**	5·3	11·8
Ovary	**111·5**	52·2	183·2	**0·0**	0·0	0·1	**2,477·6**	1,147·6	4,066·2	**0·9**	0·4	1·5
Mesothelioma	**448·3**	401·5	500·3	**0·2**	0·1	0·2	**11,212·7**	9,970·3	12,591·5	**4·1**	3·6	4·6
**Male**												
All cancers	**1,257·5**	889·7	1,742·8	**1·1**	0·8	1·5	**25,821·1**	18,509·3	35,415·0	**21·2**	15·1	29·2
Larynx	**39·2**	19·6	61·8	**0·0**	0·0	0·1	**763·9**	376·3	1,218·1	**0·6**	0·3	1·0
Lung	**919·8**	559·2	1,408·5	**0·8**	0·5	1·2	**17,584·1**	10,675·4	27,002·9	**14·7**	8·9	22·6
Mesothelioma	**298·4**	259·9	343·3	**0·2**	0·2	0·3	**7,473·1**	6,409·7	8,681·4	**5·9**	5·1	6·8
**Female**												
All cancers	**471·3**	367·9	599·2	**0·3**	0·3	0·4	**10,028·5**	7,868·0	12,791·7	**6·9**	5·4	8·7
Larynx	**3·1**	1·5	5·2	**0·0**	0·0	0·0	**53·9**	25·5	92·2	**0·0**	0·0	0·1
Lung	**206·9**	132·7	299·2	**0·1**	0·1	0·2	**3,757·4**	2,440·0	5,382·1	**2·6**	1·7	3·7
Ovary	**111·5**	52·2	183·2	**0·1**	0·0	0·1	**2,477·6**	1,147·6	4,066·2	**1·7**	0·8	2·8
Mesothelioma	**149·8**	123·6	178·0	**0·1**	0·1	0·1	**3,739·6**	3,096·4	4,494·8	**2·5**	2·1	3·0
**Caribbean**												
**Both sexes**												
All cancers	**478·6**	338·5	686·0	**0·8**	0·6	1·2	**9,459·6**	6,725·9	13,272·1	**16·7**	11·9	23·4
Larynx	**20·7**	10·3	34·3	**0·0**	0·0	0·1	**423·0**	206·0	705·8	**0·7**	0·4	1·2
Lung	**378·4**	241·7	582·4	**0·7**	0·4	1·0	**6,936·4**	4,349·1	10,700·5	**12·2**	7·6	18·8
Ovary	**11·2**	4·8	18·4	**0·0**	0·0	0·0	**270·8**	110·1	460·6	**0·5**	0·2	0·8

Mesothelioma	**68·2**	57·2	80·9	**0·1**	0·1	0·1	**1,829·3**	1,497·3	2,198·0	**3·3**	2·7	3·9
**Male**												
All cancers	**408·3**	271·2	597·8	**1·6**	1·1	2·4	**7,829·2**	5,269·1	11,391·0	**30·0**	20·2	43·6
Larynx	**19·9**	9·6	33·5	**0·1**	0·0	0·1	**406·2**	192·2	694·7	**1·5**	0·7	2·6
Lung	**339·7**	205·2	533·0	**1·4**	0·8	2·1	**6,185·5**	3,686·2	9,787·5	**23·8**	14·3	37·7
Mesothelioma	**48·7**	40·1	58·1	**0·2**	0·2	0·2	**1,237·4**	992·2	1,512·9	**4·6**	3·7	5·7
**Female**												
All cancers	**70·3**	51·1	96·1	**0·2**	0·2	0·3	**1,630·4**	1,199·5	2,236·0	**5·5**	4·0	7·5
Larynx	**0·8**	0·4	1·4	**0·0**	0·0	0·0	**16·8**	7·8	29·1	**0·1**	0·0	0·1
Lung	**38·7**	23·9	58·0	**0·1**	0·1	0·2	**750·9**	460·1	1,138·4	**2·5**	1·5	3·7
Ovary	**11·2**	4·8	18·4	**0·0**	0·0	0·1	**270·8**	110·1	460·6	**0·9**	0·4	1·6
Mesothelioma	**19·5**	14·9	25·8	**0·1**	0·1	0·1	**592·0**	431·9	817·7	**2·1**	1·5	2·9
**Andean Latin America**												
**Both sexes**												
All cancers	**533·5**	377·3	729·0	**0·9**	0·6	1·2	**9,845·3**	6,994·4	13,364·5	**15·4**	11·0	20·9
Larynx	**10·9**	5·1	18·0	**0·0**	0·0	0·0	**183·1**	85·3	305·3	**0·3**	0·1	0·5
Lung	**367·6**	228·8	553·4	**0·6**	0·4	0·9	**6,144·4**	3,754·1	9,373·6	**9·8**	6·0	14·9
Ovary	**29·3**	12·6	55·7	**0·0**	0·0	0·1	**578·5**	246·9	1,097·4	**0·9**	0·4	1·7
Mesothelioma	**125·7**	93·5	166·0	**0·2**	0·1	0·3	**2,939·3**	2,147·0	3,916·8	**4·5**	3·3	6·0
**Male**												
All cancers	**369·2**	239·3	536·8	**1·3**	0·9	1·9	**6,686·5**	4,384·9	9,766·3	**22·8**	14·8	33·3
Larynx	**9·9**	4·3	16·8	**0·0**	0·0	0·1	**165·3**	69·5	281·3	**0·6**	0·2	1·0
Lung	**280·3**	155·2	435·0	**1·0**	0·6	1·6	**4,681·8**	2,498·8	7,511·6	**16·3**	8·8	25·8
Mesothelioma	**79·0**	52·4	105·9	**0·3**	0·2	0·4	**1,839·4**	1,216·9	2,476·0	**5·9**	3·9	8·0
**Female**												
All cancers	**164·3**	108·6	244·2	**0·5**	0·3	0·7	**3,158·8**	2,078·1	4,606·6	**9·2**	6·1	13·5
Larynx	**1·0**	0·4	2·2	**0·0**	0·0	0·0	**17·9**	7·1	37·7	**0·1**	0·0	0·1
Lung	**87·3**	47·4	149·7	**0·3**	0·1	0·4	**1,462·6**	728·7	2,478·3	**4·3**	2·1	7·3
Ovary	**29·3**	12·6	55·7	**0·1**	0·0	0·2	**578·5**	246·9	1,097·4	**1·7**	0·7	3·2
Mesothelioma	**46·7**	28·0	72·9	**0·1**	0·1	0·2	**1,099·8**	639·4	1,718·0	**3·2**	1·9	5·0
**Tropical Latin America**												
**Both sexes**												
All cancers	**4,504·4**	3,548·1	5,617·6	**1·7**	1·4	2·2	**87,985·5**	71,073·3	109,031·7	**32·9**	26·6	40·8
Larynx	**164·7**	92·9	251·1	**0·1**	0·0	0·1	**3,511·1**	1,993·9	5,399·0	**1·3**	0·7	2·0
Lung	**3,293·1**	2,383·0	4,332·0	**1·3**	0·9	1·7	**58,662·5**	41,656·6	78,047·2	**22·1**	15·7	29·3
Ovary	**232·6**	109·7	367·6	**0·1**	0·0	0·1	**4,294·7**	2,041·2	6,795·6	**1·6**	0·8	2·5
Mesothelioma	**814·1**	728·0	911·5	**0·3**	0·3	0·3	**21,517·4**	19,201·4	24,238·6	**7·9**	7·1	9·0
**Male**												
All cancers	**2,910·4**	2,072·4	3,886·9	**2·6**	1·9	3·5	**58,761·3**	42,627·9	77,780·3	**49·8**	36·0	65·9
Larynx	**144·8**	74·9	228·1	**0·1**	0·1	0·2	**3,181·7**	1,672·4	5,024·9	**2·6**	1·4	4·2
Lung	**2,298·9**	1,494·0	3,254·3	**2·1**	1·4	3·0	**42,453·8**	27,104·7	61,639·8	**36·7**	23·6	52·8
Mesothelioma	**466·8**	409·5	534·6	**0·4**	0·3	0·4	**13,125·8**	11,307·4	14,999·1	**10·5**	9·0	12·0
**Female**												
All cancers	**1,594·0**	1,171·8	2,026·4	**1·1**	0·8	1·3	**29,224·3**	22,523·6	36,267·2	**19·6**	15·2	24·3
Larynx	**19·9**	9·7	31·9	**0·0**	0·0	0·0	**329·4**	161·9	523·1	**0·2**	0·1	0·3
Lung	**994·2**	643·2	1,374·7	**0·7**	0·4	0·9	**16,208·6**	10,409·0	22,286·5	**10·8**	6·9	14·8

Ovary	**232·6**	109·7	367·6	**0·2**	0·1	0·2	**4,294·7**	2,041·2	6,795·6	**2·9**	1·4	4·6
Mesothelioma	**347·3**	279·0	413·4	**0·2**	0·2	0·3	**8,391·6**	6,934·7	10,072·0	**5·8**	4·8	6·9
**Southern Latin America**												
**Both sexes**												
All cancers	**2,650·8**	2,016·3	3,430·8	**2·7**	2·1	3·5	**50,650·3**	38,471·6	66,101·0	**53·1**	40·4	69·3
Larynx	**56·5**	31·0	84·4	**0·1**	0·0	0·1	**1,097·7**	598·5	1,664·5	**1·1**	0·6	1·7
Lung	**2,160·9**	1,494·2	2,948·4	**2·2**	1·5	3·0	**39,841·7**	27,026·0	55,729·4	**41·3**	28·0	58·0
Ovary	**100·6**	47·4	167·6	**0·1**	0·0	0·2	**1,971·7**	928·5	3,280·6	**2·1**	1·0	3·4
Mesothelioma	**332·9**	302·0	367·4	**0·4**	0·3	0·4	**7,739·1**	7,020·3	8,546·8	**8·5**	7·7	9·4
**Male**												
All cancers	**2,072·9**	1,405·0	2,854·8	**5·1**	3·5	7·0	**39,435·8**	26,436·2	55,002·8	**95·2**	64·1	132·6
Larynx	**52·2**	25·9	80·2	**0·1**	0·1	0·2	**1,018·1**	507·7	1,589·9	**2·5**	1·2	3·8
Lung	**1,811·7**	1,158·5	2,590·8	**4·4**	2·9	6·3	**33,536·7**	20,798·5	49,097·1	**80·8**	50·3	118·0
Mesothelioma	**209·0**	185·2	239·6	**0·5**	0·5	0·6	**4,880·9**	4,274·9	5,621·8	**11·9**	10·4	13·7
**Female**												
All cancers	**577·9**	434·5	741·0	**1·0**	0·8	1·3	**11,214·5**	8,555·2	14,135·3	**21·2**	16·2	26·7
Larynx	**4·3**	2·1	7·1	**0·0**	0·0	0·0	**79·6**	38·6	130·1	**0·1**	0·1	0·2
Lung	**349·1**	220·8	493·6	**0·6**	0·4	0·9	**6,305·0**	3,992·2	8,935·2	**11·5**	7·3	16·5
Ovary	**100·6**	47·4	167·6	**0·2**	0·1	0·3	**1,971·7**	928·5	3,280·6	**3·7**	1·7	6·2
Mesothelioma	**123·9**	104·6	144·8	**0·2**	0·2	0·3	**2,858·2**	2,427·0	3,368·4	**5·8**	4·9	6·8

Table S1. Mortality and disability-adjusted life years (DALYs) numbers and rates for cancer attributable to occupational asbestos exposure, by sex and regions, in 2023 in the Americas.

Number: number of deaths and DALYs for all ages; Rate: age-standardized rates per 100,000; 95%UI: 95% uncertainty interval; All cancers attributable to occupational asbestos exposure: laryngeal, lung (trachea, bronchi and lungs) and ovarian cancers and mesothelioma.

Elaborated by the authors (2025).

Models	Laryngeal cancer			Lung cancer			Mesothelioma			Ovarian cancer		
**High-income North America**												
**Mortality**	**df**	**dev**	**p**	**df**	**dev**	**p**	**df**	**dev**	**p**	**df**	**dev**	**p**
**Men**										-	-	-
Age	35	987·6	NA	35	95328·0	NA	35	4284·0	NA	-	-	-
Age-drift[1]	34	252·1	<0·01	34	21562·0	<0·01	34	2067·0	<0·01	-	-	-
Age-cohort[2]	24	74·1	<0·01	24	1165·0	<0·01	24	99·0	<0·01	-	-	-
Age-period-cohort[3]	20	10·8	<0·01	20	426·0	<0·01	20	80·0	<0·01	-	-	-
Age-period[4]	30	242·0	<0·01	30	19254·0	<0·01	30	1687·0	<0·01	-	-	-
Age-drift[5]	34	252·1	<0·05	34	21562·0	<0·01	34	2067·0	<0·01	-	-	-
**Women**												
Age	-	-	-	35	4190·1	NA	35	153·7	NA	35	622·2	NA
Age-drift[1]	-	-	-	34	3723·8	<0·01	34	149·3	<0·05	34	311·6	<0·01
Age-cohort[2]	-	-	-	24	72·1	<0·01	24	24·5	<0·01	24	12·1	<0·01
Age-period-cohort[3]	-	-	-	20	58·1	<0·01	20	10·9	<0·01	20	11·8	=0·99
Age-period[4]	-	-	-	30	2937·9	<0·01	30	143·6	<0·01	30	222·1	<0·01
Age-drift[5]	-	-	-	34	3723·8	<0·01	34	149·3	=0·22	34	311·6	<0·01
**DALY**	**df**	**dev**	**p**	**df**	**dev**	**p**	**df**	**dev**	**p**	**df**	**dev**	**p**
**Men**										-	-	-
Age	35	23956·0	NA	35	2145989·0	NA	35	109346·0	NA	-	-	-
Age-drift[1]	34	4542·0	<0·01	34	363978·0	<0·01	34	37183·0	<0·01	-	-	-
Age-cohort[2]	24	1390·0	<0·01	24	22239·0	<0·01	24	2256·0	<0·01	-	-	-
Age-period-cohort[3]	20	194·0	<0·01	20	7905·0	<0·01	20	1292·0	<0·01	-	-	-
Age-period[4]	30	4168·0	<0·01	30	326278·0	<0·01	30	29242·0	<0·01	-	-	-
Age-drift[5]	34	4542·0	<0·01	34	363978·0	<0·01	34	37183·0	<0·01	-	-	-
**Women**												
Age	-	-	-	35	84098·0	NA	35	3038·0	NA	35	13647·0	NA
Age-drift[1]	-	-	-	34	56477·0	<0·01	34	2186·0	<0·01	34	4481·0	<0·01
Age-cohort[2]	-	-	-	24	784·0	<0·01	24	335·0	<0·01	24	227·0	<0·01
Age-period-cohort[3]	-	-	-	20	726·0	<0·01	20	177·0	<0·01	20	181·0	<0·01
Age-period[4]	-	-	-	30	45969·0	<0·01	30	2111·0	<0·01	30	3343·0	<0·01
Age-drift[5]	-	-	-	34	56477·0	<0·01	34	2186·0	<0·01	34	4481·0	<0·01
**Central Latin America**												
**Models**	**Laryngeal cancer**			**Lung cancer**			**Mesothelioma**			**Ovarian cancer**		
**Mortality**	**df**	**dev**	**p**	**df**	**dev**	**p**	**df**	**dev**	**p**	**df**	**dev**	**p**
**Men**												
Age	35	31·8	NA	35	437·6	NA	35	68·6	NA	-	-	-
Age-drift[1]	34	3·7	<0·01	34	105·5	<0·01	34	53·5	<0·01	-	-	-
Age-cohort[2]	24	1·8	=0·99	24	42·2	<0·01	24	28·2	<0·01	-	-	-
Age-period-cohort[3]	20	0·4	=0·85	20	9·5	<0·01	20	6·4	<0·01	-	-	-
Age-period[4]	30	1·3	=0·99	30	38·8	<0·01	30	19·6	=0·21	-	-	-
Age-drift[5]	34	3·7	=0·66	34	105·5	<0·01	34	53·5	<0·01	-	-	-
**Women**										-	-	-
Age	-	-	-	35	58·8	NA	35	18·8	NA	35	12·5	NA
Age-drift[1]	-	-	-	34	12·1	<0·01	34	17·5	=0·25	34	7·1	<0·05
Age-cohort[2]	-	-	-	24	5·8	=0·79	24	12·5	=0·89	24	5·1	=1·00
Age-period-cohort[3]	-	-	-	20	**3·2**	=0·61	20	**2·0**	<0·05	20	**1·3**	=0·44
Age-period[4]	-	-	-	30	6·7	=0·97	30	3·1	=1·00	30	3·9	=0·99
Age-drift[5]	-	-	-	34	12·1	=0·25	34	17·5	<0·05	34	7·1	=0·53
**DALY**	**df**	**dev**	**p**	**df**	**dev**	**p**	**df**	**dev**	**p**	**df**	**dev**	**p**
**Men**												
Age	35	677·5	NA	35	9758·0	NA	35	1751·0	NA	-	-	-
Age-drift[1]	34	80·6	<0·01	34	2169·0	<0·01	34	1518·0	<0·01	-	-	-
Age-cohort[2]	24	35·0	<0·01	24	831·0	<0·01	24	680·0	<0·01	-	-	-
Age-period-cohort[3]	20	11·0	<0·01	20	226·0	<0·01	20	155·0	<0·01	-	-	-
Age-period[4]	30	34·5	<0·01	30	927·0	<0·01	30	568·0	<0·01	-	-	-
Age-drift[5]	34	80·6	<0·01	34	2169·0	<0·01	34	1518·0	<0·01	-	-	-
**Women**										-	-	-
Age	-	-	-	35	1112·6	NA	35	469·2	NA	35	329·0	NA
Age-drift[1]	-	-	-	34	217·9	<0·01	34	427·2	<0·01	34	146·3	<0·01
Age-cohort[2]	-	-	-	24	113·0	<0·01	24	305·3	<0·01	24	105·0	<0·01
Age-period-cohort[3]	-	-	-	20	58·2	<0·01	20	47·6	<0·01	20	25·0	<0·01
Age-period[4]	-	-	-	30	131·5	<0·01	30	75·2	<0·01	30	87·3	<0·01
Age-drift[5]	-	-	-	34	217·9	<0·01	34	427·2	<0·01	34	146·3	<0·01
**Caribbean**												
**Models**	**Laryngeal cancer**			**Lung cancer**			**Mesothelioma**			**Ovarian cancer**		
**Mortality**	**df**	**dev**	**p**	**df**	**dev**	**p**	**df**	**dev**	**p**	**df**	**dev**	**p**
**Men**										-	-	-

Age	35	1·4	NA	35	65·7	NA	35	1·6	NA	-	-	-
Age-drift[1]	34	0·9	=0·47	34	13·5	<0·01	34	0·3	=0·26	-	-	-
Age-cohort[2]	24	0·2	=1·00	24	2·6	=0·36	24	0·2	=1·00	-	-	-
Age-period-cohort[3]	20	0·1	=1·00	20	1·8	=0·94	20	0·1	=1·00	-	-	-
Age-period[4]	30	0·7	=1·00	30	11·5	=0·47	30	0·3	=1·00	-	-	-
Age-drift[5]	34	0·9	=1·00	34	13·5	=0·71	34	0·3	=1·00	-	-	-
**Women**												
Age	-	-	-	35	5·0	NA	35	0·8	NA	35	0·3	NA
Age-drift[1]	-	-	-	34	4·5	=0·50	34	0·2	=0·47	34	0·3	=0·95
Age-cohort[2]	-	-	-	24	1·3	=0·97	24	0·2	=1·00	24	0·2	=1·00
Age-period-cohort[3]	-	-	-	20	0·4	=0·93	20	0·06	=1·00	20	0·04	=1·00
Age-period[4]	-	-	-	30	2·8	=0·99	30	0·1	=1·00	30	0·1	=1·00
Age-drift[5]	-	-	-	34	4·5	=0·79	34	0·2	=1·00	34	0·3	=1·00
**DALY**	**df**	**dev**	**p**	**df**	**dev**	**p**	**df**	**dev**	**p**	**df**	**dev**	**p**
**Men**										-	-	-
Age	35	23·3	NA	35	1514·2	NA	35	35·6	NA	-	-	-
Age-drift[1]	34	19·3	<0·05	34	277·8	<0·01	34	5·5	<0·01	-	-	-
Age-cohort[2]	24	4·0	=0·12	24	50·5	<0·01	24	3·8	=0·99	-	-	-
Age-period-cohort[3]	20	1·8	=0·70	20	39·6	<0·05	20	2·8	=0·90	-	-	-
Age-period[4]	30	15·9	=0·17	30	253·1	<0·01	30	4·4	=0·99	-	-	-
Age-drift[5]	34	19·3	=0·48	34	277·8	<0·01	34	5·5	=0·91	-	-	-
**Women**												
Age	-	-	-	35	123·8	NA	35	20·5	NA	35	7·7	NA
Age-drift[1]	-	-	-	34	123·4	=0·53	34	5·6	<0·01	34	7·1	=0·44
Age-cohort[2]	-	-	-	24	28·0	<0·01	24	5·1	=0·99	24	4·1	=0·98
Age-period-cohort[3]	-	-	-	20	9·4	<0·01	20	1·4	=0·45	20	1·0	=0·53
Age-period[4]	-	-	-	30	83·5	<0·01	30	2·0	=0·99	30	3·5	=0·99
Age-drift[5]	-	-	-	34	123·4	<0·01	34	5·6	=0·47	34	7·1	=0·45
**Andean Latin America**												
**Models**	**Laryngeal cancer**			**Lung cancer**			**Mesothelioma**			**Ovarian cancer**		
**Mortality**	**df**	**dev**	**p**	**df**	**dev**	**p**	**df**	**dev**	**p**	**df**	**dev**	**p**
**Men**										-	-	-
Age	35	9·8	NA	35	320·7	NA	35	29·8	NA	-	-	-
Age-drift[1]	34	3·7	<0·05	34	79·1	<0·01	34	18·0	<0·01	-	-	-
Age-cohort[2]	24	2·4	=1·00	24	72·3	=0·74	24	13·2	=0·90	-	-	-
Age-period-cohort[3]	20	0·2	=0·68	20	7·0	<0·01	20	0·9	<0·05	-	-	-
Age-period[4]	30	0·4	=1·00	30	19·6	=0·25	30	1·8	=0·99	-	-	-
Age-drift[5]	34	3·7	=0·50	34	79·1	<0·01	34	18·0	<0·01	-	-	-
**Women**												
Age	-	-	-	35	24·3	NA	35	10·1	NA	35	1·8	NA
Age-drift[1]	-	-	-	34	8·4	<0·01	34	6·5	=0·06	34	1·0	=0·36
Age-cohort[2]	-	-	-	24	7·6	=0·99	24	5·6	=1·00	24	0·7	=1·00
Age-period-cohort[3]	-	-	-	20	0·8	=0·15	20	0·1	=0·24	20	0·3	=0·97
Age-period[4]	-	-	-	30	1·7	=0·99	30	0·2	=1·00	30	0·5	=1·00
Age-drift[5]	-	-	-	34	8·4	=0·15	34	6·5	=0·18	34	1·0	=0·97
**DALY**	**df**	**dev**	**p**	**df**	**dev**	**p**	**df**	**dev**	**p**	**df**	**dev**	**p**
**Men**										-	-	-
Age	35	168·8	NA	35	6141·0	NA	35	658·5	NA	-	-	-
Age-drift[1]	34	70·9	<0·01	34	1700·0	<0·01	34	407·6	<0·01	-	-	-
Age-cohort[2]	24	50·8	<0·05	24	1564·0	<0·01	24	329·9	<0·01	-	-	-
Age-period-cohort[3]	20	3·5	<0·01	20	140·0	<0·01	20	18·5	<0·01	-	-	-
Age-period[4]	30	7·2	=0·96	30	326·0	<0·01	30	32·2	=0·19	-	-	-
Age-drift[5]	34	70·9	<0·01	34	1700·0	<0·01	34	407·6	<0·01	-	-	-
**Women**												
Age	-	-	-	35	491·6	NA	35	241·2	NA	35	34·1	NA
Age-drift[1]	-	-	-	34	180·2	<0·01	34	152·7	<0·01	34	21·6	<0·05
Age-cohort[2]	-	-	-	24	169·1	=0·35	24	136·5	=0·07	24	18·0	=0·97
Age-period-cohort[3]	-	-	-	20	18·8	<0·01	20	2·6	<0·01	20	6·9	=0·49
Age-period[4]	-	-	-	30	34·5	=0·11	30	5·4	=0·98	30	10·1	=0·92
Age-drift[5]	-	-	-	34	180·2	<0·01	34	152·7	<0·01	34	21·6	=0·68
**Tropical Latin America**												
**Models**	**Laryngeal cancer**			**Lung cancer**			**Mesothelioma**			**Ovarian cancer**		
**Mortality**	**df**	**dev**	**p**	**df**	**dev**	**p**	**df**	**dev**	**p**	**df**	**dev**	**p**
**Men**										-	-	-
Age	35	21·3	NA	35	621·4	NA	35	36·6	NA	-	-	-
Age-drift[1]	34	20·2	=0·31	34	575·1	<0·01	34	34·7	=0·17	-	-	-
Age-cohort[2]	24	1·9	=0·05	24	17·2	<0·01	24	9·6	<0·01	-	-	-
Age-period-cohort[3]	20	0·6	=0·85	20	12·0	=0·27	20	3·2	=0·17	-	-	-

Age-period[4]	30	13·1	=0·25	30	478·8	<0·01	30	13·6	=0·41	-	-	-
Age-drift[5]	34	20·2	=0·13	34	575·1	<0·01	34	34·7	<0·01	-	-	-
**Women**												
Age	-	-	-	35	210·7	NA	35	12·4	NA	35	4·4	NA
Age-drift[1]	-	-	-	34	25·4	<0·01	34	11·6	=0·39	34	4·0	=0·54
Age-cohort[2]	-	-	-	24	3·8	<0·05	24	5·0	=0·76	24	0·7	=0·97
Age-period-cohort[3]	-	-	-	20	1·2	=0·62	20	1·6	=0·50	20	0·4	=0·99
Age-period[4]	-	-	-	30	17·2	=0·10	30	4·5	=0·98	30	2·2	=1·00
Age-drift[5]	-	-	-	34	25·4	=0·08	34	11·6	=0·13	34	4·0	=0·77
**DALY**	**df**	**dev**	**p**	**df**	**dev**	**p**	**df**	**dev**	**p**	**df**	**dev**	**p**
**Men**										-	-	-
Age	35	592·5	NA	35	16183·0	NA	35	815·8	NA	-	-	-
Age-drift[1]	34	464·6	<0·01	34	10985·0	<0·01	34	815·0	=0·37	-	-	-
Age-cohort[2]	24	47·1	<0·01	24	305·0	<0·01	24	224·4	<0·01	-	-	-
Age-period-cohort[3]	20	15·9	<0·01	20	204·0	<0·01	20	78·9	<0·01	-	-	-
Age-period[4]	30	303·0	<0·01	30	9276·0	<0·01	30	300·5	<0·01	-	-	-
Age-drift[5]	34	464·6	<0·01	34	10985·0	<0·01	34	815·0	<0·01	-	-	-
**Women**												
Age	-	-	-	35	3338·0	NA	35	297·2	NA	35	78·6	NA
Age-drift[1]	-	-	-	34	704·4	<0·01	34	278·6	<0·01	34	66·0	<0·01
Age-cohort[2]	-	-	-	24	92·4	<0·01	24	126·0	<0·01	24	18·5	<0·01
Age-period-cohort[3]	-	-	-	20	21·6	<0·01	20	29·2	<0·01	20	9·6	=0·06
Age-period[4]	-	-	-	30	485·9	<0·01	30	102·2	<0·01	30	44·5	<0·01
Age-drift[5]	-	-	-	34	704·4	<0·01	34	278·6	<0·01	34	66·0	<0·01
**Southern Latin America**												
**Models**	**Laryngeal cancer**			**Lung cancer**			**Mesothelioma**			**Ovarian cancer**		
**Mortality**	**df**	**dev**	**p**	**df**	**dev**	**p**	**df**	**dev**	**p**	**df**	**dev**	**p**
**Men**										-	-	-
Age	35	55·7	NA	35	827·9	NA	35	70·5	NA	-	-	-
Age-drift[1]	34	36·6	<0·01	34	737·3	<0·01	34	54·6	<0·01	-	-	-
Age-cohort[2]	24	10·8	<0·01	24	222·6	<0·01	24	35·2	<0·05	-	-	-
Age-period-cohort[3]	20	2·9	=0·09	20	55·7	<0·01	20	6·1	<0·01	-	-	-
Age-period[4]	30	17·2	=0·16	30	346·4	<0·01	30	11·3	=0·88	-	-	-
Age-drift[5]	34	36·6	<0·01	34	737·3	<0·01	34	54·6	<0·01	-	-	-
**Women**												
Age	-	-	-	35	501·5	NA	35	138·0	NA	35	60·9	NA
Age-drift[1]	-	-	-	34	112·7	<0·01	34	36·7	<0·01	34	35·4	<0·01
Age-cohort[2]	-	-	-	24	83·7	<0·01	24	27·7	=0·54	24	25·1	=0·42
Age-period-cohort[3]	-	-	-	20	4·5	<0·01	20	1·7	<0·01	20	1·5	<0·01
Age-period[4]	-	-	-	30	20·6	=0·09	30	2·8	=0·99	30	2·1	=0·99
Age-drift[5]	-	-	-	34	112·7	<0·01	34	36·7	<0·01	34	35·4	<0·01
**DALY**	**df**	**dev**	**p**	**df**	**dev**	**p**	**df**	**dev**	**p**	**df**	**dev**	**p**
**Men**										-	-	-
Age	35	1491·0	NA	35	22289·0	NA	35	1541·0	NA	-	-	-
Age-drift[1]	34	842·0	<0·01	34	17843·0	<0·01	34	1297·0	<0·01	-	-	-
Age-cohort[2]	24	234·0	<0·01	24	4872·0	<0·01	24	789·0	<0·01	-	-	-
Age-period-cohort[3]	20	73·0	<0·01	20	1181·0	<0·01	20	154·0	<0·01	-	-	-
Age-period[4]	30	448·0	<0·01	30	9212·0	<0·01	30	326·0	<0·01	-	-	-
Age-drift[5]	34	842·0	<0·01	34	17843·0	<0·01	34	1297·0	<0·01	-	-	-
**Women**												
Age	-	-	-	35	9885·1	NA	35	2945·0	NA	35	1178·6	NA
Age-drift[1]	-	-	-	34	2598·9	<0·01	34	830·3	<0·01	34	683·1	<0·01
Age-cohort[2]	-	-	-	24	1830·1	<0·01	24	622·8	<0·01	24	556·0	<0·01
Age-period-cohort[3]	-	-	-	20	93·2	<0·01	20	46·4	<0·01	20	33·4	<0·01
Age-period[4]	-	-	-	30	569·3	<0·01	30	78·5	<0·01	30	45·8	=0·25
Age-drift[5]	-	-	-	34	2598·9	<0·01	34	830·3	<0·01	34	683·1	<0·01

Table S2. Fit for the age-period-cohort models on mortality and disability-adjusted life years (DALY) age-specific groups rates for each cancer attributable to occupational asbestos exposure by sex and regions in the Americas, 1994 to 2023.

If significant ($p<0.05$), it represents: [1] non-linear age effect; [2] non-linear cohort effect; [3] non-linear period effect, in the presence of cohort; [4] non-linear cohort effect, in the presence of period; [5] non-linear period effect.

Lung cancer: trachea, bronchi and lungs cancer; df: degrees of freedom; dev: residual deviance; *p*: Chi-square *p* value. Elaborated by the authors (2025).

Male								Female							
		1990			2023					1990			2023		
		Value	95%UI		Value	95%UI				Value	95%UI		Value	95%UI	
**Antigua and Barbuda**															
Lung	Number	0·2	0·1	0·3	0·1	0·1	0·2	Lung	Number	0·0	0·0	0·0	0·0	0·0	0·0
Mesothelioma	Number	0·0	0·0	0·0	0·0	0·0	0·0	Ovary	Number	0·0	0·0	0·0	0·0	0·0	0·0
Larynx	Number	0·0	0·0	0·0	0·0	0·0	0·0	Mesothelioma	Number	0·0	0·0	0·0	0·0	0·0	0·0
Lung	Rate	0·7	0·4	1·1	0·2	0·1	0·4	Larynx	Number	0·0	0·0	0·0	0·0	0·0	0·0
Mesothelioma	Rate	0·1	0·1	0·1	0·0	0·0	0·1	Ovary	Rate	0·0	0·0	0·1	0·0	0·0	0·0
Larynx	Rate	0·0	0·0	0·1	0·0	0·0	0·0	Lung	Rate	0·0	0·0	0·1	0·0	0·0	0·0
								Mesothelioma	Rate	0·0	0·0	0·1	0·0	0·0	0·0
								Larynx	Rate	0·0	0·0	0·0	0·0	0·0	0·0
**Argentina**															
Lung	Number	736·5	433·2	1,099·6	1,334·9	850·3	1,899·6	Lung	Number	48·6	32·6	69·8	280·8	177·6	398·0
Larynx	Number	28·1	14·9	43·9	42·1	20·7	64·8	Larynx	Number	1·1	0·5	1·8	3·8	1·9	6·2
Mesothelioma	Number	56·6	49·6	65·8	148·6	130·8	170·1	Mesothelioma	Number	21·8	18·1	25·8	96·8	82·3	112·6
Lung	Rate	5·4	3·2	8·0	5·1	3·2	7·2	Ovary	Number	26·4	13·6	41·5	81·8	38·5	134·8
Larynx	Rate	0·2	0·1	0·3	0·2	0·1	0·2	Ovary	Rate	0·1	0·1	0·2	0·2	0·1	0·4
Mesothelioma	Rate	0·4	0·4	0·5	0·6	0·5	0·6	Lung	Rate	0·3	0·2	0·4	0·7	0·5	1·1
								Larynx	Rate	0·0	0·0	0·0	0·0	0·0	0·0
								Mesothelioma	Rate	0·1	0·1	0·1	0·3	0·2	0·3
**Bahamas**															
Lung	Number	1·6	0·9	2·4	3·1	1·8	5·0	Lung	Number	0·1	0·0	0·1	0·2	0·1	0·3
Mesothelioma	Number	0·3	0·2	0·3	0·6	0·5	0·7	Ovary	Number	0·1	0·0	0·1	0·2	0·1	0·3
Larynx	Number	0·1	0·0	0·2	0·2	0·1	0·4	Mesothelioma	Number	0·0	0·0	0·1	0·2	0·1	0·2
Lung	Rate	2·8	1·7	4·2	1·9	1·2	3·1	Larynx	Number	0·0	0·0	0·0	0·0	0·0	0·0
Mesothelioma	Rate	0·4	0·3	0·4	0·3	0·3	0·4	Ovary	Rate	0·1	0·0	0·1	0·1	0·0	0·1
Larynx	Rate	0·2	0·1	0·3	0·1	0·1	0·2	Lung	Rate	0·1	0·0	0·1	0·1	0·1	0·1
								Mesothelioma	Rate	0·0	0·0	0·1	0·1	0·1	0·1
								Larynx	Rate	0·0	0·0	0·0	0·0	0·0	0·0
**Barbados**															
Lung	Number	1·9	1·1	2·8	2·4	1·3	3·7	Lung	Number	0·1	0·1	0·2	0·3	0·2	0·5
Larynx	Number	0·1	0·0	0·2	0·2	0·1	0·3	Ovary	Number	0·1	0·1	0·3	0·3	0·1	0·5
Mesothelioma	Number	0·3	0·3	0·4	0·5	0·4	0·6	Larynx	Number	0·0	0·0	0·0	0·0	0·0	0·0
Lung	Rate	1·4	0·8	2·1	1·0	0·6	1·6	Mesothelioma	Number	0·2	0·2	0·2	0·2	0·2	0·3
Larynx	Rate	0·1	0·0	0·1	0·1	0·0	0·1	Lung	Rate	0·1	0·0	0·1	0·1	0·1	0·1
Mesothelioma	Rate	0·3	0·2	0·3	0·2	0·2	0·3	Ovary	Rate	0·1	0·0	0·1	0·1	0·0	0·2
								Larynx	Rate	0·0	0·0	0·0	0·0	0·0	0·0
								Mesothelioma	Rate	0·1	0·1	0·1	0·1	0·1	0·1
**Belize**															
Lung	Number	1·0	0·6	1·5	3·3	1·9	5·1	Lung	Number	0·0	0·0	0·1	0·2	0·1	0·3
Mesothelioma	Number	0·3	0·2	0·3	0·9	0·8	1·1	Ovary	Number	0·0	0·0	0·0	0·1	0·0	0·1
Larynx	Number	0·0	0·0	0·1	0·2	0·1	0·3	Mesothelioma	Number	0·1	0·0	0·1	0·2	0·2	0·3
Lung	Rate	2·4	1·5	3·5	2·5	1·5	3·8	Larynx	Number	0·0	0·0	0·0	0·0	0·0	0·0
Mesothelioma	Rate	0·6	0·5	0·8	0·6	0·5	0·7	Ovary	Rate	0·0	0·0	0·1	0·0	0·0	0·1
Larynx	Rate	0·1	0·1	0·2	0·1	0·1	0·2	Lung	Rate	0·1	0·1	0·1	0·1	0·1	0·2
								Mesothelioma	Rate	0·1	0·1	0·2	0·1	0·1	0·1
								Larynx	Rate	0·0	0·0	0·0	0·0	0·0	0·0

**Bermuda**															
Lung	Number	5·2	3·7	6·9	4·0	2·6	5·8	Lung	Number	0·4	0·2	0·6	0·4	0·2	0·6
Mesothelioma	Number	0·4	0·4	0·5	0·4	0·4	0·5	Mesothelioma	Number	0·1	0·1	0·2	0·1	0·1	0·2
Larynx	Number	0·2	0·1	0·2	0·1	0·1	0·2	Larynx	Number	0·0	0·0	0·0	0·0	0·0	0·0
Lung	Rate	21·2	15·3	27·7	6·4	4·1	9·3	Ovary	Number	0·1	0·1	0·2	0·1	0·1	0·3
Mesothelioma	Rate	1·6	1·4	1·8	0·7	0·6	0·9	Lung	Rate	1·2	0·7	1·7	0·4	0·2	0·7
Larynx	Rate	0·6	0·4	0·9	0·2	0·1	0·3	Ovary	Rate	0·4	0·2	0·7	0·2	0·1	0·3
								Mesothelioma	Rate	0·4	0·3	0·5	0·2	0·2	0·3
								Larynx	Rate	0·0	0·0	0·0	0·0	0·0	0·0
**Bolivia (Plurinational State of)**															
Lung	Number	15·3	7·2	29·4	90·7	43·6	162·8	Lung	Number	2·8	1·1	5·9	19·7	8·4	36·3
Mesothelioma	Number	3·3	2·0	5·1	20·2	11·4	29·3	Ovary	Number	0·5	0·2	1·1	6·7	2·4	13·7
Larynx	Number	0·5	0·2	0·9	3·6	1·4	7·0	Mesothelioma	Number	1·4	0·7	2·6	8·5	4·4	14·9
Lung	Rate	1·4	0·7	2·7	2·7	1·3	4·7	Larynx	Number	0·1	0·0	0·2	0·4	0·1	0·8
Mesothelioma	Rate	0·2	0·2	0·4	0·5	0·3	0·7	Ovary	Rate	0·0	0·0	0·1	0·1	0·0	0·3
Larynx	Rate	0·1	0·0	0·1	0·1	0·0	0·2	Lung	Rate	0·2	0·1	0·4	0·4	0·2	0·7
								Mesothelioma	Rate	0·1	0·0	0·2	0·2	0·1	0·3
								Larynx	Rate	0·0	0·0	0·0	0·0	0·0	0·0
**Brazil**															
Lung	Number	824·2	517·2	1,209·9	2,241·4	1,458·9	3,174·7	Lung	Number	190·4	130·6	256·1	983·8	635·6	1,358·0
Larynx	Number	53·3	27·3	82·4	142·9	73·8	225·4	Ovary	Number	63·2	30·1	98·0	228·9	108·3	362·7
Mesothelioma	Number	161·2	138·1	186·2	458·5	401·3	528·0	Larynx	Number	7·6	3·8	12·3	19·7	9·6	31·6
Lung	Rate	2·4	1·5	3·4	2·1	1·4	3·0	Mesothelioma	Number	118·8	97·8	139·5	341·3	271·6	407·8
Larynx	Rate	0·1	0·1	0·2	0·1	0·1	0·2	Ovary	Rate	0·2	0·1	0·2	0·2	0·1	0·2
Mesothelioma	Rate	0·4	0·3	0·4	0·4	0·3	0·5	Lung	Rate	0·5	0·3	0·7	0·7	0·4	0·9
								Larynx	Rate	0·0	0·0	0·0	0·0	0·0	0·0
								Mesothelioma	Rate	0·3	0·2	0·3	0·2	0·2	0·3
**Canada**															
Lung	Number	3,553·9	2,525·7	4,553·4	4,851·4	3,553·6	6,307·4	Lung	Number	228·2	142·7	327·0	801·5	515·8	1,145·8
Larynx	Number	60·5	33·5	88·0	69·6	39·8	101·3	Larynx	Number	1·9	0·9	3·1	2·6	1·2	4·4
Mesothelioma	Number	236·3	204·3	272·5	464·7	403·5	529·2	Mesothelioma	Number	42·6	35·3	51·0	102·4	84·3	119·3
Lung	Rate	25·3	18·2	32·1	12·6	9·2	16·3	Ovary	Number	58·8	27·3	97·1	136·1	63·2	217·9
Larynx	Rate	0·4	0·2	0·6	0·2	0·1	0·3	Lung	Rate	1·2	0·7	1·7	1·7	1·1	2·4
Mesothelioma	Rate	1·7	1·5	2·0	1·2	1·1	1·4	Ovary	Rate	0·3	0·1	0·5	0·3	0·1	0·5
								Larynx	Rate	0·0	0·0	0·0	0·0	0·0	0·0
**Chile**															
Lung	Number	165·9	102·3	239·4	322·3	199·3	468·0	Lung	Number	14·4	9·1	21·6	52·4	32·7	75·0
Larynx	Number	5·3	2·7	7·8	5·4	2·8	8·5	Larynx	Number	0·2	0·1	0·4	0·3	0·1	0·5
Mesothelioma	Number	19·7	17·2	23·0	49·5	43·2	56·7	Ovary	Number	5·2	2·5	8·7	13·8	6·2	22·4
Lung	Rate	4·1	2·6	5·9	2·7	1·7	3·9	Mesothelioma	Number	5·6	4·6	6·6	22·0	17·7	26·2
Larynx	Rate	0·1	0·1	0·2	0·0	0·0	0·1	Lung	Rate	0·3	0·2	0·4	0·3	0·2	0·5
Mesothelioma	Rate	0·5	0·4	0·5	0·4	0·4	0·5	Ovary	Rate	0·1	0·0	0·2	0·1	0·0	0·1
								Larynx	Rate	0·0	0·0	0·0	0·0	0·0	0·0
								Mesothelioma	Rate	0·1	0·1	0·1	0·1	0·1	0·2
**Colombia**															
Lung	Number	72·8	43·3	105·8	222·5	124·1	370·4	Lung	Number	15·0	9·5	20·9	49·8	31·4	73·9
Mesothelioma	Number	17·9	15·3	21·0	68·7	59·1	78·9	Ovary	Number	4·9	2·2	8·3	18·9	8·4	33·4
Larynx	Number	4·2	2·1	6·7	9·6	4·7	16·0	Mesothelioma	Number	9·8	8·1	11·5	31·1	25·4	37·3

Lung	Rate	1·0	0·6	1·4	0·9	0·5	1·4	Larynx	Number	0·9	0·4	1·4	0·7	0·3	1·1
Mesothelioma	Rate	0·2	0·2	0·3	0·3	0·2	0·3	Ovary	Rate	0·1	0·0	0·1	0·1	0·0	0·1
Larynx	Rate	0·1	0·0	0·1	0·0	0·0	0·1	Lung	Rate	0·2	0·1	0·3	0·1	0·1	0·2
								Mesothelioma	Rate	0·1	0·1	0·1	0·1	0·1	0·1
								Larynx	Rate	0·0	0·0	0·0	0·0	0·0	0·0
**Costa Rica**															
Lung	Number	6·7	3·8	10·5	15·6	8·7	24·8	Lung	Number	0·8	0·5	1·2	2·0	1·1	3·0
Larynx	Number	0·4	0·2	0·6	0·6	0·3	1·1	Larynx	Number	0·0	0·0	0·1	0·0	0·0	0·0
Mesothelioma	Number	0·8	0·7	1·0	3·2	2·7	3·7	Ovary	Number	0·1	0·1	0·2	0·7	0·3	1·4
Lung	Rate	1·0	0·6	1·5	0·6	0·4	1·0	Mesothelioma	Number	0·4	0·3	0·5	1·4	1·1	1·7
Larynx	Rate	0·1	0·0	0·1	0·0	0·0	0·0	Lung	Rate	0·1	0·1	0·1	0·1	0·0	0·1
Mesothelioma	Rate	0·1	0·1	0·1	0·1	0·1	0·1	Ovary	Rate	0·0	0·0	0·0	0·0	0·0	0·0
								Larynx	Rate	0·0	0·0	0·0	0·0	0·0	0·0
								Mesothelioma	Rate	0·0	0·0	0·1	0·0	0·0	0·1
**Cuba**															
Lung	Number	106·8	63·9	162·5	141·7	80·0	238·4	Lung	Number	10·3	6·3	14·9	24·1	14·1	36·4
Mesothelioma	Number	6·6	5·8	7·7	12·9	10·5	15·2	Ovary	Number	1·9	0·8	3·3	3·5	1·5	6·0
Larynx	Number	4·5	2·3	7·0	10·1	4·7	18·0	Mesothelioma	Number	4·3	3·5	5·1	4·8	3·8	6·0
Lung	Rate	2·0	1·2	3·0	1·5	0·8	2·5	Larynx	Number	0·4	0·2	0·6	0·5	0·2	0·9
Mesothelioma	Rate	0·1	0·1	0·1	0·1	0·1	0·2	Ovary	Rate	0·0	0·0	0·1	0·0	0·0	0·1
Larynx	Rate	0·1	0·0	0·1	0·1	0·0	0·2	Lung	Rate	0·2	0·1	0·3	0·2	0·1	0·3
								Mesothelioma	Rate	0·1	0·1	0·1	0·0	0·0	0·1
								Larynx	Rate	0·0	0·0	0·0	0·0	0·0	0·0
**Dominica**															
Lung	Number	0·8	0·4	1·3	1·7	0·9	3·0	Lung	Number	0·0	0·0	0·0	0·0	0·0	0·0
Mesothelioma	Number	0·1	0·1	0·1	0·2	0·2	0·3	Ovary	Number	0·0	0·0	0·0	0·0	0·0	0·0
Larynx	Number	0·0	0·0	0·1	0·1	0·0	0·1	Mesothelioma	Number	0·0	0·0	0·0	0·0	0·0	0·0
Lung	Rate	2·8	1·5	4·5	3·6	1·9	6·2	Larynx	Number	0·0	0·0	0·0	0·0	0·0	0·0
Mesothelioma	Rate	0·3	0·2	0·5	0·5	0·3	0·6	Ovary	Rate	0·0	0·0	0·0	0·0	0·0	0·0
Larynx	Rate	0·1	0·1	0·2	0·2	0·1	0·3	Lung	Rate	0·0	0·0	0·0	0·0	0·0	0·0
								Mesothelioma	Rate	0·0	0·0	0·0	0·0	0·0	0·0
								Larynx	Rate	0·0	0·0	0·0	0·0	0·0	0·0
**Dominican Republic**															
Lung	Number	8·3	4·5	14·7	38·6	19·6	70·0	Lung	Number	0·3	0·1	0·7	2·4	0·8	5·5
Mesothelioma	Number	2·0	1·2	3·0	5·7	3·7	8·2	Ovary	Number	0·1	0·0	0·1	0·4	0·1	0·9
Larynx	Number	0·4	0·2	0·8	1·7	0·7	3·2	Mesothelioma	Number	0·7	0·3	1·2	2·4	1·0	4·2
Lung	Rate	0·5	0·3	0·9	1·0	0·5	1·8	Larynx	Number	0·0	0·0	0·0	0·1	0·0	0·1
Mesothelioma	Rate	0·1	0·1	0·2	0·1	0·1	0·2	Ovary	Rate	0·0	0·0	0·0	0·0	0·0	0·0
Larynx	Rate	0·0	0·0	0·0	0·0	0·0	0·1	Lung	Rate	0·0	0·0	0·0	0·0	0·0	0·1
								Mesothelioma	Rate	0·0	0·0	0·1	0·0	0·0	0·1
								Larynx	Rate	0·0	0·0	0·0	0·0	0·0	0·0
**Ecuador**															
Lung	Number	11·7	6·9	17·2	40·4	23·5	61·6	Lung	Number	2·7	1·8	3·7	16·8	10·9	24·6
Mesothelioma	Number	3·5	3·0	4·1	11·2	9·5	13·0	Ovary	Number	0·2	0·1	0·3	6·6	3·1	11·6
Larynx	Number	0·5	0·3	0·8	1·3	0·6	2·0	Mesothelioma	Number	2·2	1·8	2·6	7·0	5·7	8·3
Lung	Rate	0·6	0·3	0·8	0·5	0·3	0·8	Larynx	Number	0·1	0·0	0·2	0·2	0·1	0·3
Mesothelioma	Rate	0·1	0·1	0·2	0·1	0·1	0·2	Ovary	Rate	0·0	0·0	0·0	0·1	0·0	0·1
Larynx	Rate	0·0	0·0	0·0	0·0	0·0	0·0	Lung	Rate	0·1	0·1	0·2	0·2	0·1	0·3

								Mesothelioma	Rate	0·1	0·1	0·1	0·1	0·1	0·1
								Larynx	Rate	0·0	0·0	0·0	0·0	0·0	0·0
**El Salvador**															
Lung	Number	0·2	0·0	0·4	2·2	1·0	4·0	Lung	Number	0·0	0·0	0·0	0·4	0·1	0·7
Larynx	Number	0·0	0·0	0·0	0·1	0·0	0·2	Ovary	Number	0·0	0·0	0·0	0·1	0·0	0·3
Mesothelioma	Number	0·3	0·2	0·5	1·3	0·8	1·9	Larynx	Number	0·0	0·0	0·0	0·0	0·0	0·0
Lung	Rate	0·0	0·0	0·0	0·1	0·0	0·2	Mesothelioma	Number	0·2	0·1	0·3	0·9	0·4	1·4
Larynx	Rate	0·0	0·0	0·0	0·0	0·0	0·0	Ovary	Rate	0·0	0·0	0·0	0·0	0·0	0·0
Mesothelioma	Rate	0·0	0·0	0·0	0·1	0·0	0·1	Lung	Rate	0·0	0·0	0·0	0·0	0·0	0·0
								Larynx	Rate	0·0	0·0	0·0	0·0	0·0	0·0
								Mesothelioma	Rate	0·0	0·0	0·0	0·0	0·0	0·0
**Greenland**															
Lung	Number	0·6	0·3	1·1	9·1	5·1	13·5	Lung	Number	1·1	0·5	2·2	2·0	0·9	3·5
Mesothelioma	Number	0·0	0·0	0·0	0·5	0·3	0·6	Ovary	Number	0·1	0·1	0·3	0·2	0·1	0·4
Larynx	Number	0·0	0·0	0·0	0·1	0·1	0·2	Mesothelioma	Number	0·0	0·0	0·1	0·1	0·1	0·2
Lung	Rate	7·3	3·7	13·1	34·8	19·9	50·4	Larynx	Number	0·0	0·0	0·0	0·0	0·0	0·0
Mesothelioma	Rate	0·2	0·2	0·3	1·9	1·3	2·5	Ovary	Rate	0·9	0·4	1·8	0·8	0·3	1·5
Larynx	Rate	0·1	0·0	0·1	0·5	0·2	0·9	Lung	Rate	7·2	3·3	15·2	6·9	3·1	11·9
								Mesothelioma	Rate	0·2	0·1	0·3	0·4	0·2	0·7
								Larynx	Rate	0·0	0·0	0·0	0·0	0·0	0·0
**Grenada**															
Lung	Number	1·0	0·6	1·5	0·9	0·5	1·3	Lung	Number	0·1	0·1	0·1	0·2	0·1	0·2
Larynx	Number	0·0	0·0	0·1	0·1	0·0	0·1	Ovary	Number	0·1	0·0	0·1	0·1	0·1	0·2
Mesothelioma	Number	0·1	0·1	0·1	0·2	0·1	0·2	Larynx	Number	0·0	0·0	0·0	0·0	0·0	0·0
Lung	Rate	3·3	2·1	4·8	1·1	0·7	1·7	Mesothelioma	Number	0·1	0·1	0·1	0·1	0·1	0·1
Larynx	Rate	0·1	0·1	0·2	0·1	0·0	0·1	Lung	Rate	0·2	0·1	0·3	0·2	0·1	0·3
Mesothelioma	Rate	0·3	0·3	0·4	0·2	0·2	0·3	Ovary	Rate	0·1	0·1	0·2	0·2	0·1	0·3
								Larynx	Rate	0·0	0·0	0·0	0·0	0·0	0·0
								Mesothelioma	Rate	0·2	0·2	0·3	0·1	0·1	0·2
**Guatemala**															
Lung	Number	3·2	1·8	5·0	8·9	4·8	15·0	Lung	Number	0·7	0·4	1·1	2·1	1·2	3·4
Larynx	Number	0·2	0·1	0·3	0·3	0·1	0·5	Ovary	Number	0·1	0·0	0·1	0·7	0·3	1·3
Mesothelioma	Number	1·5	1·2	1·8	3·8	2·9	4·7	Larynx	Number	0·0	0·0	0·1	0·0	0·0	0·1
Lung	Rate	0·3	0·2	0·4	0·2	0·1	0·3	Mesothelioma	Number	0·7	0·6	0·9	2·3	1·8	3·0
Larynx	Rate	0·0	0·0	0·0	0·0	0·0	0·0	Ovary	Rate	0·0	0·0	0·0	0·0	0·0	0·0
Mesothelioma	Rate	0·1	0·1	0·1	0·1	0·1	0·1	Lung	Rate	0·0	0·0	0·1	0·0	0·0	0·1
								Larynx	Rate	0·0	0·0	0·0	0·0	0·0	0·0
								Mesothelioma	Rate	0·0	0·0	0·1	0·0	0·0	0·0
**Guyana**															
Lung	Number	1·1	0·6	1·7	2·3	1·3	3·7	Lung	Number	0·1	0·1	0·2	0·5	0·3	0·8
Larynx	Number	0·1	0·0	0·1	0·2	0·1	0·3	Ovary	Number	0·1	0·1	0·2	0·5	0·3	1·0
Mesothelioma	Number	0·3	0·3	0·4	0·9	0·7	1·1	Larynx	Number	0·0	0·0	0·0	0·0	0·0	0·0
Lung	Rate	0·7	0·4	1·1	0·7	0·4	1·1	Mesothelioma	Number	0·3	0·2	0·4	1·0	0·7	1·3
Larynx	Rate	0·0	0·0	0·1	0·0	0·0	0·1	Ovary	Rate	0·1	0·0	0·1	0·1	0·1	0·3
Mesothelioma	Rate	0·2	0·2	0·2	0·3	0·2	0·3	Lung	Rate	0·1	0·0	0·1	0·1	0·1	0·2
								Larynx	Rate	0·0	0·0	0·0	0·0	0·0	0·0
								Mesothelioma	Rate	0·1	0·1	0·2	0·2	0·2	0·3
**Haiti**															

Lung	Number	14·2	6·4	27·5	42·0	20·5	76·8	Lung	Number	1·0	0·4	2·5	3·6	1·1	7·4
Larynx	Number	1·0	0·4	1·8	3·3	1·3	6·5	Ovary	Number	0·5	0·1	1·4	2·6	0·7	6·1
Mesothelioma	Number	2·8	1·7	4·4	10·0	5·9	14·7	Larynx	Number	0·0	0·0	0·1	0·1	0·0	0·3
Lung	Rate	1·1	0·5	2·2	1·6	0·8	2·9	Mesothelioma	Number	1·3	0·5	2·4	4·7	2·0	8·5
Larynx	Rate	0·1	0·0	0·1	0·1	0·0	0·2	Ovary	Rate	0·0	0·0	0·1	0·1	0·0	0·1
Mesothelioma	Rate	0·2	0·1	0·3	0·3	0·2	0·4	Lung	Rate	0·1	0·0	0·2	0·1	0·0	0·2
								Larynx	Rate	0·0	0·0	0·0	0·0	0·0	0·0
								Mesothelioma	Rate	0·1	0·0	0·1	0·1	0·0	0·2
**Honduras**															
Lung	Number	5·2	2·3	9·4	33·7	14·6	56·0	Lung	Number	1·2	0·4	2·1	12·2	4·3	23·8
Larynx	Number	0·2	0·1	0·4	1·4	0·6	2·7	Larynx	Number	0·0	0·0	0·1	0·3	0·1	0·6
Mesothelioma	Number	1·4	0·9	2·2	6·6	3·9	9·5	Mesothelioma	Number	0·6	0·2	1·1	3·9	1·4	6·2
Lung	Rate	0·6	0·3	1·2	1·2	0·5	2·0	Ovary	Number	0·3	0·1	0·6	3·6	1·0	7·6
Larynx	Rate	0·0	0·0	0·0	0·0	0·0	0·1	Lung	Rate	0·1	0·0	0·3	0·4	0·1	0·7
Mesothelioma	Rate	0·1	0·1	0·2	0·2	0·1	0·3	Ovary	Rate	0·0	0·0	0·1	0·1	0·0	0·2
								Larynx	Rate	0·0	0·0	0·0	0·0	0·0	0·0
								Mesothelioma	Rate	0·1	0·0	0·1	0·1	0·0	0·2
**Jamaica**															
Lung	Number	17·7	10·9	26·9	24·5	13·6	41·3	Lung	Number	1·0	0·6	1·5	0·9	0·5	1·4
Larynx	Number	0·4	0·2	0·7	0·8	0·4	1·4	Larynx	Number	0·0	0·0	0·0	0·0	0·0	0·0
Mesothelioma	Number	1·9	1·6	2·2	3·8	3·1	4·6	Ovary	Number	0·5	0·2	0·8	0·4	0·2	0·7
Lung	Rate	2·1	1·3	3·2	1·7	0·9	2·8	Mesothelioma	Number	1·0	0·8	1·3	1·4	1·1	1·8
Larynx	Rate	0·0	0·0	0·1	0·1	0·0	0·1	Lung	Rate	0·1	0·1	0·1	0·0	0·0	0·1
Mesothelioma	Rate	0·2	0·2	0·3	0·3	0·2	0·3	Ovary	Rate	0·0	0·0	0·1	0·0	0·0	0·0
								Larynx	Rate	0·0	0·0	0·0	0·0	0·0	0·0
								Mesothelioma	Rate	0·1	0·1	0·1	0·1	0·1	0·1
**Mexico**															
Lung	Number	319·8	196·7	467·9	531·5	313·8	789·0	Lung	Number	53·0	36·1	73·0	116·5	72·1	167·8
Larynx	Number	16·6	8·6	25·6	22·5	11·2	35·0	Ovary	Number	17·9	8·8	28·0	79·5	38·0	131·2
Mesothelioma	Number	59·5	51·6	68·7	189·4	165·7	217·0	Larynx	Number	1·9	0·9	3·0	1·6	0·8	2·8
Lung	Rate	1·8	1·1	2·6	0·9	0·5	1·3	Mesothelioma	Number	29·2	24·4	34·3	95·0	79·0	113·2
Larynx	Rate	0·1	0·0	0·1	0·0	0·0	0·1	Ovary	Rate	0·1	0·0	0·1	0·1	0·1	0·2
Mesothelioma	Rate	0·3	0·3	0·3	0·3	0·3	0·3	Lung	Rate	0·3	0·2	0·4	0·2	0·1	0·2
								Larynx	Rate	0·0	0·0	0·0	0·0	0·0	0·0
								Mesothelioma	Rate	0·1	0·1	0·2	0·1	0·1	0·2
**Nicaragua**															
Lung	Number	0·8	0·4	1·3	2·8	1·4	5·2	Lung	Number	0·1	0·0	0·2	0·9	0·3	1·5
Larynx	Number	0·1	0·0	0·1	0·2	0·1	0·4	Ovary	Number	0·0	0·0	0·0	0·4	0·1	0·7
Mesothelioma	Number	0·5	0·4	0·8	2·5	1·7	3·2	Larynx	Number	0·0	0·0	0·0	0·0	0·0	0·0
Lung	Rate	0·1	0·1	0·2	0·2	0·1	0·3	Mesothelioma	Number	0·3	0·1	0·5	1·4	0·7	2·5
Larynx	Rate	0·0	0·0	0·0	0·0	0·0	0·0	Ovary	Rate	0·0	0·0	0·0	0·0	0·0	0·0
Mesothelioma	Rate	0·1	0·1	0·1	0·1	0·1	0·2	Lung	Rate	0·0	0·0	0·0	0·0	0·0	0·1
								Larynx	Rate	0·0	0·0	0·0	0·0	0·0	0·0
								Mesothelioma	Rate	0·0	0·0	0·1	0·1	0·0	0·1
**Panama**															
Lung	Number	3·7	2·1	5·9	7·8	4·3	13·4	Lung	Number	0·6	0·3	0·8	1·3	0·8	2·2
Mesothelioma	Number	0·6	0·5	0·7	1·6	1·3	2·0	Mesothelioma	Number	0·4	0·3	0·5	1·3	1·0	1·6
Larynx	Number	0·2	0·1	0·3	0·3	0·2	0·5	Larynx	Number	0·0	0·0	0·0	0·0	0·0	0·0

Lung	Rate	0·6	0·3	0·9	0·4	0·2	0·6	Ovary	Number	0·1	0·0	0·2	0·5	0·2	1·0
Mesothelioma	Rate	0·1	0·1	0·1	0·1	0·1	0·1	Lung	Rate	0·1	0·1	0·1	0·1	0·0	0·1
Larynx	Rate	0·0	0·0	0·0	0·0	0·0	0·0	Ovary	Rate	0·0	0·0	0·0	0·0	0·0	0·0
								Mesothelioma	Rate	0·1	0·0	0·1	0·1	0·0	0·1
								Larynx	Rate	0·0	0·0	0·0	0·0	0·0	0·0
**Paraguay**															
Lung	Number	7·7	4·3	12·2	57·5	25·7	93·2	Lung	Number	1·3	0·6	2·3	10·4	4·0	18·3
Larynx	Number	0·3	0·1	0·5	1·9	0·8	3·4	Ovary	Number	0·4	0·2	0·8	3·7	1·3	6·8
Mesothelioma	Number	1·9	1·4	2·5	8·2	5·9	10·6	Larynx	Number	0·0	0·0	0·1	0·2	0·1	0·4
Lung	Rate	0·9	0·5	1·4	2·8	1·3	4·4	Mesothelioma	Number	1·4	0·8	2·4	6·0	3·5	9·7
Larynx	Rate	0·0	0·0	0·1	0·1	0·0	0·2	Ovary	Rate	0·0	0·0	0·1	0·1	0·0	0·2
Mesothelioma	Rate	0·2	0·1	0·3	0·3	0·2	0·4	Lung	Rate	0·1	0·1	0·2	0·4	0·1	0·7
								Larynx	Rate	0·0	0·0	0·0	0·0	0·0	0·0
								Mesothelioma	Rate	0·1	0·1	0·2	0·2	0·1	0·3
**Peru**															
Lung	Number	85·9	43·7	140·0	149·2	77·9	250·8	Lung	Number	17·7	7·1	31·4	50·8	20·2	99·4
Larynx	Number	2·6	1·1	4·5	5·0	1·9	9·4	Ovary	Number	3·3	1·2	6·4	16·0	5·3	35·7
Mesothelioma	Number	14·7	9·7	22·3	47·5	29·6	65·3	Larynx	Number	0·3	0·1	0·7	0·5	0·2	1·2
Lung	Rate	1·8	0·9	2·9	0·9	0·5	1·6	Mesothelioma	Number	8·2	4·2	13·1	31·2	16·7	51·8
Larynx	Rate	0·1	0·0	0·1	0·0	0·0	0·1	Lung	Rate	0·3	0·1	0·6	0·3	0·1	0·5
Mesothelioma	Rate	0·3	0·2	0·4	0·3	0·2	0·4	Ovary	Rate	0·1	0·0	0·1	0·1	0·0	0·2
								Larynx	Rate	0·0	0·0	0·0	0·0	0·0	0·0
								Mesothelioma	Rate	0·1	0·1	0·2	0·2	0·1	0·3
**Puerto Rico**															
Lung	Number	40·0	24·7	60·4	49·5	28·4	79·3	Lung	Number	1·4	0·8	2·0	2·8	1·7	4·5
Mesothelioma	Number	6·4	5·5	7·4	7·6	6·5	8·8	Ovary	Number	0·4	0·2	0·7	1·2	0·6	2·3
Larynx	Number	2·1	1·1	3·3	1·6	0·8	2·7	Mesothelioma	Number	0·8	0·6	0·9	2·2	1·8	2·6
Lung	Rate	2·3	1·4	3·4	1·1	0·7	1·8	Larynx	Number	0·0	0·0	0·1	0·0	0·0	0·0
Mesothelioma	Rate	0·4	0·3	0·4	0·2	0·2	0·2	Ovary	Rate	0·0	0·0	0·0	0·0	0·0	0·1
Larynx	Rate	0·1	0·1	0·2	0·0	0·0	0·1	Lung	Rate	0·1	0·0	0·1	0·1	0·0	0·1
								Mesothelioma	Rate	0·0	0·0	0·0	0·1	0·0	0·1
								Larynx	Rate	0·0	0·0	0·0	0·0	0·0	0·0
**Saint Kitts and Nevis**															
Lung	Number	0·8	0·5	1·0	0·6	0·4	0·9	Lung	Number	0·0	0·0	0·0	0·0	0·0	0·0
Mesothelioma	Number	0·2	0·1	0·2	0·3	0·2	0·3	Ovary	Number	0·0	0·0	0·0	0·0	0·0	0·0
Larynx	Number	0·1	0·0	0·1	0·1	0·0	0·1	Mesothelioma	Number	0·0	0·0	0·0	0·0	0·0	0·0
Lung	Rate	4·4	3·0	6·2	2·7	1·8	3·8	Larynx	Number	0·0	0·0	0·0	0·0	0·0	0·0
Mesothelioma	Rate	1·1	0·9	1·2	1·1	0·9	1·2	Ovary	Rate	0·1	0·0	0·1	0·1	0·0	0·1
Larynx	Rate	0·3	0·2	0·6	0·3	0·1	0·4	Lung	Rate	0·1	0·0	0·1	0·1	0·0	0·1
								Mesothelioma	Rate	0·1	0·1	0·1	0·1	0·0	0·1
								Larynx	Rate	0·0	0·0	0·0	0·0	0·0	0·0
**Saint Lucia**															
Lung	Number	0·5	0·3	0·8	0·5	0·3	0·8	Lung	Number	0·1	0·1	0·1	0·1	0·1	0·2
Mesothelioma	Number	0·1	0·1	0·1	0·1	0·1	0·1	Mesothelioma	Number	0·1	0·1	0·1	0·1	0·1	0·1
Larynx	Number	0·0	0·0	0·1	0·0	0·0	0·1	Larynx	Number	0·0	0·0	0·0	0·0	0·0	0·0
Lung	Rate	1·4	0·8	2·2	0·4	0·2	0·7	Ovary	Number	0·1	0·0	0·1	0·1	0·1	0·2
Mesothelioma	Rate	0·2	0·2	0·2	0·1	0·1	0·1	Ovary	Rate	0·1	0·1	0·2	0·1	0·0	0·2
Larynx	Rate	0·1	0·0	0·1	0·0	0·0	0·1	Lung	Rate	0·2	0·1	0·3	0·1	0·1	0·2

								Mesothelioma	Rate	0·2	0·2	0·2	0·1	0·1	0·1
								Larynx	Rate	0·0	0·0	0·0	0·0	0·0	0·0
**Saint Vincent and the Grenadines**															
Lung	Number	0·3	0·2	0·5	0·4	0·3	0·7	Lung	Number	0·1	0·0	0·1	0·1	0·1	0·1
Larynx	Number	0·0	0·0	0·0	0·1	0·0	0·1	Ovary	Number	0·0	0·0	0·1	0·1	0·0	0·1
Mesothelioma	Number	0·1	0·1	0·1	0·1	0·1	0·1	Larynx	Number	0·0	0·0	0·0	0·0	0·0	0·0
Lung	Rate	1·1	0·6	1·7	0·5	0·3	0·8	Mesothelioma	Number	0·2	0·2	0·2	0·2	0·2	0·2
Larynx	Rate	0·1	0·0	0·2	0·1	0·0	0·1	Ovary	Rate	0·1	0·0	0·2	0·1	0·0	0·1
Mesothelioma	Rate	0·2	0·2	0·3	0·1	0·1	0·2	Lung	Rate	0·1	0·1	0·2	0·1	0·1	0·2
								Larynx	Rate	0·0	0·0	0·0	0·0	0·0	0·0
								Mesothelioma	Rate	0·5	0·4	0·6	0·3	0·2	0·3
**Suriname**															
Lung	Number	0·6	0·3	1·0	1·9	0·8	3·2	Lung	Number	0·1	0·0	0·2	0·5	0·2	1·0
Larynx	Number	0·0	0·0	0·0	0·0	0·0	0·1	Ovary	Number	0·1	0·0	0·1	0·3	0·1	0·5
Mesothelioma	Number	0·1	0·1	0·1	0·3	0·2	0·5	Larynx	Number	0·0	0·0	0·0	0·0	0·0	0·0
Lung	Rate	0·5	0·3	0·9	0·7	0·3	1·2	Mesothelioma	Number	0·1	0·0	0·2	0·4	0·2	0·6
Larynx	Rate	0·0	0·0	0·0	0·0	0·0	0·0	Lung	Rate	0·1	0·0	0·2	0·1	0·1	0·3
Mesothelioma	Rate	0·1	0·0	0·1	0·1	0·1	0·2	Ovary	Rate	0·0	0·0	0·1	0·1	0·0	0·1
								Larynx	Rate	0·0	0·0	0·0	0·0	0·0	0·0
								Mesothelioma	Rate	0·1	0·0	0·1	0·1	0·1	0·2
**Trinidad and Tobago**															
Lung	Number	4·7	2·8	7·1	7·6	4·0	13·0	Lung	Number	0·3	0·2	0·5	0·7	0·4	1·1
Mesothelioma	Number	0·9	0·8	1·1	2·0	1·7	2·4	Ovary	Number	0·4	0·2	0·7	0·8	0·4	1·4
Larynx	Number	0·2	0·1	0·3	0·3	0·2	0·6	Mesothelioma	Number	0·3	0·2	0·3	0·7	0·6	0·9
Lung	Rate	1·6	0·9	2·4	1·0	0·5	1·7	Larynx	Number	0·0	0·0	0·0	0·0	0·0	0·0
Mesothelioma	Rate	0·3	0·2	0·3	0·2	0·2	0·3	Ovary	Rate	0·1	0·0	0·2	0·1	0·0	0·1
Larynx	Rate	0·1	0·0	0·1	0·0	0·0	0·1	Lung	Rate	0·1	0·1	0·1	0·1	0·0	0·1
								Mesothelioma	Rate	0·1	0·1	0·1	0·1	0·1	0·1
								Larynx	Rate	0·0	0·0	0·0	0·0	0·0	0·0
**United States of America**															
Lung	Number	27,252·2	19,325·3	35,788·5	23,170·8	16,715·4	31,350·3	Lung	Number	2,700·8	1,793·0	3,570·7	4,373·2	2,796·7	5,992·1
Larynx	Number	345·0	188·4	504·8	377·4	203·9	562·1	Ovary	Number	634·3	307·1	991·4	782·7	368·8	1,217·3
Mesothelioma	Number	1,507·1	1,322·0	1,728·0	1,834·0	1,601·1	2,105·7	Larynx	Number	17·9	8·8	28·8	21·4	11·1	34·4
Lung	Rate	19·9	14·1	26·1	8·1	5·8	10·9	Mesothelioma	Number	398·9	323·6	477·2	645·4	512·6	772·8
Larynx	Rate	0·3	0·1	0·4	0·1	0·1	0·2	Ovary	Rate	0·3	0·1	0·5	0·2	0·1	0·3
Mesothelioma	Rate	1·1	1·0	1·3	0·7	0·6	0·8	Lung	Rate	1·3	0·9	1·7	1·2	0·8	1·6
								Larynx	Rate	0·0	0·0	0·0	0·0	0·0	0·0
								Mesothelioma	Rate	0·2	0·2	0·2	0·2	0·1	0·2
**United States Virgin Islands**															
Lung	Number	1·2	0·7	1·8	3·2	2·1	4·9	Lung	Number	0·1	0·1	0·2	0·3	0·2	0·6
Mesothelioma	Number	0·3	0·2	0·3	0·5	0·4	0·5	Ovary	Number	0·0	0·0	0·1	0·2	0·1	0·4
Larynx	Number	0·0	0·0	0·1	0·1	0·1	0·2	Mesothelioma	Number	0·1	0·1	0·1	0·1	0·1	0·2
Lung	Rate	4·9	3·1	7·1	3·4	2·2	5·2	Larynx	Number	0·0	0·0	0·0	0·0	0·0	0·0
Mesothelioma	Rate	0·8	0·7	1·0	0·6	0·5	0·7	Ovary	Rate	0·1	0·0	0·2	0·2	0·1	0·4
Larynx	Rate	0·2	0·1	0·3	0·1	0·1	0·2	Lung	Rate	0·4	0·2	0·6	0·3	0·1	0·5
								Mesothelioma	Rate	0·2	0·1	0·2	0·2	0·1	0·2

								Larynx	Rate	0·0	0·0	0·0	0·0	0·0	0·0
**Uruguay**															
Lung	Number	114·3	72·1	170·7	154·5	97·2	224·1	Lung	Number	3·1	2·1	4·6	15·9	9·5	22·7
Larynx	Number	4·4	2·2	7·0	4·7	2·5	7·2	Ovary	Number	1·8	0·8	3·0	5·0	2·3	8·4
Mesothelioma	Number	5·6	4·9	6·5	10·9	9·5	12·5	Larynx	Number	0·1	0·0	0·1	0·2	0·1	0·3
Lung	Rate	7·0	4·5	10·3	6·4	4·0	9·3	Mesothelioma	Number	1·4	1·2	1·7	5·0	4·1	6·0
Larynx	Rate	0·3	0·1	0·4	0·2	0·1	0·3	Ovary	Rate	0·1	0·0	0·1	0·1	0·1	0·2
Mesothelioma	Rate	0·3	0·3	0·4	0·5	0·4	0·5	Lung	Rate	0·1	0·1	0·2	0·4	0·3	0·6
								Larynx	Rate	0·0	0·0	0·0	0·0	0·0	0·0
								Mesothelioma	Rate	0·1	0·1	0·1	0·2	0·1	0·2
**Venezuela (Bolivarian Republic of)**															
Lung	Number	30·5	17·4	47·6	94·8	47·1	173·3	Lung	Number	9·0	6·0	13·2	21·6	12·0	37·5
Larynx	Number	1·7	0·8	2·8	4·2	1·8	7·6	Ovary	Number	0·7	0·4	1·3	7·2	3·4	12·6
Mesothelioma	Number	6·0	5·2	7·1	21·3	17·1	26·4	Larynx	Number	0·4	0·2	0·6	0·4	0·2	0·8
Lung	Rate	0·8	0·5	1·3	0·8	0·4	1·4	Mesothelioma	Number	4·3	3·5	5·1	12·5	9·7	15·0
Larynx	Rate	0·1	0·0	0·1	0·0	0·0	0·1	Ovary	Rate	0·0	0·0	0·0	0·0	0·0	0·1
Mesothelioma	Rate	0·1	0·1	0·2	0·2	0·1	0·2	Lung	Rate	0·2	0·1	0·3	0·1	0·1	0·2
								Larynx	Rate	0·0	0·0	0·0	0·0	0·0	0·0
								Mesothelioma	Rate	0·1	0·1	0·1	0·1	0·1	0·1

Table S3. Mortality numbers and rates for cancer attributable to occupational asbestos exposure, by sex, cancer type and country, in 1990 and 2023 in the Americas.
Number: number of deaths for all ages; Rate: age-standardized mortality rates per 100,000; 95%UI: 95% uncertainty interval.
Elaborated by the authors (2025).

		Male								Female					
		1990			2023					1990			2023		
		Value	95%UI		Value	95%UI				Value	95%UI		Value	95%UI	
**Antigua and Barbuda**															
Lung	Number	3·0	1·8	4·6	2·1	1·2	3·6	Lung	Number	0·2	0·1	0·3	0·3	0·2	0·5
Larynx	Number	0·2	0·1	0·3	0·2	0·1	0·3	Ovary	Number	0·2	0·1	0·3	0·3	0·1	0·6
Mesothelioma	Number	0·6	0·5	0·7	0·6	0·4	0·7	Larynx	Number	0·0	0·0	0·0	0·0	0·0	0·0
Lung	Rate	12·8	7·7	19·8	4·1	2·3	7·0	Mesothelioma	Number	0·3	0·3	0·4	0·2	0·2	0·3
Larynx	Rate	0·7	0·4	1·2	0·4	0·2	0·6	Lung	Rate	0·6	0·4	1·0	0·5	0·3	0·8
Mesothelioma	Rate	2·6	2·1	3·2	1·0	0·7	1·2	Ovary	Rate	0·6	0·3	1·1	0·5	0·2	1·0
								Larynx	Rate	0·0	0·0	0·0	0·0	0·0	0·0
								Mesothelioma	Rate	1·2	1·0	1·5	0·4	0·3	0·5
**Argentina**															
Lung	Number	16,124·3	9,424·2	24,012·6	25,020·9	15,515·1	36,218·8	Lung	Number	948·1	629·4	1,377·7	5,134·2	3,303·9	7,285·2
Larynx	Number	652·5	346·7	1,034·5	826·3	404·1	1,281·1	Larynx	Number	21·5	10·4	35·9	70·6	33·6	114·1
Mesothelioma	Number	1,463·5	1,269·8	1,696·9	3,471·2	3,046·2	3,992·4	Mesothelioma	Number	537·1	449·9	636·1	2,238·1	1,898·5	2,627·5
Lung	Rate	111·9	65·9	166·4	93·8	58·4	135·9	Ovary	Number	549·9	284·6	873·5	1,606·1	758·1	2,616·5
Larynx	Rate	4·5	2·4	7·1	3·1	1·5	15·1	Lung	Rate	5·1	3·4	7·4	14·5	9·3	20·8
Mesothelioma	Rate	9·9	8·6	11·5	13·2	11·6	15·1	Larynx	Rate	0·1	0·1	0·2	0·2	0·1	0·3
								Mesothelioma	Rate	3·0	2·5	3·6	7·0	5·9	8·2
								Ovary	Rate	3·0	1·5	4·7	4·7	2·2	7·6
**Bahamas**															
Lung	Number	36·9	21·1	55·9	65·5	35·4	108·1	Lung	Number	1·6	1·0	2·5	5·0	2·8	8·2
Mesothelioma	Number	8·3	6·9	9·9	17·7	14·5	21·4	Ovary	Number	1·7	0·7	3·0	4·8	1·9	8·8
Larynx	Number	2·3	1·1	3·6	5·1	2·1	9·1	Mesothelioma	Number	1·4	1·1	1·8	5·5	4·3	7·0
Lung	Rate	57·3	33·1	86·6	35·0	19·8	56·9	Larynx	Number	0·0	0·0	0·1	0·1	0·0	0·2
Mesothelioma	Rate	10·4	8·8	12·2	8·1	6·7	9·6	Lung	Rate	1·8	1·1	2·9	2·0	1·1	3·3
Larynx	Rate	3·5	1·7	5·5	2·7	1·1	4·8	Ovary	Rate	1·8	0·8	3·2	1·9	0·7	3·4
								Mesothelioma	Rate	1·4	1·1	1·8	2·1	1·7	2·7
								Larynx	Rate	0·1	0·0	0·1	0·0	0·0	0·1
**Barbados**															
Lung	Number	31·7	18·3	48·1	42·1	22·7	66·5	Lung	Number	2·5	1·6	3·9	5·6	3·3	8·5
Larynx	Number	1·6	0·9	2·8	3·5	1·7	5·8	Larynx	Number	0·1	0·0	0·1	0·1	0·1	0·2
Mesothelioma	Number	7·0	5·9	8·2	11·5	9·4	13·8	Ovary	Number	2·9	1·2	5·0	5·4	2·3	9·7
Lung	Rate	24·5	14·0	37·5	17·5	9·5	27·5	Mesothelioma	Number	5·2	4·2	6·2	6·2	5·0	7·7
Larynx	Rate	1·3	0·7	2·2	1·4	0·7	2·4	Lung	Rate	1·5	0·9	2·3	1·8	1·1	2·8
Mesothelioma	Rate	6·2	5·2	7·3	4·9	4·0	6·0	Larynx	Rate	0·0	0·0	0·1	0·0	0·0	0·1
								Ovary	Rate	1·8	0·8	3·2	1·9	0·8	3·3
								Mesothelioma	Rate	3·8	3·1	4·5	2·5	2·0	3·1
**Belize**															
Lung	Number	20·1	12·1	29·2	71·4	41·3	112·5	Lung	Number	0·8	0·5	1·2	4·6	2·8	7·6
Mesothelioma	Number	8·1	6·1	10·0	27·2	22·4	33·0	Ovary	Number	0·4	0·2	0·6	1·8	0·8	3·3
Larynx	Number	0·9	0·4	1·4	3·9	1·9	6·4	Mesothelioma	Number	2·2	1·5	3·2	7·4	5·9	9·4
Lung	Rate	45·6	27·4	66·1	47·9	28·0	74·6	Larynx	Number	0·0	0·0	0·1	0·1	0·0	0·2
Mesothelioma	Rate	16·5	13·0	20·0	15·9	13·2	19·1	Lung	Rate	1·8	1·1	2·7	2·8	1·7	4·6
Larynx	Rate	1·9	1·0	3·2	2·6	1·3	4·2	Ovary	Rate	0·8	0·3	1·4	1·0	0·4	1·8
								Mesothelioma	Rate	4·4	3·2	6·4	3·8	3·1	4·9
								Larynx	Rate	0·1	0·0	0·1	0·1	0·0	0·1

**Bermuda**															
Lung	Number	105·6	72·5	144·2	67·9	42·9	101·5	Lung	Number	7·3	4·5	10·6	5·5	3·3	8·9
Larynx	Number	3·2	1·7	4·9	2·2	1·1	3·4	Ovary	Number	2·9	1·3	5·1	2·4	1·1	4·5
Mesothelioma	Number	11·9	10·2	13·8	10·2	8·8	12·0	Larynx	Number	0·1	0·0	0·1	0·0	0·0	0·1
Lung	Rate	398·6	277·4	538·5	106·5	67·4	159·7	Mesothelioma	Number	4·4	3·4	5·3	3·7	3·1	4·5
Larynx	Rate	12·2	6·7	18·5	3·5	1·7	5·3	Lung	Rate	21·0	12·9	30·3	6·7	4·0	11·0
Mesothelioma	Rate	40·3	34·6	46·4	17·8	15·2	21·1	Ovary	Rate	8·2	3·8	14·6	3·2	1·4	5·8
								Larynx	Rate	0·2	0·1	0·4	0·0	0·0	0·1
								Mesothelioma	Rate	12·3	9·5	15·1	7·2	5·8	9·0
**Bolivia (Plurinational State of)**															
Lung	Number	294·1	134·3	578·4	1,705·7	811·2	3,168·0	Lung	Number	53·1	20·5	114·0	367·9	153·5	699·6
Larynx	Number	9·4	3·8	16·9	66·8	25·0	134·5	Ovary	Number	10·5	3·4	25·8	144·8	51·9	289·3
Mesothelioma	Number	88·1	53·8	140·2	507·9	292·0	736·2	Larynx	Number	1·7	0·6	4·3	7·0	2·3	16·1
Lung	Rate	23·5	11·0	45·2	44·4	21·4	80·8	Mesothelioma	Number	36·8	16·8	64·2	218·3	111·3	394·5
Larynx	Rate	0·8	0·3	1·4	1·8	0·7	3·5	Lung	Rate	3·5	1·4	7·2	7·3	3·1	13·7
Mesothelioma	Rate	5·6	3·5	8·9	11·1	6·3	16·1	Ovary	Rate	0·6	0·2	1·5	2·8	1·0	5·6
								Larynx	Rate	0·1	0·0	0·3	0·1	0·0	0·3
								Mesothelioma	Rate	2·1	1·0	3·7	4·0	2·1	7·2
**Brazil**															
Lung	Number	19,175·6	11,895·1	28,656·2	41,384·5	26,393·7	60,076·7	Lung	Number	3,459·0	2,332·4	4,737·0	16,039·3	10,304·6	22,035·8
Larynx	Number	1,375·9	709·5	2,122·7	3,142·1	1,650·3	4,969·7	Ovary	Number	1,301·8	613·9	2,045·8	4,229·1	2,016·4	6,636·7
Mesothelioma	Number	5,134·4	4,351·0	5,944·9	12,902·8	11,110·6	14,774·4	Larynx	Number	138·3	69·3	224·6	326·3	160·7	516·4
Lung	Rate	47·8	30·0	70·6	36·5	23·4	52·5	Mesothelioma	Number	3,239·8	2,637·0	3,817·5	8,251·6	6,811·5	9,949·3
Larynx	Rate	3·3	1·7	5·1	2·7	1·4	4·2	Lung	Rate	8·2	5·5	11·1	10·9	7·0	14·9
Mesothelioma	Rate	10·8	9·2	12·5	10·5	9·1	12·0	Ovary	Rate	2·9	1·4	4·6	2·9	1·4	4·5
								Larynx	Rate	0·3	0·2	0·5	0·2	0·1	0·3
								Mesothelioma	Rate	6·4	5·2	7·4	5·8	4·8	7·0
**Canada**															
Lung	Number	71,107·1	48,639·5	92,318·1	78,956·9	56,550·0	103,883·5	Ovary	Number	1,128·6	525·9	1,855·1	2,264·8	1,065·6	3,624·7
Larynx	Number	1,246·1	680·9	1,849·6	1,153·3	643·2	1,715·9	Lung	Number	4,468·0	2,775·1	6,437·5	12,974·9	8,335·7	18,463·7
Mesothelioma	Number	5,089·0	4,378·6	5,892·8	7,729·4	6,805·7	8,809·1	Larynx	Number	39·7	18·6	65·5	47·7	22·2	80·4
Lung	Rate	485·0	336·3	629·2	204·3	145·1	269·3	Mesothelioma	Number	891·4	737·7	1,065·3	1,909·0	1,596·4	2,242·3
Larynx	Rate	8·6	4·7	12·7	3·0	1·7	4·5	Ovary	Rate	5·9	2·8	9·8	5·1	2·4	8·2
Mesothelioma	Rate	35·1	30·3	40·6	20·9	18·3	23·8	Lung	Rate	23·5	14·6	33·8	28·6	18·4	40·9
								Larynx	Rate	0·2	0·1	0·3	0·1	0·0	0·2
**Chile**															
Lung	Number	3,377·9	2,036·9	4,914·8	5,619·0	3,459·6	8,245·0	Lung	Number	285·1	176·2	428·4	898·4	560·8	1,308·1
Larynx	Number	111·1	57·2	167·0	100·4	51·2	159·7	Ovary	Number	113·3	54·0	192·1	280·1	126·5	454·6
Mesothelioma	Number	505·8	436·3	592·7	1,154·7	1,004·1	1,326·5	Larynx	Number	4·8	2·2	8·1	5·8	2·7	10·0
Lung	Rate	78·7	47·9	113·7	45·9	28·3	67·2	Mesothelioma	Number	134·5	110·9	161·0	507·3	420·1	607·9
Larynx	Rate	2·6	1·3	3·9	0·8	0·4	1·3	Lung	Rate	5·1	3·2	7·7	5·7	3·6	8·3
Mesothelioma	Rate	10·9	9·5	12·7	9·5	8·2	10·9	Ovary	Rate	2·0	1·0	3·4	1·9	0·8	3·0
								Larynx	Rate	0·1	0·0	0·1	0·0	0·0	0·1
								Mesothelioma	Rate	2·4	2·0	2·8	3·5	2·9	4·2
**Colombia**															
Lung	Number	1,572·0	936·0	2,320·6	4,308·4	2,379·0	7,188·4	Lung	Number	317·3	202·9	449·9	893·8	547·0	1,353·3
Larynx	Number	89·9	44·7	144·4	185·1	88·3	310·1	Ovary	Number	117·6	52·6	196·0	416·4	184·7	732·8
Mesothelioma	Number	484·1	404·5	576·5	1,669·1	1,421·3	1,925·3	Larynx	Number	17·1	8·6	26·9	11·2	5·1	19·3

Lung	Rate	19·7	11·7	28·9	16·4	9·1	27·5	Mesothelioma	Number	267·4	218·2	315·8	744·6	598·2	910·7
Larynx	Rate	1·1	0·6	1·8	0·7	0·3	1·2	Lung	Rate	3·7	2·3	5·2	2·7	1·7	4·1
Mesothelioma	Rate	5·4	4·6	6·4	6·2	5·3	7·1	Ovary	Rate	1·3	0·6	2·2	1·3	0·6	2·2
								Larynx	Rate	0·2	0·1	0·3	0·0	0·0	0·1
								Mesothelioma	Rate	2·8	2·3	3·3	2·3	1·9	2·8
**Costa Rica**															
Lung	Number	109·5	62·3	171·4	244·3	135·3	389·6	Lung	Number	12·8	7·9	19·3	28·7	16·3	45·1
Larynx	Number	6·1	3·1	9·7	10·3	4·9	17·5	Ovary	Number	2·4	1·1	4·0	11·9	5·2	21·5
Mesothelioma	Number	19·0	15·4	22·5	78·3	63·8	93·1	Larynx	Number	0·5	0·2	0·8	0·3	0·1	0·6
Lung	Rate	14·6	8·4	22·6	9·2	5·1	14·5	Mesothelioma	Number	9·4	7·4	11·6	32·5	24·5	40·9
Larynx	Rate	0·8	0·4	1·3	0·4	0·2	0·7	Lung	Rate	1·5	0·9	2·3	0·9	0·5	1·4
Mesothelioma	Rate	2·3	1·9	2·7	2·6	2·1	3·1	Ovary	Rate	0·3	0·1	0·5	0·4	0·2	0·7
								Larynx	Rate	0·1	0·0	0·1	0·0	0·0	0·0
								Mesothelioma	Rate	1·1	0·8	1·3	1·0	0·8	1·3
**Cuba**															
Lung	Number	1,913·6	1,143·4	2,866·0	2,547·9	1,383·4	4,345·1	Lung	Number	207·3	130·6	304·5	451·7	256·9	698·4
Larynx	Number	86·4	44·4	135·0	206·7	94·2	371·4	Ovary	Number	45·6	20·6	82·0	76·2	32·0	134·2
Mesothelioma	Number	152·3	129·9	180·1	302·6	242·9	358·9	Larynx	Number	7·9	3·8	13·2	10·3	4·5	17·8
Lung	Rate	36·6	21·7	54·6	26·7	14·5	45·8	Mesothelioma	Number	132·1	105·4	164·0	125·6	102·4	158·4
Larynx	Rate	1·7	0·9	2·6	2·1	1·0	3·9	Lung	Rate	3·9	2·5	5·8	4·1	2·3	6·3
Mesothelioma	Rate	3·0	2·5	3·5	3·2	2·5	3·8	Ovary	Rate	0·9	0·4	1·6	0·7	0·3	1·3
								Larynx	Rate	0·2	0·1	0·3	0·1	0·0	0·2
								Mesothelioma	Rate	2·5	2·0	3·1	1·3	1·0	1·6
**Dominica**															
Lung	Number	15·1	7·7	24·3	30·5	15·6	53·4	Lung	Number	0·0	0·0	0·0	0·0	0·0	0·0
Larynx	Number	0·7	0·3	1·1	1·5	0·7	2·6	Ovary	Number	0·0	0·0	0·0	0·0	0·0	0·0
Mesothelioma	Number	2·2	1·6	3·0	5·3	3·9	6·9	Larynx	Number	0·0	0·0	0·0	0·0	0·0	0·0
Lung	Rate	51·7	26·2	83·4	62·1	32·0	108·4	Mesothelioma	Number	0·0	0·0	0·0	0·0	0·0	0·0
Larynx	Rate	2·3	1·1	4·0	3·0	1·5	5·3	Lung	Rate	0·0	0·0	0·0	0·0	0·0	0·0
Mesothelioma	Rate	8·3	6·1	11·6	11·2	8·2	14·5	Ovary	Rate	0·0	0·0	0·0	0·0	0·0	0·0
								Larynx	Rate	0·0	0·0	0·0	0·0	0·0	0·0
								Mesothelioma	Rate	0·0	0·0	0·0	0·0	0·0	0·0
**Dominican Republic**															
Lung	Number	139·2	73·4	252·0	635·9	310·6	1,180·8	Lung	Number	6·3	0·9	14·8	46·0	15·4	110·4
Larynx	Number	7·2	3·3	13·7	31·4	13·3	57·4	Ovary	Number	1·7	0·3	4·5	10·6	2·6	24·6
Mesothelioma	Number	50·7	29·4	78·9	143·0	89·8	210·6	Larynx	Number	0·3	0·1	0·7	1·1	0·3	2·6
Lung	Rate	8·3	4·4	15·1	15·2	7·4	28·2	Mesothelioma	Number	23·0	9·0	41·3	71·5	30·1	130·0
Larynx	Rate	0·4	0·2	0·8	0·7	0·3	1·3	Lung	Rate	0·3	0·0	0·8	0·9	0·3	2·1
Mesothelioma	Rate	2·7	1·6	4·1	2·9	1·9	4·2	Ovary	Rate	0·1	0·0	0·2	0·2	0·0	0·4
								Larynx	Rate	0·0	0·0	0·0	0·0	0·0	0·0
								Mesothelioma	Rate	1·1	0·4	1·9	1·3	0·5	2·3
**Ecuador**															
Lung	Number	200·8	119·8	306·3	605·1	339·3	938·7	Lung	Number	45·8	31·0	63·4	241·4	155·9	372·8
Larynx	Number	8·5	4·4	13·6	19·2	8·9	31·2	Ovary	Number	3·0	1·4	5·1	118·0	54·3	208·4
Mesothelioma	Number	84·1	70·5	101·5	259·2	210·4	309·2	Larynx	Number	1·7	0·8	2·6	2·4	1·1	4·0
Lung	Rate	8·8	5·2	13·4	7·5	4·2	11·6	Mesothelioma	Number	52·7	41·9	65·6	160·7	127·1	196·3
Larynx	Rate	0·4	0·2	0·6	0·2	0·1	0·4	Lung	Rate	2·0	1·3	2·7	2·6	1·7	4·0
Mesothelioma	Rate	3·2	2·8	3·9	3·1	2·5	3·6	Ovary	Rate	0·1	0·1	0·2	1·3	0·6	2·2

								Larynx	Rate	0·1	0·0	0·1	0·0	0·0	0·0
								Mesothelioma	Rate	1·9	1·6	2·4	1·7	1·3	2·0
**El Salvador**															
Lung	Number	3·0	0·3	7·8	38·6	18·3	72·3	Lung	Number	0·1	0·0	0·3	5·5	1·9	10·7
Larynx	Number	0·1	0·0	0·4	1·7	0·6	3·5	Ovary	Number	0·0	0·0	0·0	2·1	0·7	4·6
Mesothelioma	Number	7·5	3·5	11·9	29·6	15·9	47·0	Larynx	Number	0·0	0·0	0·0	0·1	0·0	0·2
Lung	Rate	0·2	0·0	0·6	1·6	0·7	2·9	Mesothelioma	Number	4·0	1·7	6·9	19·2	8·3	31·3
Larynx	Rate	0·0	0·0	0·0	0·1	0·0	0·1	Lung	Rate	0·0	0·0	0·0	0·1	0·1	0·3
Mesothelioma	Rate	0·6	0·3	0·9	1·2	0·6	1·9	Ovary	Rate	0·0	0·0	0·0	0·1	0·0	0·1
								Larynx	Rate	0·0	0·0	0·0	0·0	0·0	0·0
								Mesothelioma	Rate	0·3	0·1	0·4	0·5	0·2	0·9
**Greenland**															
Lung	Number	11·2	5·7	20·5	166·2	89·6	252·3	Lung	Number	24·5	10·6	48·7	41·1	18·0	68·0
Larynx	Number	0·1	0·0	0·2	2·3	1·0	4·2	Ovary	Number	3·2	1·3	6·4	5·0	2·1	9·3
Mesothelioma	Number	0·5	0·3	0·7	8·6	6·1	11·0	Larynx	Number	0·1	0·0	0·2	0·1	0·0	0·1
Lung	Rate	116·9	58·2	214·7	549·8	307·6	801·5	Mesothelioma	Number	0·6	0·3	1·2	2·6	1·3	4·4
Larynx	Rate	1·0	0·4	2·0	7·8	3·6	14·2	Lung	Rate	153·0	67·3	309·7	129·5	58·5	217·3
Mesothelioma	Rate	4·1	2·9	5·7	29·3	20·9	38·1	Ovary	Rate	19·6	7·9	39·1	15·0	6·1	28·3
								Larynx	Rate	0·4	0·1	0·9	0·2	0·1	0·4
								Mesothelioma	Rate	3·7	1·7	7·3	8·2	4·2	14·0
**Grenada**															
Lung	Number	18·8	11·7	28·0	16·7	9·7	24·7	Lung	Number	2·0	1·2	2·9	3·3	2·0	4·9
Larynx	Number	0·8	0·4	1·2	1·1	0·5	1·9	Ovary	Number	1·3	0·6	2·3	3·0	1·3	5·2
Mesothelioma	Number	2·5	2·1	3·0	4·3	3·6	5·4	Larynx	Number	0·1	0·0	0·1	0·1	0·0	0·1
Lung	Rate	58·6	36·8	87·0	21·4	12·5	31·4	Mesothelioma	Number	3·2	2·5	3·8	3·4	2·7	4·1
Larynx	Rate	2·5	1·2	3·8	1·4	0·7	2·4	Lung	Rate	4·5	2·8	6·7	4·1	2·5	6·1
Mesothelioma	Rate	7·9	6·6	9·5	5·6	4·6	7·0	Ovary	Rate	3·2	1·5	5·6	3·9	1·7	6·7
								Larynx	Rate	0·2	0·1	0·3	0·1	0·0	0·2
								Mesothelioma	Rate	8·2	6·3	10·0	4·9	3·9	6·1
**Guatemala**															
Lung	Number	61·0	32·7	96·7	157·6	81·4	268·9	Lung	Number	15·0	8·0	24·3	41·6	23·3	69·8
Mesothelioma	Number	41·9	32·0	51·7	95·8	71·8	121·0	Mesothelioma	Number	20·3	15·2	25·9	60·3	43·6	82·4
Larynx	Number	3·9	1·9	6·8	5·2	2·5	8·6	Larynx	Number	0·8	0·4	1·5	0·7	0·3	1·3
Lung	Rate	4·3	2·4	6·7	3·4	1·8	5·6	Ovary	Number	2·1	0·9	3·8	17·2	7·7	32·8
Mesothelioma	Rate	2·3	1·8	2·8	1·8	1·3	2·2	Lung	Rate	0·9	0·5	1·5	0·7	0·4	1·2
Larynx	Rate	0·3	0·1	0·5	0·1	0·1	0·2	Mesothelioma	Rate	1·1	0·8	1·3	0·9	0·7	1·3
								Larynx	Rate	0·1	0·0	0·1	0·0	0·0	0·0
								Ovary	Rate	0·1	0·1	0·2	0·3	0·1	0·5
**Guyana**															
Lung	Number	25·1	13·7	39·1	55·7	30·0	91·4	Lung	Number	3·2	2·0	4·8	12·7	7·2	20·6
Mesothelioma	Number	10·0	8·2	12·2	26·6	20·9	33·0	Ovary	Number	3·8	1·5	6·7	15·2	7·0	28·3
Larynx	Number	1·5	0·7	2·3	3·9	1·7	6·7	Mesothelioma	Number	11·0	8·2	14·1	34·0	23·8	48·3
Lung	Rate	14·5	8·1	22·6	15·9	8·7	25·7	Larynx	Number	0·1	0·0	0·1	0·1	0·1	0·3
Mesothelioma	Rate	5·1	4·2	6·1	7·0	5·5	8·6	Lung	Rate	1·7	1·0	2·5	3·2	1·9	5·2
Larynx	Rate	0·8	0·4	1·3	1·1	0·5	1·9	Ovary	Rate	1·8	0·8	3·3	3·7	1·7	6·9
								Mesothelioma	Rate	4·5	3·4	5·7	8·2	5·8	11·6
								Larynx	Rate	0·0	0·0	0·1	0·0	0·0	0·1
**Haiti**															

Lung	Number	306·1	143·2	592·0	919·9	443·2	1,680·7	Lung	Number	25·6	8·4	68·8	93·1	28·2	201·8
Larynx	Number	21·9	8·8	41·5	76·1	31·6	156·9	Ovary	Number	15·7	3·8	42·9	75·5	18·3	189·4
Mesothelioma	Number	83·9	49·5	132·1	302·9	176·4	431·6	Larynx	Number	1·1	0·3	2·7	3·1	0·8	7·4
Lung	Rate	21·0	9·6	40·8	29·8	14·6	54·7	Mesothelioma	Number	47·5	18·5	97·8	172·0	66·9	321·5
Larynx	Rate	1·5	0·6	2·7	2·4	0·9	4·7	Lung	Rate	1·5	0·5	3·8	2·2	0·7	4·7
Mesothelioma	Rate	4·7	2·8	7·4	7·4	4·4	10·9	Ovary	Rate	0·9	0·2	2·3	1·6	0·4	4·0
								Larynx	Rate	0·1	0·0	0·2	0·1	0·0	0·2
								Mesothelioma	Rate	2·3	0·9	4·4	3·1	1·3	5·6
**Honduras**															
Lung	Number	107·1	45·2	197·6	584·2	241·4	982·6	Lung	Number	23·4	6·8	43·3	221·0	70·9	432·8
Larynx	Number	4·2	1·6	8·0	23·8	9·3	46·0	Ovary	Number	6·3	1·9	12·9	73·8	17·4	149·0
Mesothelioma	Number	36·5	22·9	57·0	156·0	89·3	226·4	Larynx	Number	0·6	0·2	1·1	4·3	1·4	9·5
Lung	Rate	11·8	5·2	21·4	19·0	8·0	31·9	Mesothelioma	Number	17·3	5·6	31·0	98·0	35·2	157·9
Larynx	Rate	0·5	0·2	0·9	0·8	0·3	1·5	Lung	Rate	2·5	0·8	4·5	6·4	2·2	12·4
Mesothelioma	Rate	3·6	2·2	5·6	4·6	2·7	6·6	Ovary	Rate	0·6	0·2	1·3	2·0	0·5	4·1
								Larynx	Rate	0·1	0·0	0·1	0·1	0·0	0·3
								Mesothelioma	Rate	1·6	0·5	2·8	2·5	0·9	4·0
**Jamaica**															
Lung	Number	346·6	211·4	528·0	477·1	261·9	800·1	Lung	Number	16·2	9·9	24·1	14·1	7·8	22·4
Mesothelioma	Number	43·8	36·1	52·2	93·2	74·0	117·0	Mesothelioma	Number	31·1	24·8	39·2	42·3	31·9	54·0
Larynx	Number	8·0	4·1	12·9	15·7	7·1	27·7	Larynx	Number	0·3	0·1	0·5	0·2	0·1	0·3
Lung	Rate	41·5	25·3	63·0	31·6	17·5	53·0	Ovary	Number	8·5	4·1	14·8	6·5	2·9	12·3
Mesothelioma	Rate	5·5	4·5	6·6	6·1	4·9	7·6	Lung	Rate	1·5	0·9	2·3	0·8	0·4	1·3
Larynx	Rate	0·9	0·5	1·5	1·0	0·5	1·8	Mesothelioma	Rate	3·7	2·9	4·6	2·6	2·0	3·4
								Larynx	Rate	0·0	0·0	0·0	0·4	0·2	0·7
								Ovary	Rate	0·8	0·4	1·5	0·0	0·0	0·0
**Mexico**															
Lung	Number	6,466·3	3,964·7	9,503·2	10,116·2	5,897·6	15,157·6	Lung	Number	993·1	672·4	1,381·4	2,116·2	1,294·6	3,037·5
Larynx	Number	335·1	175·3	518·0	441·4	220·9	694·6	Ovary	Number	387·8	191·6	618·2	1,786·9	848·0	3,019·1
Mesothelioma	Number	1,605·0	1,380·6	1,865·5	4,815·0	4,170·5	5,540·0	Larynx	Number	32·8	16·2	52·4	29·0	13·9	50·0
Lung	Rate	34·2	21·0	50·1	16·4	9·6	24·5	Mesothelioma	Number	800·8	655·2	944·3	2,410·1	1,967·4	2,909·0
Larynx	Rate	1·8	0·9	2·7	0·7	0·4	1·1	Lung	Rate	4·7	3·2	6·6	2·9	1·8	4·2
Mesothelioma	Rate	7·4	6·4	8·6	7·3	6·3	8·4	Ovary	Rate	1·8	0·9	2·8	2·4	1·1	4·0
								Larynx	Rate	0·2	0·1	0·3	0·0	0·0	0·1
								Mesothelioma	Rate	3·4	2·8	3·9	3·2	2·6	3·8
**Nicaragua**															
Lung	Number	15·7	6·9	27·8	57·6	27·5	102·1	Lung	Number	1·2	0·4	2·9	17·1	5·6	29·5
Larynx	Number	1·1	0·5	2·3	4·6	1·9	9·1	Ovary	Number	0·3	0·1	0·8	8·4	2·3	16·7
Mesothelioma	Number	13·6	8·8	19·2	64·9	45·6	85·0	Larynx	Number	0·1	0·0	0·2	0·4	0·1	0·9
Lung	Rate	2·5	1·1	4·2	2·8	1·3	5·0	Mesothelioma	Number	7·5	3·7	13·2	36·4	18·9	60·1
Larynx	Rate	0·2	0·1	0·4	0·2	0·1	0·4	Lung	Rate	0·2	0·1	0·4	0·6	0·2	1·1
Mesothelioma	Rate	1·9	1·2	2·6	2·8	1·9	3·6	Ovary	Rate	0·0	0·0	0·1	0·3	0·1	0·6
								Larynx	Rate	0·0	0·0	0·0	0·0	0·0	0·0
								Mesothelioma	Rate	0·9	0·5	1·6	1·3	0·7	2·1
**Panama**															
Lung	Number	62·5	34·7	101·6	121·9	66·1	205·0	Lung	Number	9·8	6·1	14·1	19·7	10·7	32·8
Larynx	Number	2·9	1·5	4·7	5·0	2·4	8·9	Ovary	Number	2·0	0·9	3·4	9·0	3·9	17·7
Mesothelioma	Number	12·7	10·4	15·3	38·0	30·1	45·9	Larynx	Number	0·3	0·2	0·6	0·2	0·1	0·4

Lung	Rate	9·1	5·1	14·8	5·7	3·1	9·6	Mesothelioma	Number	10·1	7·8	12·3	30·9	23·9	38·4
Larynx	Rate	0·4	0·2	0·7	0·2	0·1	0·4	Lung	Rate	1·4	0·9	2·0	0·8	0·4	1·3
Mesothelioma	Rate	1·7	1·4	2·1	1·7	1·4	2·1	Ovary	Rate	0·3	0·1	0·5	0·4	0·2	0·7
								Larynx	Rate	0·1	0·0	0·1	0·0	0·0	0·0
								Mesothelioma	Rate	1·3	1·1	1·6	1·3	1·0	1·6
**Paraguay**															
Lung	Number	147·3	81·3	235·4	1,069·3	471·6	1,781·8	Lung	Number	20·5	9·7	39·2	169·3	71·3	301·2
Larynx	Number	6·5	2·9	11·3	39·5	16·4	70·9	Ovary	Number	7·5	2·8	14·5	65·5	23·8	125·1
Mesothelioma	Number	51·5	36·8	70·1	223·0	160·1	292·0	Larynx	Number	0·6	0·2	1·1	3·1	1·1	6·0
Lung	Rate	15·3	8·5	24·5	46·1	20·7	75·5	Mesothelioma	Number	34·7	18·6	60·6	139·9	81·8	224·5
Larynx	Rate	0·7	0·3	1·1	1·6	0·7	2·9	Lung	Rate	1·9	0·9	3·6	5·9	2·5	10·5
Mesothelioma	Rate	4·6	3·3	6·3	8·3	5·9	10·7	Ovary	Rate	0·7	0·3	1·3	2·3	0·8	4·3
								Larynx	Rate	0·1	0·0	0·1	0·1	0·0	0·2
								Mesothelioma	Rate	2·9	1·6	5·0	4·6	2·7	7·4
**Peru**															
Lung	Number	1,543·8	744·3	2,524·3	2,371·0	1,168·0	4,008·3	Lung	Number	326·0	119·8	597·5	853·3	329·3	1,647·8
Larynx	Number	44·8	18·2	77·4	79·3	29·7	142·3	Ovary	Number	71·8	25·8	139·7	315·7	102·3	710·2
Mesothelioma	Number	368·0	229·4	562·0	1,072·3	667·8	1,503·7	Larynx	Number	5·9	1·9	12·2	8·5	2·8	20·4
Lung	Rate	30·2	15·1	49·2	14·3	7·2	24·1	Mesothelioma	Number	208·8	105·6	338·1	720·8	380·7	1,200·0
Larynx	Rate	0·9	0·4	1·6	0·5	0·2	0·9	Lung	Rate	5·7	2·1	10·3	4·4	1·7	8·5
Mesothelioma	Rate	6·2	3·9	9·3	6·0	3·7	8·4	Ovary	Rate	1·2	0·4	2·3	1·6	0·5	3·7
								Larynx	Rate	0·1	0·0	0·2	0·0	0·0	0·1
								Mesothelioma	Rate	3·3	1·7	5·3	3·7	2·0	6·2
**Puerto Rico**															
Lung	Number	731·6	443·8	1,129·9	767·2	428·7	1,243·7	Lung	Number	27·3	16·1	40·9	47·1	28·1	76·0
Larynx	Number	40·0	19·6	63·9	27·8	13·7	46·8	Ovary	Number	9·2	4·0	16·6	24·6	10·7	48·4
Mesothelioma	Number	170·8	146·0	200·2	166·1	138·7	193·6	Larynx	Number	0·6	0·3	1·0	0·5	0·2	0·8
Lung	Rate	42·1	25·4	65·3	18·7	10·2	30·5	Mesothelioma	Number	21·2	15·8	27·0	52·9	43·0	64·3
Larynx	Rate	2·3	1·1	3·7	0·7	0·3	1·2	Lung	Rate	1·4	0·8	2·0	1·0	0·6	1·7
Mesothelioma	Rate	10·1	8·7	11·9	5·3	4·3	6·3	Ovary	Rate	0·5	0·2	0·8	0·6	0·3	1·2
								Larynx	Rate	0·0	0·0	0·0	0·0	0·0	0·0
								Mesothelioma	Rate	1·1	0·8	1·4	1·7	1·4	2·1
**Saint Kitts and Nevis**															
Lung	Number	14·3	9·4	20·3	13·4	8·4	19·6	Lung	Number	0·3	0·2	0·5	0·4	0·3	0·7
Mesothelioma	Number	4·1	3·5	4·7	7·6	6·5	9·0	Ovary	Number	0·3	0·1	0·5	0·4	0·2	0·8
Larynx	Number	1·1	0·6	1·9	1·3	0·7	2·1	Mesothelioma	Number	0·3	0·2	0·4	0·4	0·3	0·6
Lung	Rate	84·7	56·1	121·6	50·0	32·5	72·0	Larynx	Number	0·0	0·0	0·0	0·0	0·0	0·0
Mesothelioma	Rate	27·4	23·7	32·2	26·4	22·6	31·2	Lung	Rate	1·6	1·0	2·6	1·4	0·9	2·3
Larynx	Rate	6·8	3·6	11·5	4·9	2·5	7·8	Ovary	Rate	1·4	0·6	2·4	1·4	0·6	2·6
								Mesothelioma	Rate	2·0	1·6	2·5	1·4	1·0	1·8
								Larynx	Rate	0·0	0·0	0·0	0·0	0·0	0·0
**Saint Lucia**															
Lung	Number	9·6	5·6	15·0	9·7	5·3	16·4	Lung	Number	2·1	1·3	2·9	2·8	1·7	4·5
Larynx	Number	0·6	0·3	0·9	0·9	0·4	1·4	Ovary	Number	1·5	0·7	2·7	2·7	1·2	4·7
Mesothelioma	Number	1·9	1·6	2·3	2·5	1·9	3·1	Larynx	Number	0·1	0·0	0·1	0·0	0·0	0·1
Lung	Rate	24·3	14·2	38·2	8·1	4·5	13·6	Mesothelioma	Number	3·2	2·6	3·8	2·9	2·3	3·6
Larynx	Rate	1·5	0·7	2·4	0·7	0·3	1·1	Lung	Rate	4·2	2·6	6·0	2·1	1·3	3·5
Mesothelioma	Rate	4·8	3·9	5·8	2·0	1·5	2·5	Ovary	Rate	3·1	1·4	5·6	2·1	0·9	3·6

								Larynx	Rate	0·1	0·1	0·2	0·0	0·0	0·1
								Mesothelioma	Rate	6·6	5·2	7·8	2·3	1·9	3·0
**Saint Vincent and the Grenadines**															
Lung	Number	6·2	3·5	9·4	8·5	4·9	13·8	Lung	Number	1·1	0·7	1·6	1·9	1·2	3·0
Larynx	Number	0·5	0·3	0·9	1·2	0·6	2·0	Larynx	Number	0·0	0·0	0·1	0·0	0·0	0·1
Mesothelioma	Number	1·7	1·4	2·0	3·2	2·6	3·9	Ovary	Number	0·9	0·4	1·5	1·6	0·7	3·0
Lung	Rate	19·7	11·3	29·8	10·2	5·9	16·6	Mesothelioma	Number	6·9	5·5	8·3	6·5	5·2	7·9
Larynx	Rate	1·7	0·9	3·0	1·5	0·7	2·4	Lung	Rate	2·7	1·7	4·0	2·4	1·5	3·9
Mesothelioma	Rate	5·3	4·4	6·3	4·0	3·2	4·9	Larynx	Rate	0·1	0·0	0·1	0·0	0·0	0·1
								Ovary	Rate	2·3	1·0	4·0	2·2	1·0	4·0
								Mesothelioma	Rate	18·1	14·6	21·4	9·2	7·4	11·4
**Suriname**															
Lung	Number	11·1	5·4	19·3	37·7	15·8	66·3	Lung	Number	2·3	1·0	4·6	11·0	4·4	20·6
Mesothelioma	Number	2·2	1·3	3·4	9·0	5·9	13·2	Ovary	Number	1·5	0·5	3·0	6·5	2·2	13·1
Larynx	Number	0·3	0·1	0·4	1·0	0·4	1·8	Mesothelioma	Number	3·0	1·4	5·1	12·5	5·7	21·0
Lung	Rate	9·6	4·6	16·9	13·1	5·6	22·2	Larynx	Number	0·0	0·0	0·1	0·1	0·0	0·2
Mesothelioma	Rate	1·7	1·0	2·6	2·8	1·8	4·1	Lung	Rate	1·7	0·8	3·5	3·0	1·2	5·7
Larynx	Rate	0·2	0·1	0·4	0·3	0·1	0·6	Ovary	Rate	1·1	0·4	2·2	1·8	0·6	3·5
								Mesothelioma	Rate	2·0	0·9	3·5	3·5	1·6	6·0
								Larynx	Rate	0·0	0·0	0·1	0·0	0·0	0·1
**Trinidad and Tobago**															
Lung	Number	94·6	54·5	143·6	154·5	77·1	272·0	Lung	Number	7·1	4·4	10·6	15·0	8·3	23·7
Larynx	Number	4·0	1·9	6·4	7·3	3·3	12·9	Ovary	Number	9·5	4·5	17·5	20·0	8·9	37·3
Mesothelioma	Number	24·5	20·2	29·1	51·5	42·4	63·4	Larynx	Number	0·2	0·1	0·3	0·3	0·1	0·5
Lung	Rate	27·4	16·0	41·6	17·7	9·2	30·7	Mesothelioma	Number	9·0	7·2	10·9	21·4	16·7	26·9
Larynx	Rate	1·1	0·5	1·8	0·8	0·4	1·4	Lung	Rate	1·7	1·1	2·6	1·5	0·8	2·4
Mesothelioma	Rate	6·3	5·2	7·5	5·7	4·7	6·9	Ovary	Rate	2·2	1·1	4·1	2·0	0·9	3·8
								Larynx	Rate	0·0	0·0	0·1	0·0	0·0	0·0
								Mesothelioma	Rate	2·0	1·6	2·4	2·3	1·7	2·8
**United States of America**															
Lung	Number	528,947·8	368,163·2	705,347·5	385,214·9	271,928·1	524,538·8	Lung	Number	50,426·4	34,335·7	66,363·2	69,781·7	44,390·6	94,200·3
Larynx	Number	6,911·2	3,823·4	10,293·3	6,717·5	3,651·2	10,132·0	Larynx	Number	364·2	181·1	585·9	397·9	202·4	641·4
Mesothelioma	Number	31,256·4	27,295·6	35,772·4	31,298·0	27,378·1	35,669·4	Mesothelioma	Number	8,130·1	6,637·1	9,696·7	12,016·1	9,914·2	14,352·4
Lung	Rate	377·3	262·9	504·0	131·3	93·0	179·0	Ovary	Number	11,573·4	5,554·8	17,766·3	12,989·1	6,135·6	20,176·4
Larynx	Rate	5·0	2·7	7·4	2·3	1·3	3·5	Lung	Rate	26·1	17·9	34·6	19·5	12·4	26·4
Mesothelioma	Rate	22·8	20·0	26·2	11·0	9·6	12·6	Larynx	Rate	0·2	0·1	0·3	0·1	0·1	0·2
								Mesothelioma	Rate	4·6	3·7	5·4	3·7	3·1	4·5
								Ovary	Rate	5·9	2·8	9·1	3·7	1·7	5·8
**United States Virgin Islands**															
Lung	Number	26·3	15·8	40·3	57·3	36·0	90·9	Lung	Number	2·6	1·3	4·0	6·0	2·8	10·0
Larynx	Number	1·1	0·5	1·9	2·3	1·0	3·7	Ovary	Number	0·9	0·3	1·6	4·1	1·7	7·8
Mesothelioma	Number	8·3	6·9	9·9	11·5	9·5	13·4	Larynx	Number	0·1	0·0	0·1	0·0	0·0	0·1
Lung	Rate	84·3	52·6	124·4	62·5	39·0	100·0	Mesothelioma	Number	2·2	1·8	2·8	3·8	3·0	4·9
Larynx	Rate	3·5	1·7	6·1	2·5	1·2	4·0	Lung	Rate	6·6	3·5	10·4	5·7	2·6	9·8
Mesothelioma	Rate	19·8	16·6	23·3	16·8	13·5	20·2	Ovary	Rate	2·1	0·9	3·7	4·4	1·8	8·2
								Larynx	Rate	0·1	0·0	0·3	0·0	0·0	0·1
								Mesothelioma	Rate	4·3	3·4	5·3	5·6	4·2	7·1

**Uruguay**															
Lung	Number	2,342·8	1,453·2	3,534·5	2,895·1	1,802·3	4,252·7	Lung	Number	54·9	35·9	81·6	272·1	164·6	386·2
Mesothelioma	Number	132·0	114·2	156·0	254·8	217·9	294·6	Mesothelioma	Number	31·7	25·9	37·8	112·6	92·7	135·0
Larynx	Number	95·2	48·4	151·0	91·4	47·2	147·0	Larynx	Number	1·5	0·7	2·5	3·2	1·4	5·6
Lung	Rate	136·2	84·9	205·0	121·0	75·0	177·9	Ovary	Number	33·8	15·3	56·3	85·4	41·3	141·7
Mesothelioma	Rate	7·7	6·7	9·1	11·3	9·7	13·1	Lung	Rate	2·3	1·5	3·5	8·0	4·9	11·3
Larynx	Rate	5·5	2·8	8·8	3·9	2·0	6·2	Mesothelioma	Rate	1·5	1·2	1·8	4·0	3·3	4·8
								Larynx	Rate	0·1	0·0	0·1	0·1	0·0	0·2
								Ovary	Rate	1·4	0·7	2·4	2·5	1·2	4·1
**Venezuela (Bolivarian Republic of)**															
Lung	Number	694·3	395·3	1,090·2	1,955·3	916·5	3,575·6	Lung	Number	186·6	122·5	278·9	413·8	229·8	731·4
Larynx	Number	37·7	18·2	60·7	86·7	36·8	159·0	Larynx	Number	6·9	3·4	11·6	7·7	3·2	15·0
Mesothelioma	Number	156·9	133·5	185·5	526·6	406·6	661·4	Mesothelioma	Number	115·9	93·0	142·3	307·7	230·9	383·2
Lung	Rate	16·9	9·7	26·2	14·2	7·0	25·8	Ovary	Number	15·3	7·5	26·5	151·9	70·7	279·7
Larynx	Rate	1·0	0·5	1·5	0·6	0·3	1·1	Lung	Rate	4·0	2·7	6·0	2·4	1·4	4·3
Mesothelioma	Rate	3·5	3·0	4·1	3·5	2·7	4·3	Larynx	Rate	0·2	0·1	0·3	0·0	0·0	0·1
								Mesothelioma	Rate	2·2	1·8	2·7	1·8	1·3	2·2
								Ovary	Rate	0·3	0·2	0·6	0·9	0·4	1·6

Table S4. Disability-adjusted life years (DALY) numbers and rates for cancer attributable to occupational asbestos exposure, by sex, cancer type and country, in 1990 and 2023 in the Americas.

Number: number of DALYs for all ages; Rate: age-standardized DALY rates per 100,000; 95%UI: 95% uncertainty interval.

Elaborated by the authors (2025).

Regions/Years	Mortality							DALY							
	APC (%)	95%UI		AAPC (%) 1990-2023	95%UI		Trend 1990-2023		APC (%)	95%UI		AAPC (%) 1990-2023	95%UI		Trend 1990-2023
**High-income North America**															
**All cancers**				**-0·3***	-0·4	-0·3	**Downward**					**-0·8***	-0·8	-0·7	**Downward**
1990-1995	2·9*	2·5	3·6					1990-1994	2·6*	1·8	3·9				
1995-2004	0·9*	0·6	1·1					1994-1999	0·9	-0·2	1·6				
2004-2013	-0·9*	-1·1	-0·6					1999-2005	-0·3	-2·3	0·2				
2013-2020	-2·9*	-3·5	-2·6					2005-2013	-1·6*	-2·4	-0·8				
2020-2023	-1·2	-2·1	0·3					2013-2020	-3·1*	-4·3	-2·7				
-	-	-	-					2020-2023	-1·0	-2·2	0·5				
**Ovarian**				**-1·0***	-1·1	-0·9	**Downward**					**-1·3***	-1·4	-1·3	**Downward**
1990-2004	0·5*	0·3	0·7					1990-2004	0·1	-0·2	0·4				
2004-2013	-1·8	-2·1	0·5					2004-2021	-2·5	-3·0	0·4				
2013-2020	-2·8*	-4·0	-1·6					2021-2023	-0·6	-2·5	0·4				
2020-2023	-1·1	-2·5	0·5					-	-	-	-				
**Lung**				**-0·2***	-0·2	-0·1	**Downward**					**-0·7***	-0·8	-0·6	**Downward**
1990-1995	3·7*	3·2	4·5					1990-1995	3·1*	2·6	3·9				
1995-2004	1·1*	0·8	1·4					1995-2003	0·6*	0·3	1·0				
2004-2013	-0·8*	-1·1	-0·0					2003-2013	-1·5*	-1·7	-1·2				
2013-2020	-3·1*	-3·8	-0·9					2013-2020	-3·5*	-4·2	-3·2				
2020-2023	-1·4	-2·4	0·1					2020-2023	-1·1	-2·2	0·5				
**Mesothelioma**				**-0·3***	-0·5	-0·1	**Downward**					**-0·5***	-0·6	-0·3	**Downward**
1990-2012	-0·1	-0·3	1·3					1990-1996	0·8	-0·2	3·4				
2012-2023	-0·8*	-3·1	-0·4					1996-2023	-0·8*	-0·9	-0·7				
**Central Latin America**															
**All cancers**				**-0·5***	-0·6	-0·5	**Downward**					**-0·5***	-0·5	-0·4	**Downward**
1990-1996	0·7*	0·1	1·4					1990-1996	0·5*	0·0	1·2				

1996-2002	-2·2*	-3·5	-1·6					1996-2002	-1·8*	-2·9	-1·3				
2002-2007	3·5*	2·5	4·9					2002-2007	3·5*	2·6	4·5				
2007-2023	-1·6*	-1·7	-1·5					2007-2023	-1·5*	-1·6	-1·4				
**Ovarian**				**0·7***	0·7	0·8	**Upward**					**0·9***	0·8	1·0	**Upward**
1990-1993	-0·6	-2·8	0·4					1990-1993	-0·3	-2·6	0·7				
1993-1996	4·7*	3·2	5·5					1993-1996	4·6*	3·1	5·4				
1996-2001	-1·4*	-2·2	-0·9					1996-2001	-1·1*	-2·2	-0·6				
2001-2007	3·9*	3·3	4·5					2001-2007	3·8*	3·2	4·5				
2007-2023	-0·2*	-0·3	-0·1					2007-2023	0·0	-0·1	0·1				
**Lung**				**-1·1***	-1·2	-1·1	**Downward**					**-1·2***	-1·2	-1·1	**Downward**
1990-1996	0·6*	0·1	1·2					1990-1996	0·2	-0·3	0·8				
1996-2001	-4·0*	-5·0	-3·3					1996-2001	-3·6*	-4·6	-2·9				
2001-2007	1·7*	1·1	2·5					2001-2007	1·4*	0·8	2·1				
2007-2023	-1·9*	-2·0	-1·8					2007-2023	-1·9*	-2·0	-1·8				
**Mesothelioma**				**-0·1**	-0·3	0·0	**Stable**					**-0·3***	-0·4	-0·2	**Downward**
1990-2004	-0·1	-0·4	0·1					1990-2004	-0·1	-0·4	0·2				
2004-2007	8·9*	5·0	9·9					2004-2007	8·2*	4·5	9·4				
2007-2020	-2·3*	-2·9	-2·0					2007-2023	-2·1*	-2·4	-1·9				
2020-2023	0·8	-1·7	4·5					-	-	-	-				
**Caribbean**															
**All cancers**				**0·0**	-0·2	0·2	**Stable**					**-0·1**	-0·3	0·1	**Stable**
1990-2023	0·0	-0·2	0·2					1990-2023	-0·1	-0·3	0·1				
**Ovarian**				**0·2***	0·1	0·4	**Upward**					**0·3***	0·1	0·4	**Upward**
1990-1993	-2·6*	-6·3	-0·3					1990-1993	-3·1*	-6·6	-0·7				
1993-2000	2·0*	1·4	4·2					1993-2000	2·0*	1·5	4·0				
2000-2003	-4·0*	-5·4	-1·6					2000-2003	-3·9*	-5·3	-1·7				
2003-2006	2·9*	0·7	4·4					2003-2006	2·7*	0·6	4·3				
2006-2013	-1·5*	-3·8	-0·9					2006-2013	-1·4*	-3·7	-0·8				

2013-2023	1·5*	1·1	2·1					2013-2023	1·8*	1·3	2·3				
**Lung**				**0·2***	0·1	0·4	**Upward**					**0·1***	0·0	0·3	**Upward**
1990-2002	0·1	-0·8	0·6					1990-2003	0·5	-0·3	0·9				
2002-2009	3·0*	1·9	6·2					2003-2009	2·9*	1·7	5·7				
2009-2012	-5·2*	-6·7	-1·7					2009-2012	-5·9*	-7·4	-2·7				
2012-2023	0·2	-0·3	1·3					2012-2023	-0·0	-0·5	0·7				
**Mesothelioma**				**-0·5***	-0·8	-0·2	**Downward**					**-0·3**	-0·8	0·1	**Stable**
1990-2023	-0·5*	-0·8	-0·2					1990-2013	-1·0*	-2·9	-0·6				
-	-	-	-					2013-2023	1·2	-0·2	8·3				
**Andean Latin America**															
**All cancers**				**0·6***	0·4	0·8	**Upward**					**0·6***	0·4	0·8	**Upward**
1990-1998	5·7*	4·9	6·7					1990-1998	5·9*	4·9	7·0				
1998-2001	-12·5*	-14·2	-8·4					1998-2001	-12·9*	-14·9	-8·4				
2001-2004	5·3*	2·0	7·4					2001-2004	4·7*	1·1	7·0				
2004-2015	-2·0*	-2·9	-1·6					2004-2015	-2·2*	-3·2	-1·7				
2015-2023	2·8*	1·9	4·0					2015-2023	3·3*	2·3	4·6				
**Ovarian**				**2·5***	2·3	2·6	**Upward**					**2·4***	2·3	2·6	**Upward**
1990-1997	9·9*	9·1	11·0					1990-1997	9·9*	8·9	11·1				
1997-2001	-3·2*	-5·7	-1·0					1997-2001	-3·9*	-6·8	-1·5				
2001-2004	6·2*	3·0	7·9					2001-2004	6·1*	2·6	8·0				
2004-2014	-1·3*	-2·3	-0·9					2004-2015	-1·3*	-2·2	-0·8				
2014-2023	2·6*	1·9	3·5					2015-2021	3·3*	2·4	4·5				
-	-	-	-					2021-2023	-7·2*	-11·9	-1·5				
**Lung**				**0·2***	0·1	0·4	**Upward**					**0·1**	-0·0	0·3	**Stable**
1990-1996	7·7*	6·6	8·9					1990-1996	7·6*	6·5	8·9				
1996-2001	-8·5*	-10·3	-7·3					1996-2001	-9·0*	-10·9	-7·8				
2001-2004	5·9*	2·3	7·6					2001-2004	5·6*	1·9	7·4				
2004-2015	-2·2*	-3·0	-1·8					2004-2015	-2·3*	-3·2	-1·9				

2015-2023	1·9*	1·1	3·1					2015-2023	2·2*	1·3	3·4				
**Mesothelioma**				**0·9***	0·2	1·6	**Upward**					**0·9***	0·2	1·6	**Upward**
1990-1999	7·1*	2·6	11·6					1990-1999	7·6*	2·5	12·7				
1999-2002	-14·4	-18·0	10·4					1999-2002	-15·2	-19·0	11·7				
2002-2015	-1·4	-11·3	0·8					2002-2015	-1·5	-12·0	0·8				
2015-2023	4·1*	0·6	14·9					2015-2023	4·3*	0·5	15·7				
**Tropical Latin America**															
**All cancers**				**0·5***	0·4	0·5	**Upward**					**0·4***	0·3	0·5	**Upward**
1990-1997	-0·4	-1·0	0·1					1990-1997	-0·0	-1·2	0·4				
1997-2012	1·1*	0·9	1·3					1997-2013	1·0*	0·8	1·4				
2012-2023	0·2	-0·1	0·4					2013-2023	-0·1	-0·4	0·1				
**Ovarian**				**-0·1**	-0·1	0·0	**Stable**					**-0·04**	-0·1	0·03	**Stable**
1990-2009	0·3*	0·2	0·5					1990-2010	0·2*	0·1	0·4				
2009-2023	-0·5*	-0·8	-0·3					2010-2023	-0·4*	-0·7	-0·2				
**Lung**				**1·0***	0·9	1·0	**Upward**					**0·9***	0·8	1·0	**Upward**
1990-1998	-0·4*	-0·7	-0·1					1990-1998	-0·1	-0·7	0·3				
1998-2016	1·7*	1·3	1·8					1998-2016	1·7*	0·5	1·9				
2016-2021	-0·1	-1·3	1·7					2016-2020	-0·8	-2·0	1·8				
2021-2023	2·9*	0·5	4·3					2020-2023	1·6*	0·0	3·5				
**Mesothelioma**				**-0·1***	-0·2	-0·03	**Downward**					**-0·1***	-0·2	-0·01	**Downward**
1990-2011	0·3*	0·2	0·5					1990-2010	0·5*	0·3	0·6				
2011-2023	-0·9*	-1·2	-0·6					2010-2023	-0·9*	-1·2	-0·7				
**Southern Latin America**															
**All cancers**				**2·3***	2·2	2·4	**Upward**					**2·3***	2·2	2·4	**Upward**
1990-1997	-0·1	-0·5	0·3					1990-1997	-0·0	-0·5	0·3				
1997-2006	7·3*	7·0	7·6					1997-2006	7·2*	1·5	7·6				
2006-2016	2·3*	2·0	2·7					2006-2013	2·6*	2·3	7·6				
2016-2020	-3·4*	-4·8	-2·0					2013-2017	0·7	-0·3	2·3				

2020-2023	0·9	-0·9	3·9					2017-2020	-3·9*	-4·8	-2·4				
-	-	-	-					2020-2023	1·1	-0·2	3·7				
**Ovarian**				**1·1***	1·0	1·3	**Upward**					**1·1***	1·0	1·2	**Upward**
1990-1997	-1·0*	-1·4	-0·6					1990-1997	-0·9*	-1·3	-0·6				
1997-2006	6·4*	6·1	6·7					1997-2006	6·2*	5·9	6·5				
2006-2017	0·1	-0·1	0·4					2006-2016	0·2	-0·0	0·6				
2017-2020	-4·8*	-5·8	-3·1					2016-2020	-3·5*	-4·9	-2·3				
2020-2023	1·0	-0·6	4·2					2020-2023	0·5	-1·0	3·3				
**Lung**				**2·8***	2·7	2·9	**Upward**					**2·7***	2·6	2·8	**Upward**
1990-1998	0·7*	0·2	1·1					1990-1997	0·3	-0·3	0·7				
1998-2005	8·0*	0·5	8·7					1997-2000	6·1*	1·0	7·3				
2005-2013	4·1*	3·7	8·5					2000-2005	8·5*	6·8	9·9				
2013-2016	1·5	-0·7	3·9					2005-2013	4·2*	3·9	5·1				
2016-2020	-3·1*	-4·5	-2·1					2013-2016	0·9	-1·3	3·4				
2020-2023	2·1*	0·6	4·8					2016-2020	-3·3*	-4·4	-2·5				
-	-	-	-					2020-2023	1·5*	0·2	3·9				
**Mesothelioma**				**2·3***	2·2	2·4	**Upward**					**2·3***	2·2	2·5	**Upward**
1990-1997	0·3	-0·6	1·0					1990-1997	0·4	-0·6	1·1				
1997-2005	8·2*	7·5	9·1					1997-2005	8·3*	7·5	9·3				
2005-2017	1·9*	1·6	2·4					2005-2017	1·8*	1·5	2·3				
2017-2023	-2·2*	-3·4	-1·3					2017-2023	-2·0*	-3·3	-1·1				

Table S5. Trends in age-standardized mortality and disability-adjusted life years (DALYs) rates for cancer in women attributable to occupational asbestos exposure between 1990 and 2023 by regions in the Americas.

All cancers: laryngeal, lung (trachea, bronchi and lungs) and ovarian cancers and mesothelioma; 95%UI: uncertainty interval 95%; APC: annual percentage change; AAPC: average annual percentage change; *significance: AAPC <> 0 and p-value < 0.05. Elaborated by the authors (2025).

Regions/Years	Mortality							DALY							
	**APC (%)**	95%UI		**AAPC (%) 1990-2023**	95%UI		**Trend 1990-2023**		**APC (%)**	95%UI		**AAPC (%) 1990-2023**	95%UI		**Trend 1990-2023**
**High-income North America**															
**All cancers**				**-2·5***	-2·5	-2·4	**Downward**					**-3·0***	-3·0	-2·9	**Downward**
1990-1995	0·1	-0·3	0·9					1990-1995	-0·1	-0·5	0·7				
1995-2002	-1·2*	-1·6	-0·9					1995-2002	-1·7*	-2·1	-1·3				
2002-2009	-2·6*	-3·0	-2·3					2002-2009	-3·5*	-3·9	-3·1				
2009-2019	-4·2*	-4·7	-4·0					2009-2017	-4·9*	-5·7	-4·6				
2019-2023	-3·1*	-3·8	-1·9					2017-2023	-3·5*	-3·9	-2·7				
**Laryngeal**				**-2·0***	-2·1	-1·9	**Downward**					**-2·4***	-2·5	-2·3	**Downward**
1990-1993	0·6	-0·6	2·5					1990-1994	0·3	-0·4	1·8				
1993-2001	-1·5*	-2·1	-1·2					1994-2002	-2·1*	-2·5	-1·7				
2001-2017	-3·3*	-3·5	-3·2					2002-2016	-4·2*	-4·4	-4·1				
2017-2023	-0·5	-1·0	0·2					2016-2023	-0·7*	-1·1	-0·1				
**Lung**				**-2·6***	-2·6	-2·5	**Downward**					**-3·0***	-3·1	-3·0	**Downward**
1990-1995	-0·0	-0·4	0·8					1990-1995	-0·3	-0·7	0·6				
1995-2002	-1·3*	-1·7	-1·0					1995-2002	-1·8*	-2·2	-1·4				
2002-2009	-2·7*	-3·1	-2·4					2002-2009	-3·6*	-4·0	-3·2				
2009-2018	-4·3*	-4·9	-4·1					2009-2017	-5·0*	-5·7	-4·7				
2018-2023	-3·3*	-3·8	-2·2					2017-2023	-3·3*	-3·8	-2·6				
**Mesothelioma**				**-1·5***	-1·6	-1·4	**Downward**					**-2·1***	-2·2	-2·0	**Downward**
1990-2000	1·1*	0·8	1·6					1990-1999	1·4*	0·9	1·9				
2000-2012	-1·2*	-1·6	-0·9					1999-2011	-2·0*	-2·3	-1·6				
2012-2023	-3·9*	-4·2	-3·6					2011-2023	-4·6*	-5·0	-4·4				
**Central Latin America**															
**All cancers**				**-1·2***	-1·2	-1·1	**Downward**					**-1·2***	-1·3	-1·1	**Downward**
1990-1995	0·3	-0·4	1·3					1990-1995	-0·1	-0·7	1·0				

1995-2001	-2·5*	-3·4	-2·0					1995-2001	-2·2*	-3·0	-1·8				
2001-2005	3·8*	2·7	5·4					2001-2005	4·0*	2·9	5·3				
2005-2018	-1·5*	-1·7	-1·3					2005-2019	-1·7*	-1·9	-1·6				
2018-2023	-3·9*	-4·8	-3·3					2019-2023	-4·4*	-5·8	-3·6				
**Laryngeal**				**-2·0***	-2·2	-1·9	**Downward**					**-2·2***	-2·3	-2·0	**Downward**
1990-1997	-1·4	-2·6	0·1					1990-1997	-1·4	-2·1	0·3				
1997-2000	-4·0	-4·9	0·2					1997-2000	-3·8	-4·7	0·4				
2000-2008	0·0	-3·7	1·7					2000-2008	0·0	-3·7	1·3				
2008-2014	-2·3*	-3·3	-0·1					2008-2020	-2·8*	-3·1	-1·7				
2014-2023	-3·6*	-5·1	-3·1					2020-2023	-5·6*	-8·3	-3·8				
**Lung**				**-1·4***	-1·5	-1·4	**Downward**					**-1·6***	-1·6	-1·5	**Downward**
1990-1995	0·3	-0·3	1·1					1990-1995	-0·1	-0·7	0·8				
1995-2001	-2·9*	-3·5	-2·4					1995-2001	-2·7*	-3·5	-2·2				
2001-2005	3·1*	2·2	4·2					2001-2005	3·0*	1·9	4·3				
2005-2017	-1·8*	-2·0	-1·6					2005-2017	-2·0*	-2·2	-1·8				
2017-2023	-3·7*	-4·3	-3·3					2017-2023	-3·7*	-4·3	-3·2				
**Mesothelioma**				**0·2***	0·1	0·3	**Upward**					**0·1**	-0·1	0·2	**Stable**
1990-2001	-0·1	-0·5	0·3					1990-2001	0·1	-0·4	0·5				
2001-2006	5·8*	4·3	9·0					2001-2006	6·3*	2·1	8·3				
2006-2019	-0·3	-0·6	0·1					2006-2016	-1·1	-2·4	5·4				
2019-2023	-4·6*	-7·3	-2·9					2016-2019	1·9	-0·6	3·1				
-	-	-	-					2019-2023	-5·8*	-8·3	-4·5				
**Caribbean**															
**All cancers**				**-0·6***	-0·8	-0·5	**Downward**					**-0·7***	-0·8	-0·6	**Downward**
1990-2010	-0·3	-0·6	0·0					1990-1997	0·3	-0·4	1·3				
2010-2013	-3·7*	-4·8	-0·2					1997-2005	-1·3*	-3·0	-0·1				
2013-2023	-0·4	-1·1	1·5					2005-2009	1·7	-1·3	3·3				

-	-	-	-					2009-2013	-4·8	-6·6	1·9				
-	-	-	-					2013-2016	1·5	-5·1	2·6				
-	-	-	-					2016-2023	-0·9*	-2·2	-0·3				
**Laryngeal**				**-0·2**	-0·3	0·0	**Stable**					**0·1**	-0·0	0·2	**Stable**
1990-1998	1·7*	0·3	2·6					1990-1998	1·5*	1·0	2·3				
1998-2001	-4·7	-5·8	2·8					1998-2002	-3·4*	-5·4	-1·9				
2001-2008	1·0	-4·7	3·6					2002-2007	1·9*	0·8	4·4				
2008-2013	-2·2	-4·5	1·4					2007-2013	-1·6*	-3·7	-0·8				
2013-2021	0·8	-2·9	3·4					2013-2023	0·5*	0·1	1·2				
2021-2023	-3·2	-6·4	0·4					-	-	-	-				
**Lung**				**-0·8***	-0·9	-0·7	**Downward**					**-0·9***	-1·0	-0·8	**Downward**
1990-2006	-0·5*	-1·3	-0·3					1990-1997	0·1	-0·4	1·0				
2006-2009	2·2	-3·1	3·3					1997-2005	-1·4*	-2·8	-0·9				
2009-2012	-5·2*	-6·3	-0·2					2005-2009	1·6*	0·1	3·0				
2012-2023	-0·7	-1·1	0·1					2009-2013	-4·8*	-6·5	-3·4				
-	-	-	-					2013-2017	0·8	-0·4	2·3				
-	-	-	-					2017-2023	-1·4*	-2·8	-0·8				
**Mesothelioma**				**-0·3***	-0·5	-0·1	**Downward**					**-0·3***	-0·5	-0·2	**Downward**
1990-2023	-0·3*	-0·5	-0·1					1990-2023	-0·3*	-0·5	-0·2				
**Andean Latin America**															
**All cancers**				**-0·7***	-0·9	-0·4	**Downward**					**-0·8***	-1·0	-0·5	**Downward**
1990-1995	7·5*	6·3	10·0					1990-1996	7·3*	5·7	9·0				
1995-1998	-1·4	-7·5	4·3					1996-2001	-8·1*	-11·3	-5·9				
1998-2001	-11·1*	-12·7	-1·3					2001-2016	-2·7*	-3·1	-2·2				
2001-2004	0·5	-3·2	2·3					2016-2021	5·8*	3·8	10·4				
2004-2016	-2·8*	-3·9	-2·3					2021-2023	-6·4*	-11·1	-0·1				
2016-2021	4·7*	3·4	8·3					-	-	-	-				

2021-2023	-4·2	-8·0	0·1					-	-	-	-				
**Laryngeal**				**-0·6***	-0·8	-0·4	**Downward**					**-0·6***	-0·8	-0·3	**Downward**
1990-1997	5·7*	4·4	7·2					1990-1997	5·9*	4·3	7·9				
1997-2001	-11·9*	-15·1	-8·8					1997-2001	-12·3*	-16·0	-8·7				
2001-2014	-2·2*	-2·9	-1·6					2001-2014	-2·3*	-3·0	-1·6				
2014-2023	2·2*	1·3	3·5					2014-2023	2·6*	1·6	4·3				
**Lung**				**-0·9***	-1·1	-0·7	**Downward**					**-1·1***	-1·3	-0·9	**Downward**
1990-1996	6·9*	6·1	7·8					1990-1996	7·0*	6·1	7·9				
1996-2001	-9·1*	-10·5	-8·2					1996-2001	-10·0*	-11·4	-9·0				
2001-2004	0·9	-1·6	2·2					2001-2004	0·4	-2·2	1·8				
2004-2016	-3·0*	-3·6	-2·8					2004-2016	-3·1*	-3·9	-2·8				
2016-2021	4·1*	3·0	6·6					2016-2021	5·4*	4·1	8·3				
2021-2023	-3·9	-7·0	0·2					2021-2023	-6·4*	-9·6	-1·8				
**Mesothelioma**				**0·6***	0·3	1·1	**Upward**					**0·6***	0·3	1·1	**Upward**
1990-1999	6·4*	5·0	8·8					1990-1999	6·7*	5·1	9·3				
1999-2002	-13·0*	-15·6	-6·4					1999-2002	-13·5*	-16·2	-6·0				
2002-2014	-2·2*	-3·4	-0·6					2002-2014	-2·4*	-3·8	-0·5				
2014-2023	3·8*	2·3	7·0					2014-2023	4·1*	2·4	7·5				
**Tropical Latin America**															
**All cancers**				**-0·2***	-0·3	-0·1	**Downward**					**-0·6***	-0·7	-0·5	**Downward**
1990-1997	-0·6	-1·7	0·0					1990-1996	-0·8*	-2·5	-0·1				
1997-2011	0·9*	0·7	1·3					1996-2012	0·3*	0·1	0·7				
2011-2023	-1·3*	-1·6	-1·1					2012-2023	-1·7*	-2·0	-1·5				
**Laryngeal**				**-0·3***	-0·4	-0·2	**Downward**					**-0·5***	-0·6	-0·5	**Downward**
1990-1993	0·9*	0·1	2·4					1990-1993	1·6*	0·4	4·0				
1993-1996	-2·8*	-3·4	-1·7					1993-1996	-2·8*	-3·7	-1·2				
1996-2002	-0·3	-0·9	0·6					1996-2003	-0·5	-0·9	1·0				

2002-2009	1·2*	0·1	1·9					2003-2012	1·2*	0·9	1·7				
2009-2012	2·3	-3·0	2·8					2012-2019	-2·6*	-3·4	-2·2				
2012-2019	-2·4*	-3·2	-1·6					2019-2023	-0·5	-1·4	1·1				
2019-2023	-0·1	-0·8	1·4					-	-	-	-				
**Lung**				**-0·3***	-0·4	-0·2	**Downward**					**-0·8***	-0·9	-0·7	**Downward**
1990-1997	-0·7*	-1·9	-0·1					1990-1997	-0·9*	-2·3	-0·3				
1997-2012	0·8*	0·6	1·1					1997-2011	0·2	-0·0	0·6				
2012-2023	-1·5*	-1·9	-1·2					2011-2023	-1·8*	-2·2	-1·5				
**Mesothelioma**				**0·2***	0·2	0·3	**Upward**					0·03	-0·04	0·1	**Stable**
1990-1996	-0·5*	-1·0	-0·05					1990-1996	-0·5	-1·3	0·1				
1996-2000	3·9*	2·9	5·2					1996-2000	3·6*	2·4	5·0				
2000-2004	-0·4	-1·3	0·5					2000-2004	-0·8	-2·0	0·2				
2004-2012	1·2*	1·0	2·0					2004-2011	1·2*	0·7	2·3				
2012-2015	-2·6*	-3·2	-1·3					2011-2017	-1·9*	-3·1	-1·3				
2015-2023	-0·7*	-1·0	-0·1					2017-2023	-0·6	-1·1	0·7				
**Southern Latin America**															
**All cancers**				**-0·3***	-0·5	-0·2	**Downward**					**-0·6***	-0·7	-0·5	**Downward**
1990-1994	-1·4*	-3·7	-0·2					1990-1995	-1·1*	-3·2	-0·2				
1994-2000	1·0*	0·2	4·2					1995-2000	0·9*	0·0	5·2				
2000-2004	5·1	-0·6	6·7					2000-2004	5·2	-2·1	6·7				
2004-2016	-1·1*	-1·4	-0·6					2004-2016	-1·6*	-1·9	-1·0				
2016-2020	-4·2*	-5·6	-2·9					2016-2020	-4·5*	-5·9	-3·4				
2020-2023	-0·4	-2·0	2·3					2020-2023	-0·1	-1·7	2·8				
**Laryngeal**				**-1·2***	-1·3	-1·0	**Downward**					**-1·5***	-1·6	-1·3	**Downward**
1990-1998	-1·4*	-2·1	-0·7					1990-1998	-1·4*	-2·2	-0·8				
1998-2004	4·6*	3·4	6·4					1998-2004	4·2*	3·0	6·0				
2004-2017	-2·3*	-2·6	-1·9					2004-2017	-3·0*	-3·2	-2·4				

2017-2020	-7·0*	-8·2	-4·7					2017-2020	-6·6*	-7·9	-4·1				
2020-2023	-0·7	-2·9	3·0					2020-2023	-0·7	-3·2	3·1				
**Lung**				**-0·4***	-0·6	-0·3	**Downward**					**-0·7***	-0·8	-0·6	**Downward**
1990-1994	-1·4*	-3·5	-0·3					1990-1994	-1·5*	-3·8	-0·3				
1994-2000	0·9*	0·2	3·2					1994-2000	0·6	-0·1	4·7				
2000-2004	4·8*	0·4	6·4					2000-2004	5·0	-1·8	6·5				
2004-2016	-1·1*	-1·4	-0·8					2004-2016	-1·7*	-1·9	-1·3				
2016-2020	-4·3*	-5·7	-3·2					2016-2020	-4·6*	-6·0	-3·6				
2020-2023	-0·5	-2·0	2·0					2020-2023	-0·5	-2·0	2·3				
**Mesothelioma**				**0·8***	0·6	0·9	**Upward**					**0·6***	0·5	0·8	**Upward**
1990-1998	-0·1	-1·2	0·8					1990-1998	0·0	-1·2	1·0				
1998-2004	7·1*	5·5	10·4					1998-2004	6·9*	5·2	10·7				
2004-2023	-0·8*	-1·0	-0·5					2004-2023	-1·0*	-1·3	-0·8				

Table S6. Trends in age-standardized mortality and disability-adjusted life years (DALYs) rates for cancer in men attributable to occupational asbestos exposure between 1990 and 2023 by regions in the Americas.

All cancers: laryngeal and lung (trachea, bronchi and lungs) cancers and mesothelioma; 95%UI: uncertainty interval 95%; APC: annual percentage change; AAPC: average annual percentage change; *significance: AAPC <> 0 and p-value < 0.05.

Elaborated by the authors (2025).

High-income North America																		
	Mortality									DALY								
	Laryngeal			Lung			Mesothelioma			Laryngeal			Lung			Mesothelioma		
**Age-group**	**MR**	**95%UI**		**MR**	**95%UI**		**MR**	**95%UI**		**DR**	**95%UI**		**DR**	**95%UI**		**DR**	**95%UI**	
50-54	**0·03**	0·02	0·03	**1·88**	1·82	1·93	**0·63**	0·59	0·66	**1·08**	1·03	1·12	**71·98**	71·63	72·34	**24·09**	23·85	24·33
55-59	**0·11**	0·09	0·13	**7·95**	7·81	8·09	**1·41**	1·35	1·47	**3·97**	3·87	4·07	**268·00**	267·15	268·84	**47·62**	47·23	48·02
60-64	**0·34**	0·31	0·38	**26·80**	26·49	27·12	**2·78**	2·68	2·88	**10·84**	10·63	11·04	**781·93**	780·16	783·72	**81·66**	81·06	82·25
65-69	**0·80**	0·74	0·87	**66·97**	66·36	67·59	**5·04**	4·88	5·21	**21·24**	20·88	21·60	**1651·87**	1648·64	1655·10	**125·24**	124·37	126·12
70-74	**1·62**	1·50	1·75	**143·89**	142·72	145·08	**9·13**	8·85	9·42	**35·47**	34·89	36·05	**2920·28**	2914·88	2925·69	**186·52**	185·23	187·81
75-79	**3·01**	2·80	3·25	**257·98**	255·84	260·14	**16·27**	15·78	16·76	**52·59**	51·73	53·46	**4177·31**	4169·40	4185·23	**265·39**	263·57	267·23
80 plus	**5·30**	4·90	5·74	**398·99**	395·26	402·75	**26·18**	25·42	26·95	**63·35**	62·28	64·43	**4522·05**	4512·80	4531·32	**295·89**	293·87	297·93
**Period**	**RR**	**95%UI**		**RR**	**95%UI**		**RR**	**95%UI**		**RR**	**95%UI**		**RR**	**95%UI**		**RR**	**95%UI**	
1994	**1·78**	1·64	1·93	**1·71**	1·69	1·73	**1·38**	1·34	1·42	**1·72**	1·69	1·75	**1·68**	1·68	1·69	**1·33**	1·32	1·34
1999	**1·36**	1·27	1·46	**1·31**	1·30	1·32	**1·20**	1·17	1·24	**1·35**	1·33	1·37	**1·31**	1·31	1·32	**1·19**	1·19	1·20
2004	***1·00***	*1·00*	*1·00*	***1·00***	*1·00*	*1·00*	***1·00***	*1·00*	*1·00*	***1·00***	*1·00*	*1·00*	***1·00***	*1·00*	*1·00*	***1·00***	*1·00*	*1·00*
2009	**0·78**	0·73	0·84	**0·78**	0·77	0·78	**0·87**	0·85	0·90	**0·77**	0·76	0·78	**0·77**	0·77	0·77	**0·87**	0·86	0·87
2014	**0·66**	0·61	0·71	**0·61**	0·60	0·61	**0·73**	0·71	0·75	**0·64**	0·63	0·65	**0·60**	0·59	0·60	**0·71**	0·71	0·72
2019	**0·64**	0·58	0·70	**0·52**	0·51	0·52	**0·59**	0·58	0·61	**0·63**	0·62	0·64	**0·51**	0·51	0·51	**0·57**	0·57	0·58
**Cohort**	**RR**	**95%UI**		**RR**	**95%UI**		**RR**	**95%UI**		**RR**	**95%UI**		**RR**	**95%UI**		**RR**	**95%UI**	
1914	**0·46**	0·41	0·53	**0·44**	0·43	0·45	**0·48**	0·45	0·50	**0·49**	0·47	0·50	**0·46**	0·46	0·46	**0·51**	0·50	0·52
1919	**0·60**	0·55	0·67	**0·58**	0·57	0·59	**0·61**	0·59	0·64	**0·62**	0·61	0·64	**0·60**	0·60	0·60	**0·64**	0·64	0·65
1924	**0·78**	0·72	0·85	**0·76**	0·75	0·76	**0·77**	0·75	0·80	**0·79**	0·78	0·80	**0·76**	0·76	0·77	**0·79**	0·79	0·80
1929	**0·93**	0·87	0·99	**0·91**	0·91	0·92	**0·91**	0·88	0·93	**0·93**	0·91	0·94	**0·91**	0·91	0·92	**0·92**	0·91	0·92
1934	***1·00***	*1·00*	*1·00*	***1·00***	*1·00*	*1·00*	***1·00***	*1·00*	*1·00*	***1·00***	*1·00*	*1·00*	***1·00***	*1·00*	*1·00*	***1·00***	*1·00*	*1·00*
1939	**0·99**	0·93	1·06	**1·01**	1·00	1·01	**1·07**	1·04	1·10	**1·01**	0·99	1·02	**1·02**	1·01	1·02	**1·07**	1·06	1·08
1944	**0·94**	0·86	1·02	**0·96**	0·95	0·97	**1·02**	0·99	1·05	**0·96**	0·95	0·98	**0·98**	0·98	0·98	**1·04**	1·03	1·05
1949	**0·90**	0·81	0·99	**0·88**	0·87	0·89	**0·91**	0·88	0·95	**0·91**	0·89	0·92	**0·89**	0·89	0·89	**0·93**	0·92	0·94
1954	**0·84**	0·73	0·95	**0·82**	0·81	0·84	**0·79**	0·76	0·83	**0·85**	0·83	0·87	**0·83**	0·83	0·84	**0·81**	0·81	0·82
1959	**0·77**	0·64	0·91	**0·77**	0·75	0·79	**0·71**	0·68	0·75	**0·78**	0·76	0·80	**0·78**	0·78	0·78	**0·73**	0·72	0·74
1964	**0·68**	0·50	0·91	**0·66**	0·63	0·68	**0·70**	0·65	0·76	**0·69**	0·66	0·73	**0·67**	0·66	0·67	**0·72**	0·71	0·73
1969	**0·58**	0·27	1·23	**0·51**	0·46	0·57	**0·67**	0·58	0·78	**0·59**	0·52	0·66	**0·52**	0·51	0·53	**0·70**	0·68	0·71
**Central Latin America**																		
	Mortality									DALY								
	Laryngeal			Lung			Mesothelioma			Laryngeal			Lung			Mesothelioma		
**Age-group**	**MR**	**95%UI**		**MR**	**95%UI**		**MR**	**95%UI**		**DR**	**95%UI**		**DR**	**95%UI**		**DR**	**95%UI**	
50-54	**0·02**	0·01	0·03	**0·38**	0·34	0·42	**0·46**	0·41	0·53	**0·68**	0·63	0·74	**14·61**	14·34	14·88	**17·98**	17·52	18·45
55-59	**0·06**	0·05	0·09	**1·31**	1·22	1·41	**0·79**	0·70	0·89	**2·19**	2·05	2·33	**44·54**	43·92	45·17	**26·89**	26·24	27·55
60-64	**0·10**	0·08	0·14	**2·25**	2·11	2·40	**0·95**	0·85	1·07	**3·10**	2·92	3·29	**65·78**	64·93	66·64	**28·02**	27·36	28·70
65-69	**0·24**	0·19	0·32	**5·44**	5·15	5·75	**1·44**	1·29	1·62	**6·06**	5·73	6·41	**133·89**	132·30	135·49	**35·72**	34·87	36·60
70-74	**0·47**	0·37	0·60	**10·59**	10·06	11·15	**2·05**	1·84	2·30	**9·61**	9·10	10·15	**214·24**	211·76	216·74	**41·83**	40·82	42·87

75-79	0·72	0·57	0·92	15·75	14·95	16·58	2·31	2·06	2·59	11·87	11·23	12·54	255·40	252·37	258·48	37·79	36·80	38·81
80 plus	0·95	0·75	1·21	20·42	19·40	21·49	2·29	2·05	2·57	10·97	10·37	11·61	233·09	230·25	235·95	26·30	25·56	27·06
**Period**	**RR**	**95%UI**		**RR**	**95%UI**		**RR**	**95%UI**		**RR**	**95%UI**		**RR**	**95%UI**		**RR**	**95%UI**	
1994	1·17	0·90	1·53	1·10	1·04	1·17	0·79	0·71	0·89	1·18	1·11	1·24	1·10	1·09	1·12	0·78	0·76	0·80
1999	1·04	0·82	1·31	0·99	0·94	1·04	0·82	0·74	0·91	1·04	0·98	1·09	0·98	0·97	1·00	0·82	0·80	0·83
2004	*1·00*	*1·00*	*1·00*	*1·00*	*1·00*	*1·00*	*1·00*	*1·00*	*1·00*	*1·00*	*1·00*	*1·00*	*1·00*	*1·00*	*1·00*	*1·00*	*1·00*	*1·00*
2009	0·92	0·74	1·14	0·91	0·87	0·95	1·01	0·92	1·10	0·92	0·87	0·96	0·90	0·89	0·91	0·99	0·97	1·01
2014	0·79	0·63	0·98	0·82	0·78	0·86	1·00	0·91	1·09	0·79	0·75	0·83	0·82	0·81	0·83	0·98	0·96	1·00
2019	0·66	0·53	0·83	0·70	0·67	0·73	0·95	0·87	1·03	0·67	0·64	0·70	0·70	0·69	0·71	0·94	0·93	0·96
**Cohort**	**RR**	**95%UI**		**RR**	**95%UI**		**RR**	**95%UI**		**RR**	**95%UI**		**RR**	**95%UI**		**RR**	**95%UI**	
1914	0·95	0·61	1·48	0·91	0·82	1·00	1·03	0·79	1·35	0·93	0·83	1·04	0·89	0·87	0·92	1·03	0·96	1·12
1919	0·89	0·64	1·24	0·85	0·79	0·91	0·95	0·79	1·14	0·88	0·82	0·96	0·84	0·83	0·86	0·96	0·91	1·00
1924	0·95	0·73	1·23	0·92	0·87	0·98	0·98	0·85	1·13	0·95	0·89	1·01	0·93	0·91	0·94	1·00	0·96	1·03
1929	0·97	0·77	1·23	0·97	0·92	1·02	0·99	0·88	1·11	0·98	0·92	1·03	0·97	0·96	0·98	0·99	0·96	1·02
1934	*1·00*	*1·00*	*1·00*	*1·00*	*1·00*	*1·00*	*1·00*	*1·00*	*1·00*	*1·00*	*1·00*	*1·00*	*1·00*	*1·00*	*1·00*	*1·00*	*1·00*	*1·00*
1939	1·01	0·82	1·25	1·01	0·97	1·06	1·02	0·92	1·13	1·02	0·97	1·07	1·01	1·00	1·02	1·03	1·01	1·06
1944	1·02	0·81	1·29	1·00	0·95	1·05	1·06	0·95	1·17	1·02	0·97	1·07	1·00	0·99	1·01	1·06	1·04	1·09
1949	1·02	0·78	1·32	0·99	0·94	1·05	1·09	0·99	1·20	1·02	0·97	1·07	1·00	0·99	1·01	1·10	1·08	1·12
1954	1·01	0·75	1·36	0·99	0·93	1·06	1·06	0·96	1·18	1·01	0·95	1·07	0·99	0·98	1·00	1·07	1·04	1·09
1959	0·95	0·66	1·37	0·94	0·87	1·02	1·00	0·89	1·12	0·95	0·88	1·01	0·94	0·92	0·95	1·01	0·98	1·03
1964	0·90	0·54	1·49	0·88	0·79	0·98	0·90	0·78	1·03	0·89	0·81	0·97	0·87	0·85	0·89	0·90	0·88	0·93
1969	0·86	0·30	2·46	0·81	0·65	1·02	0·84	0·69	1·04	0·85	0·72	1·01	0·81	0·78	0·84	0·84	0·81	0·87
**Caribbean**																		
	**Mortality**									**DALY**								
	**Laryngeal**			**Lung**			**Mesothelioma**			**Laryngeal**			**Lung**			**Mesothelioma**		
**Age-group**	**MR**	**95%UI**		**MR**	**95%UI**		**MR**	**95%UI**		**DR**	**95%UI**		**DR**	**95%UI**		**DR**	**95%UI**	
50-54	0·05	0·02	0·09	0·70	0·60	0·83	0·37	0·27	0·50	1·81	1·61	2·03	27·01	26·24	27·80	14·11	13·26	15·02
55-59	0·15	0·09	0·24	2·30	2·05	2·58	0·68	0·51	0·90	5·21	4·73	5·73	77·53	75·75	79·35	22·89	21·56	24·31
60-64	0·14	0·09	0·23	2·32	2·07	2·61	0·41	0·30	0·56	4·22	3·82	4·66	67·51	65·92	69·14	11·96	11·22	12·75
65-69	0·23	0·14	0·36	4·13	3·71	4·61	0·57	0·41	0·78	5·69	5·15	6·29	101·32	98·98	103·71	13·93	13·04	14·89
70-74	0·46	0·30	0·71	9·34	8·46	10·31	0·89	0·65	1·20	9·54	8·65	10·51	188·57	184·43	192·80	17·96	16·79	19·20
75-79	1·01	0·67	1·51	20·57	18·78	22·53	1·72	1·29	2·29	16·72	15·24	18·33	332·32	325·36	339·43	27·94	26·18	29·82
80 plus	1·44	0·98	2·11	32·20	29·51	35·13	2·18	1·67	2·86	16·37	14·95	17·92	355·71	348·39	363·18	24·38	22·84	26·02
**Period**	**RR**	**95%UI**		**RR**	**95%UI**		**RR**	**95%UI**		**RR**	**95%UI**		**RR**	**95%UI**		**RR**	**95%UI**	
1994	1·02	0·67	1·57	1·14	1·03	1·25	1·05	0·80	1·36	1·01	0·92	1·10	1·13	1·11	1·15	1·04	0·99	1·10
1999	0·96	0·66	1·40	1·03	0·95	1·13	1·00	0·79	1·28	0·96	0·89	1·04	1·04	1·02	1·06	1·00	0·96	1·05
2004	*1·00*	*1·00*	*1·00*	*1·00*	*1·00*	*1·00*	*1·00*	*1·00*	*1·00*	*1·00*	*1·00*	*1·00*	*1·00*	*1·00*	*1·00*	*1·00*	*1·00*	*1·00*
2009	0·96	0·68	1·37	0·95	0·87	1·03	0·98	0·78	1·24	0·96	0·89	1·04	0·94	0·92	0·96	0·98	0·93	1·03
2014	0·95	0·67	1·35	0·89	0·82	0·96	0·95	0·75	1·19	0·94	0·88	1·02	0·88	0·86	0·89	0·94	0·90	0·99
2019	0·96	0·69	1·35	0·84	0·78	0·91	0·93	0·74	1·17	0·96	0·90	1·04	0·84	0·83	0·85	0·93	0·89	0·98

Cohort	RR	95%UI		RR	95%UI		RR	95%UI		RR	95%UI		RR	95%UI		RR	95%UI	
1914	**1·12**	0·60	2·09	**0·87**	0·75	1·00	**0·94**	0·60	1·49	**1·16**	0·99	1·35	**0·89**	0·86	0·92	**0·96**	0·85	1·08
1919	**1·12**	0·69	1·81	**0·97**	0·87	1·08	**1·00**	0·71	1·42	**1·13**	1·01	1·27	**0·98**	0·95	1·00	**1·01**	0·92	1·10
1924	**1·07**	0·71	1·63	**1·02**	0·93	1·12	**1·02**	0·75	1·38	**1·07**	0·97	1·19	**1·02**	0·99	1·04	**1·02**	0·95	1·10
1929	**1·05**	0·72	1·54	**1·03**	0·95	1·12	**1·02**	0·77	1·35	**1·05**	0·96	1·15	**1·03**	1·01	1·05	**1·02**	0·95	1·09
1934	***1·00***	*1·00*	*1·00*	***1·00***	*1·00*	*1·00*	***1·00***	*1·00*	*1·00*	***1·00***	*1·00*	*1·00*	***1·00***	*1·00*	*1·00*	***1·00***	*1·00*	*1·00*
1939	**0·99**	0·69	1·41	**1·01**	0·93	1·10	**0·99**	0·77	1·27	**0·99**	0·91	1·08	**1·02**	1·00	1·04	**0·99**	0·94	1·05
1944	**1·03**	0·70	1·52	**1·06**	0·97	1·16	**1·00**	0·77	1·30	**1·04**	0·95	1·13	**1·07**	1·05	1·10	**1·01**	0·95	1·06
1949	**1·04**	0·68	1·59	**1·07**	0·97	1·18	**0·99**	0·76	1·30	**1·05**	0·96	1·15	**1·08**	1·05	1·10	**1·00**	0·95	1·06
1954	**1·09**	0·69	1·71	**1·04**	0·93	1·17	**1·00**	0·76	1·31	**1·09**	1·00	1·20	**1·04**	1·02	1·07	**1·00**	0·95	1·06
1959	**1·08**	0·66	1·78	**0·98**	0·86	1·12	**1·00**	0·76	1·32	**1·09**	0·99	1·20	**0·99**	0·96	1·01	**1·01**	0·95	1·07
1964	**1·11**	0·61	2·00	**0·93**	0·79	1·10	**1·00**	0·74	1·37	**1·12**	1·00	1·25	**0·94**	0·91	0·97	**1·01**	0·94	1·07
1969	**0·97**	0·30	3·12	**0·86**	0·62	1·19	**1·01**	0·63	1·61	**0·98**	0·80	1·19	**0·86**	0·82	0·91	**1·01**	0·92	1·10
**Andean Latin America**																		
	**Mortality**									**DALY**								
	**Laryngeal**			**Lung**			**Mesothelioma**			**Laryngeal**			**Lung**			**Mesothelioma**		
**Age-group**	**MR**	**95%UI**		**MR**	**95%UI**		**MR**	**95%UI**		**DR**	**95%UI**		**DR**	**95%UI**		**DR**	**95%UI**	
50-54	**0·01**	0·00	0·03	**0·33**	0·26	0·41	**0·42**	0·32	0·55	**0·31**	0·24	0·39	**12·67**	12·18	13·18	**16·42**	15·58	17·30
55-59	**0·02**	0·01	0·06	**0·85**	0·72	1·00	**0·63**	0·49	0·80	**0·75**	0·62	0·90	**28·69**	27·78	29·63	**21·21**	20·15	22·33
60-64	**0·03**	0·01	0·07	**1·05**	0·90	1·24	**0·47**	0·36	0·62	**0·82**	0·68	0·99	**30·64**	29·68	31·64	**13·82**	13·09	14·59
65-69	**0·08**	0·04	0·17	**2·86**	2·51	3·27	**0·86**	0·67	1·11	**2·00**	1·70	2·36	**70·13**	68·15	72·17	**21·11**	20·01	22·28
70-74	**0·21**	0·11	0·41	**7·49**	6·67	8·42	**1·73**	1·36	2·20	**4·28**	3·67	4·99	**151·39**	147·44	155·45	**35·06**	33·27	36·95
75-79	**0·46**	0·25	0·86	**14·80**	13·26	16·51	**2·34**	1·84	2·97	**7·60**	6·57	8·79	**239·85**	233·79	246·06	**38·05**	36·03	40·18
80 plus	**0·84**	0·46	1·54	**28·73**	25·86	31·92	**3·15**	2·52	3·94	**9·53**	8·27	10·97	**318·28**	310·47	326·28	**35·46**	33·59	37·43
**Period**	**RR**	**95%UI**		**RR**	**95%UI**		**RR**	**95%UI**		**RR**	**95%UI**		**RR**	**95%UI**		**RR**	**95%UI**	
1994	**1·76**	0·90	3·47	**1·73**	1·54	1·94	**1·31**	1·04	1·64	**1·76**	1·52	2·04	**1·75**	1·71	1·80	**1·32**	1·26	1·38
1999	**1·23**	0·67	2·25	**1·14**	1·03	1·27	**1·26**	1·03	1·55	**1·23**	1·07	1·41	**1·15**	1·12	1·18	**1·27**	1·21	1·32
2004	***1·00***	*1·00*	*1·00*	***1·00***	*1·00*	*1·00*	***1·00***	*1·00*	*1·00*	***1·00***	*1·00*	*1·00*	***1·00***	*1·00*	*1·00*	***1·00***	*1·00*	*1·00*
2009	**0·95**	0·53	1·69	**0·87**	0·79	0·96	**0·90**	0·74	1·10	**0·94**	0·83	1·08	**0·87**	0·85	0·89	**0·89**	0·86	0·93
2014	**0·93**	0·52	1·67	**0·80**	0·73	0·89	**0·90**	0·74	1·09	**0·92**	0·81	1·05	**0·80**	0·78	0·82	**0·89**	0·86	0·93
2019	**1·04**	0·58	1·83	**0·88**	0·80	0·98	**1·05**	0·87	1·26	**1·03**	0·91	1·17	**0·89**	0·87	0·91	**1·06**	1·01	1·10
**Cohort**	**RR**	**95%UI**		**RR**	**95%UI**		**RR**	**95%UI**		**RR**	**95%UI**		**RR**	**95%UI**		**RR**	**95%UI**	
1914	**1·00**	0·40	2·54	**0·82**	0·69	0·96	**1·11**	0·75	1·63	**1·01**	0·81	1·27	**0·81**	0·78	0·85	**1·10**	0·99	1·22
1919	**1·05**	0·50	2·21	**0·96**	0·84	1·10	**1·05**	0·78	1·41	**1·05**	0·88	1·25	**0·95**	0·92	0·98	**1·04**	0·97	1·12
1924	**1·04**	0·55	1·96	**1·02**	0·92	1·14	**0·99**	0·77	1·27	**1·04**	0·89	1·20	**1·01**	0·99	1·04	**0·98**	0·92	1·04
1929	**1·02**	0·58	1·80	**1·03**	0·93	1·14	**0·98**	0·78	1·24	**1·03**	0·89	1·18	**1·03**	1·00	1·05	**0·98**	0·93	1·04
1934	***1·00***	*1·00*	*1·00*	***1·00***	*1·00*	*1·00*	***1·00***	*1·00*	*1·00*	***1·00***	*1·00*	*1·00*	***1·00***	*1·00*	*1·00*	***1·00***	*1·00*	*1·00*
1939	**0·93**	0·55	1·57	**0·93**	0·84	1·02	**0·97**	0·79	1·20	**0·94**	0·82	1·06	**0·93**	0·91	0·96	**0·98**	0·93	1·03
1944	**0·96**	0·52	1·78	**0·97**	0·87	1·09	**1·00**	0·81	1·24	**0·96**	0·84	1·11	**0·97**	0·94	0·99	**1·00**	0·96	1·05
1949	**1·01**	0·49	2·06	**0·99**	0·87	1·12	**1·03**	0·83	1·29	**1·00**	0·86	1·17	**0·98**	0·96	1·01	**1·03**	0·98	1·08

1954	**0·99**	0·41	2·36	**0·95**	0·82	1·11	**1·01**	0·80	1·28	**1·00**	0·84	1·18	**0·95**	0·92	0·98	**1·01**	0·97	1·06
1959	**1·02**	0·35	2·95	**0·96**	0·80	1·16	**1·01**	0·79	1·29	**1·02**	0·84	1·24	**0·96**	0·93	0·99	**1·01**	0·96	1·06
1964	**1·06**	0·28	3·97	**1·00**	0·79	1·25	**1·02**	0·77	1·34	**1·06**	0·84	1·34	**0·99**	0·95	1·03	**1·01**	0·96	1·07
1969	**1·07**	0·10	11·84	**0·99**	0·65	1·49	**0·99**	0·67	1·46	**1·06**	0·71	1·58	**0·97**	0·91	1·04	**0·97**	0·91	1·05
**Tropical LatinAmerica**																		
	**Mortality**									**DALY**								
	**Laryngeal**			**Lung**			**Mesothelioma**			**Laryngeal**			**Lung**			**Mesothelioma**		
**Age-group**	**MR**	**95%UI**		**MR**	**95%UI**		**MR**	**95%UI**		**DR**	**95%UI**		**DR**	**95%UI**		**DR**	**95%UI**	
50-54	**0·23**	0·19	0·27	**2·13**	2·02	2·25	**1·28**	1·15	1·42	**8·83**	8·50	9·17	**81·58**	80·75	82·41	**49·22**	48·12	50·35
55-59	**0·54**	0·45	0·64	**5·87**	5·62	6·14	**2·06**	1·85	2·28	**18·40**	17·75	19·07	**197·55**	195·75	199·36	**69·38**	67·84	70·95
60-64	**0·20**	0·16	0·24	**2·89**	2·75	3·05	**0·56**	0·50	0·64	**5·78**	5·55	6·03	**84·07**	83·19	84·96	**16·41**	15·99	16·84
65-69	**0·33**	0·27	0·40	**5·80**	5·53	6·08	**0·81**	0·72	0·92	**8·17**	7·83	8·52	**142·20**	140·77	143·63	**19·94**	19·41	20·47
70-74	**0·50**	0·41	0·61	**10·51**	10·05	10·99	**1·06**	0·93	1·20	**10·26**	9·83	10·71	**212·11**	210·00	214·24	**21·44**	20·85	22·04
75-79	**1·60**	1·35	1·89	**35·30**	33·96	36·69	**3·34**	3·00	3·72	**26·30**	25·33	27·31	**569·69**	564·64	574·78	**54·25**	52·92	55·60
80 plus	**2·49**	2·13	2·92	**55·29**	53·28	57·37	**4·86**	4·39	5·37	**28·20**	27·17	29·27	**617·57**	612·16	623·02	**54·17**	52·86	55·50
**Period**	**RR**	**95%UI**		**RR**	**95%UI**		**RR**	**95%UI**		**RR**	**95%UI**		**RR**	**95%UI**		**RR**	**95%UI**	
1994	**1·08**	0·91	1·28	**1·14**	1·09	1·19	**0·91**	0·82	1·00	**1·08**	1·04	1·11	**1·13**	1·12	1·14	**0·91**	0·90	0·93
1999	**1·01**	0·86	1·17	**1·07**	1·03	1·11	**0·99**	0·91	1·08	**1·00**	0·97	1·03	**1·07**	1·06	1·08	**0·99**	0·98	1·01
2004	***1·00***	*1·00*	*1·00*	***1·00***	*1·00*	*1·00*	***1·00***	*1·00*	*1·00*	***1·00***	*1·00*	*1·00*	***1·00***	*1·00*	*1·00*	***1·00***	*1·00*	*1·00*
2009	**1·05**	0·91	1·20	**0·99**	0·96	1·02	**1·06**	0·98	1·15	**1·04**	1·01	1·07	**0·99**	0·98	0·99	**1·06**	1·04	1·08
2014	**1·00**	0·87	1·15	**0·94**	0·90	0·97	**1·02**	0·94	1·11	**0·99**	0·96	1·02	**0·93**	0·92	0·94	**1·02**	1·01	1·04
2019	**0·96**	0·84	1·10	**0·89**	0·86	0·92	**1·01**	0·93	1·09	**0·96**	0·94	0·99	**0·88**	0·87	0·89	**1·01**	0·99	1·03
**Cohort**	**RR**	**95%UI**		**RR**	**95%UI**		**RR**	**95%UI**		**RR**	**95%UI**		**RR**	**95%UI**		**RR**	**95%UI**	
1914	**0·77**	0·58	1·03	**0·61**	0·57	0·65	**0·85**	0·69	1·04	**0·79**	0·73	0·85	**0·62**	0·61	0·64	**0·86**	0·82	0·91
1919	**0·82**	0·66	1·01	**0·71**	0·68	0·75	**0·90**	0·78	1·04	**0·83**	0·78	0·87	**0·73**	0·72	0·73	**0·91**	0·87	0·94
1924	**0·91**	0·76	1·09	**0·83**	0·80	0·87	**0·95**	0·84	1·07	**0·92**	0·88	0·96	**0·84**	0·83	0·85	**0·95**	0·92	0·98
1929	**1·00**	0·85	1·17	**0·94**	0·91	0·98	**0·99**	0·89	1·11	**0·99**	0·95	1·03	**0·95**	0·94	0·96	**0·99**	0·96	1·01
1934	***1·00***	*1·00*	*1·00*	***1·00***	*1·00*	*1·00*	***1·00***	*1·00*	*1·00*	***1·00***	*1·00*	*1·00*	***1·00***	*1·00*	*1·00*	***1·00***	*1·00*	*1·00*
1939	**0·97**	0·84	1·12	**0·98**	0·95	1·01	**0·98**	0·89	1·08	**0·97**	0·94	1·01	**0·98**	0·98	0·99	**0·99**	0·97	1·01
1944	**0·97**	0·83	1·14	**0·94**	0·91	0·98	**0·98**	0·89	1·08	**0·98**	0·94	1·01	**0·95**	0·94	0·96	**0·98**	0·96	1·00
1949	**0·97**	0·83	1·14	**0·93**	0·89	0·97	**0·99**	0·89	1·09	**0·98**	0·94	1·01	**0·94**	0·93	0·95	**0·99**	0·97	1·01
1954	**1·02**	0·87	1·19	**0·95**	0·91	0·99	**1·00**	0·91	1·10	**1·02**	0·99	1·06	**0·96**	0·95	0·97	**1·00**	0·98	1·02
1959	**0·95**	0·81	1·11	**0·87**	0·83	0·91	**0·95**	0·87	1·04	**0·96**	0·92	0·99	**0·88**	0·87	0·88	**0·96**	0·94	0·98
1964	**0·82**	0·68	0·99	**0·72**	0·68	0·76	**0·90**	0·82	1·00	**0·83**	0·80	0·86	**0·73**	0·73	0·74	**0·90**	0·88	0·92
1969	**0·71**	0·52	0·96	**0·61**	0·55	0·68	**0·85**	0·74	0·98	**0·71**	0·67	0·75	**0·62**	0·61	0·63	**0·85**	0·83	0·88
**Southern Latin America**																		
	**Mortality**									**DALY**								
	**Laryngeal**			**Lung**			**Mesothelioma**			**Laryngeal**			**Lung**			**Mesothelioma**		
**Age-group**	**MR**	**95%UI**		**MR**	**95%UI**		**MR**	**95%UI**		**DR**	**95%UI**		**DR**	**95%UI**		**DR**	**95%UI**	
50-54	**0·12**	0·09	0·17	**2·92**	2·72	3·12	**0·89**	0·76	1·03	**4·69**	4·41	4·98	**110·76**	109·45	112·09	**33·62**	32·64	34·62

55-59	**0·35**	0·27	0·45	**8·09**	7·70	8·50	**1·50**	1·31	1·72	**11·96**	11·38	12·57	**269·74**	267·17	272·34	**49·95**	48·57	51·36
60-64	**0·55**	0·44	0·69	**13·98**	13·40	14·59	**1·73**	1·51	1·97	**16·28**	15·55	17·04	**402·99**	399·51	406·50	**49·59**	48·24	50·98
65-69	**1·02**	0·83	1·26	**28·75**	27·68	29·86	**2·58**	2·26	2·94	**25·42**	24·33	26·56	**698·69**	693·00	704·43	**62·39**	60·68	64·15
70-74	**1·58**	1·29	1·93	**47·25**	45·56	49·00	**3·45**	3·03	3·92	**32·21**	30·82	33·66	**944·76**	937·09	952·49	**68·69**	66·76	70·67
75-79	**2·81**	2·31	3·42	**84·94**	81·99	88·01	**5·61**	4·95	6·36	**46·01**	44·00	48·12	**1359·20**	1348·10	1370·39	**89·80**	87·24	92·43
80 plus	**3·78**	3·11	4·59	**108·70**	104·91	112·62	**7·02**	6·23	7·92	**43·74**	41·77	45·81	**1231·46**	1221·05	1241·96	**79·58**	77·29	81·94
**Period**	**RR**	**95%UI**		**RR**	**95%UI**		**RR**	**95%UI**		**RR**	**95%UI**		**RR**	**95%UI**		**RR**	**95%UI**	
1994	**0·96**	0·78	1·19	**0·93**	0·89	0·96	**0·70**	0·61	0·79	**0·97**	0·93	1·01	**0·92**	0·91	0·93	**0·70**	0·68	0·72
1999	**0·99**	0·82	1·18	**0·96**	0·93	0·99	**0·85**	0·75	0·95	**0·99**	0·95	1·03	**0·95**	0·95	0·96	**0·85**	0·83	0·87
2004	***1·00***	*1·00*	*1·00*	***1·00***	*1·00*	*1·00*	***1·00***	*1·00*	*1·00*	***1·00***	*1·00*	*1·00*	***1·00***	*1·00*	*1·00*	***1·00***	*1·00*	*1·00*
2009	**0·83**	0·70	0·99	**0·90**	0·87	0·93	**0·96**	0·87	1·07	**0·82**	0·79	0·86	**0·90**	0·89	0·90	**0·96**	0·94	0·98
2014	**0·74**	0·62	0·88	**0·82**	0·79	0·84	**0·96**	0·87	1·07	**0·73**	0·70	0·75	**0·80**	0·80	0·81	**0·95**	0·93	0·97
2019	**0·60**	0·50	0·72	**0·71**	0·68	0·73	**0·89**	0·81	0·98	**0·60**	0·58	0·63	**0·70**	0·70	0·71	**0·90**	0·88	0·92
**Cohort**	**RR**	**95%UI**		**RR**	**95%UI**		**RR**	**95%UI**		**RR**	**95%UI**		**RR**	**95%UI**		**RR**	**95%UI**	
1914	**0·64**	0·44	0·94	**0·73**	0·68	0·78	**0·93**	0·72	1·19	**0·65**	0·59	0·72	**0·75**	0·73	0·76	**0·94**	0·88	1·01
1919	**0·72**	0·55	0·94	**0·78**	0·75	0·82	**0·92**	0·78	1·10	**0·73**	0·68	0·78	**0·80**	0·79	0·81	**0·94**	0·90	0·99
1924	**0·83**	0·67	1·02	**0·87**	0·84	0·90	**0·98**	0·85	1·13	**0·84**	0·80	0·89	**0·89**	0·88	0·90	**1·00**	0·97	1·04
1929	**0·92**	0·76	1·11	**0·94**	0·91	0·98	**0·98**	0·86	1·11	**0·92**	0·88	0·97	**0·95**	0·95	0·96	**0·99**	0·96	1·02
1934	***1·00***	*1·00*	*1·00*	***1·00***	*1·00*	*1·00*	***1·00***	*1·00*	*1·00*	***1·00***	*1·00*	*1·00*	***1·00***	*1·00*	*1·00*	***1·00***	*1·00*	*1·00*
1939	**1·01**	0·85	1·21	**1·04**	1·00	1·07	**1·00**	0·90	1·12	**1·04**	1·00	1·08	**1·05**	1·04	1·06	**1·02**	1·00	1·05
1944	**1·05**	0·87	1·27	**1·07**	1·04	1·11	**1·03**	0·92	1·15	**1·08**	1·04	1·13	**1·10**	1·09	1·11	**1·06**	1·03	1·08
1949	**1·06**	0·86	1·31	**1·10**	1·06	1·14	**1·05**	0·93	1·18	**1·08**	1·03	1·12	**1·12**	1·11	1·13	**1·07**	1·05	1·10
1954	**0·96**	0·75	1·22	**1·05**	1·00	1·10	**1·00**	0·89	1·13	**0·97**	0·92	1·02	**1·06**	1·05	1·07	**1·02**	1·00	1·05
1959	**0·83**	0·61	1·13	**0·90**	0·85	0·95	**0·97**	0·85	1·11	**0·84**	0·79	0·89	**0·91**	0·90	0·92	**0·98**	0·95	1·01
1964	**0·67**	0·43	1·05	**0·69**	0·63	0·75	**0·91**	0·77	1·07	**0·68**	0·63	0·74	**0·70**	0·69	0·71	**0·92**	0·89	0·95
1969	**0·59**	0·24	1·48	**0·60**	0·50	0·71	**0·85**	0·65	1·11	**0·59**	0·51	0·69	**0·61**	0·59	0·63	**0·86**	0·82	0·90

Table S7. Results from the age-period-cohort analysis of mortality and disability-adjusted life years (DALY) age-specific groups rates for each cancer in men attributable to occupational asbestos exposure in the Americas regions from 1994 to 2023.

MR: mortality age-specific groups rates; DR: DALY age-specific groups rates; RR: rate ratio. Lung: trachea, bronchi and lungs cancer; 95%UI: uncertainty interval 95% (significant: 95%UI excluding one). Reference categories for period and cohort are highlighted in blue italics.

Elaborated by the authors (2025).

High-income North America																		
	Mortality									DALY								
	Ovarian			Lung			Mesothelioma			Ovarian			Lung			Mesothelioma		
**Age-group**	**MR**	**95%UI**		**MR**	**95%UI**		**MR**	**95%UI**		**DR**	**95%UI**		**DR**	**95%UI**		**DR**	**95%UI**	
50-54	**0·09**	0·07	0·10	**0·33**	0·30	0·35	**0·20**	0·18	0·22	**3·36**	3·29	3·44	**12·53**	12·39	12·68	**7·66**	7·53	7·80
55-59	**0·25**	0·23	0·27	**1·10**	1·05	1·15	**0·39**	0·36	0·42	**8·63**	8·48	8·78	**37·06**	36·76	37·37	**13·13**	12·92	13·34
60-64	**0·55**	0·51	0·59	**2·81**	2·72	2·90	**0·59**	0·54	0·63	**16·42**	16·19	16·66	**82·25**	81·73	82·78	**17·18**	16·92	17·44
65-69	**1·28**	1·21	1·36	**7·28**	7·10	7·47	**1·01**	0·94	1·08	**32·44**	32·03	32·85	**180·13**	179·15	181·11	**24·97**	24·60	25·35
70-74	**2·41**	2·28	2·54	**14·67**	14·34	15·00	**1·52**	1·42	1·63	**49·91**	49·30	50·53	**298·28**	296·75	299·82	**31·01**	30·55	31·48
75-79	**4·30**	4·08	4·53	**26·62**	26·04	27·21	**2·30**	2·16	2·45	**70·88**	70·03	71·73	**431·08**	428·90	433·28	**37·38**	36·82	37·93
80 plus	**6·91**	6·56	7·27	**38·23**	37·36	39·12	**3·19**	3·01	3·39	**77·51**	76·61	78·43	**424·97**	422·78	427·17	**35·31**	34·82	35·81
**Period**	**RR**	**95%UI**		**RR**	**95%UI**		**RR**	**95%UI**		**RR**	**95%UI**		**RR**	**95%UI**		**RR**	**95%UI**	
1994	**1·24**	1·17	1·32	**1·30**	1·27	1·33	**1·19**	1·12	1·27	**1·21**	1·20	1·23	**1·26**	1·26	1·27	**1·16**	1·14	1·17
1999	**1·12**	1·06	1·17	**1·13**	1·11	1·16	**1·05**	0·99	1·11	**1·11**	1·10	1·13	**1·13**	1·12	1·13	**1·04**	1·03	1·05
2004	***1·00***	*1·00*	*1·00*	***1·00***	*1·00*	*1·00*	***1·00***	*1·00*	*1·00*	***1·00***	*1·00*	*1·00*	***1·00***	*1·00*	*1·00*	***1·00***	*1·00*	*1·00*
2009	**0·90**	0·86	0·94	**0·91**	0·89	0·93	**1·00**	0·95	1·06	**0·88**	0·87	0·89	**0·90**	0·89	0·90	**0·99**	0·98	1·00
2014	**0·79**	0·75	0·83	**0·81**	0·79	0·82	**0·95**	0·90	1·00	**0·78**	0·77	0·79	**0·79**	0·79	0·80	**0·93**	0·92	0·95
2019	**0·72**	0·68	0·75	**0·73**	0·71	0·75	**0·95**	0·90	1·00	**0·70**	0·70	0·71	**0·72**	0·71	0·72	**0·93**	0·92	0·94
**Cohort**	**RR**	**95%UI**		**RR**	**95%UI**		**RR**	**95%UI**		**RR**	**95%UI**		**RR**	**95%UI**		**RR**	**95%UI**	
1914	**0·59**	0·54	0·65	**0·43**	0·41	0·44	**0·63**	0·57	0·69	**0·62**	0·61	0·63	**0·45**	0·45	0·45	**0·66**	0·64	0·68
1919	**0·75**	0·70	0·80	**0·60**	0·58	0·62	**0·77**	0·71	0·83	**0·77**	0·76	0·78	**0·62**	0·62	0·63	**0·80**	0·78	0·81
1924	**0·89**	0·84	0·94	**0·78**	0·76	0·80	**0·88**	0·83	0·94	**0·90**	0·88	0·91	**0·79**	0·78	0·79	**0·90**	0·88	0·91
1929	**0·96**	0·92	1·01	**0·91**	0·90	0·93	**0·93**	0·88	0·99	**0·95**	0·94	0·97	**0·91**	0·91	0·92	**0·94**	0·93	0·95
1934	***1·00***	*1·00*	*1·00*	***1·00***	*1·00*	*1·00*	***1·00***	*1·00*	*1·00*	***1·00***	*1·00*	*1·00*	***1·00***	*1·00*	*1·00*	***1·00***	*1·00*	*1·00*
1939	**0·99**	0·94	1·04	**0·99**	0·97	1·01	**1·01**	0·95	1·06	**1·00**	0·99	1·01	**1·00**	1·00	1·01	**1·01**	1·00	1·03
1944	**0·95**	0·90	1·01	**0·92**	0·90	0·94	**0·97**	0·91	1·03	**0·97**	0·96	0·98	**0·94**	0·93	0·94	**0·98**	0·97	0·99
1949	**0·90**	0·84	0·96	**0·82**	0·80	0·85	**0·90**	0·85	0·96	**0·91**	0·90	0·92	**0·83**	0·83	0·84	**0·92**	0·90	0·93
1954	**0·88**	0·81	0·95	**0·80**	0·77	0·83	**0·87**	0·81	0·94	**0·89**	0·87	0·90	**0·81**	0·80	0·82	**0·89**	0·87	0·90
1959	**0·85**	0·77	0·95	**0·81**	0·77	0·85	**0·85**	0·78	0·93	**0·86**	0·85	0·88	**0·82**	0·82	0·83	**0·87**	0·85	0·88
1964	**0·81**	0·69	0·95	**0·71**	0·65	0·77	**0·82**	0·73	0·92	**0·82**	0·80	0·84	**0·72**	0·71	0·73	**0·84**	0·82	0·85
1969	**0·78**	0·55	1·11	**0·53**	0·43	0·65	**0·81**	0·66	0·99	**0·80**	0·75	0·84	**0·54**	0·53	0·56	**0·83**	0·80	0·86

Central Latin America																		
	Mortality									DALY								
	Ovarian			Lung			Mesothelioma			Ovarian			Lung			Mesothelioma		
**Age-group**	**MR**	**95%UI**		**MR**	**95%UI**		**MR**	**95%UI**		**DR**	**95%UI**		**DR**	**95%UI**		**DR**	**95%UI**	
50-54	**0·07**	0·06	0·09	**0·08**	0·06	0·09	**0·20**	0·16	0·24	**2·82**	2·68	2·96	**2·90**	2·78	3·02	**7·61**	7·33	7·90
55-59	**0·15**	0·12	0·19	**0·19**	0·16	0·22	**0·34**	0·29	0·40	**5·13**	4·90	5·37	**6·37**	6·16	6·59	**11·46**	11·06	11·87
60-64	**0·23**	0·18	0·28	**0·38**	0·33	0·44	**0·41**	0·35	0·49	**6·70**	6·42	7·00	**11·07**	10·74	11·41	**12·04**	11·63	12·46
65-69	**0·37**	0·31	0·46	**0·76**	0·67	0·87	**0·56**	0·48	0·66	**9·27**	8·88	9·68	**18·73**	18·19	19·29	**13·82**	13·34	14·32
70-74	**0·60**	0·50	0·73	**1·53**	1·35	1·74	**0·87**	0·74	1·02	**12·26**	11·74	12·80	**30·95**	30·09	31·83	**17·58**	16·97	18·22

75-79	**0·77**	0·64	0·94	**2·31**	2·04	2·61	**0·98**	0·83	1·15	**12·57**	12·02	13·15	**37·31**	36·26	38·39	**15·81**	15·22	16·43
80 plus	**0·98**	0·82	1·18	**3·66**	3·26	4·11	**1·08**	0·92	1·27	**10·93**	10·47	11·42	**40·11**	39·01	41·23	**12·01**	11·55	12·49
**Period**	**RR**	**95%UI**		**RR**	**95%UI**		**RR**	**95%UI**		**RR**	**95%UI**		**RR**	**95%UI**		**RR**	**95%UI**	
1994	**0·81**	0·65	1·01	**1·10**	0·96	1·26	**0·88**	0·75	1·04	**0·82**	0·79	0·86	**1·11**	1·07	1·14	**0·88**	0·85	0·91
1999	**0·85**	0·70	1·03	**0·98**	0·86	1·10	**0·88**	0·75	1·02	**0·86**	0·82	0·89	**0·98**	0·96	1·01	**0·89**	0·86	0·91
2004	***1·00***	*1·00*	*1·00*	***1·00***	*1·00*	*1·00*	***1·00***	*1·00*	*1·00*	***1·00***	*1·00*	*1·00*	***1·00***	*1·00*	*1·00*	***1·00***	*1·00*	*1·00*
2009	**1·03**	0·87	1·21	**0·93**	0·83	1·03	**1·01**	0·89	1·15	**1·03**	1·00	1·07	**0·93**	0·91	0·96	**1·02**	0·99	1·04
2014	**1·03**	0·88	1·20	**0·86**	0·77	0·96	**0·90**	0·79	1·02	**1·04**	1·01	1·08	**0·87**	0·85	0·89	**0·91**	0·88	0·93
2019	**1·00**	0·86	1·17	**0·78**	0·70	0·87	**0·85**	0·74	0·96	**1·01**	0·98	1·05	**0·78**	0·77	0·80	**0·84**	0·82	0·87
**Cohort**	**RR**	**95%UI**		**RR**	**95%UI**		**RR**	**95%UI**		**RR**	**95%UI**		**RR**	**95%UI**		**RR**	**95%UI**	
1914	**1·19**	0·82	1·73	**1·02**	0·83	1·26	**0·93**	0·66	1·33	**1·17**	1·06	1·29	**1·00**	0·95	1·06	**0·94**	0·85	1·04
1919	**1·02**	0·78	1·35	**0·91**	0·77	1·06	**0·92**	0·72	1·19	**1·00**	0·93	1·07	**0·89**	0·86	0·93	**0·92**	0·87	0·98
1924	**1·02**	0·82	1·27	**0·94**	0·83	1·08	**0·99**	0·82	1·19	**1·02**	0·97	1·08	**0·94**	0·91	0·97	**0·99**	0·95	1·04
1929	**1·02**	0·84	1·23	**0·99**	0·89	1·11	**0·99**	0·84	1·17	**1·02**	0·97	1·06	**1·00**	0·97	1·02	**0·99**	0·95	1·03
1934	***1·00***	*1·00*	*1·00*	***1·00***	*1·00*	*1·00*	***1·00***	*1·00*	*1·00*	***1·00***	*1·00*	*1·00*	***1·00***	*1·00*	*1·00*	***1·00***	*1·00*	*1·00*
1939	**0·97**	0·82	1·15	**0·99**	0·89	1·10	**0·99**	0·86	1·14	**0·97**	0·93	1·01	**0·98**	0·96	1·01	**0·99**	0·96	1·02
1944	**0·95**	0·80	1·13	**0·96**	0·85	1·07	**0·97**	0·84	1·12	**0·94**	0·91	0·98	**0·94**	0·92	0·97	**0·96**	0·93	0·99
1949	**0·96**	0·80	1·15	**0·94**	0·83	1·07	**0·99**	0·85	1·14	**0·95**	0·92	0·99	**0·93**	0·91	0·96	**0·98**	0·95	1·02
1954	**0·99**	0·82	1·20	**0·96**	0·82	1·11	**0·99**	0·85	1·16	**0·99**	0·95	1·03	**0·95**	0·92	0·98	**0·99**	0·96	1·02
1959	**1·05**	0·85	1·28	**1·00**	0·84	1·19	**0·99**	0·84	1·17	**1·04**	1·00	1·08	**0·99**	0·96	1·03	**1·00**	0·96	1·03
1964	**1·09**	0·85	1·41	**1·00**	0·79	1·26	**0·97**	0·79	1·18	**1·08**	1·03	1·14	**0·99**	0·95	1·03	**0·97**	0·93	1·01
1969	**1·14**	0·77	1·71	**0·94**	0·60	1·45	**0·88**	0·65	1·19	**1·14**	1·06	1·22	**0·93**	0·87	1·00	**0·89**	0·84	0·94
**Caribbean**																		
	**Mortality**									**DALY**								
	**Ovarian**			**Lung**			**Mesothelioma**			**Ovarian**			**Lung**			**Mesothelioma**		
**Age-group**	**MR**	**95%UI**		**MR**	**95%UI**		**MR**	**95%UI**		**DR**	**95%UI**		**DR**	**95%UI**		**DR**	**95%UI**	
50-54	**0·06**	0·03	0·12	**0·13**	0·09	0·20	**0·20**	0·12	0·33	**2·30**	2·00	2·64	**5·02**	4·65	5·43	**7·64**	6·89	8·47
55-59	**0·12**	0·06	0·22	**0·32**	0·23	0·45	**0·29**	0·18	0·48	**4·01**	3·52	4·57	**10·65**	9·95	11·41	**9·92**	8·96	10·99
60-64	**0·07**	0·03	0·14	**0·27**	0·19	0·39	**0·13**	0·07	0·22	**2·07**	1·79	2·38	**7·83**	7·29	8·42	**3·66**	3·27	4·09
65-69	**0·10**	0·05	0·20	**0·38**	0·27	0·53	**0·19**	0·11	0·32	**2·44**	2·11	2·82	**9·18**	8·52	9·88	**4·57**	4·08	5·12
70-74	**0·24**	0·13	0·45	**1·02**	0·75	1·38	**0·29**	0·17	0·49	**4·95**	4·32	5·66	**20·50**	19·16	21·93	**5·85**	5·22	6·56
75-79	**0·18**	0·09	0·36	**0·94**	0·68	1·30	**0·28**	0·16	0·49	**2·98**	2·55	3·49	**15·10**	14·01	16·28	**4·49**	3·95	5·11
80 plus	**0·45**	0·26	0·79	**2·45**	1·87	3·23	**0·50**	0·31	0·80	**5·05**	4·42	5·77	**26·62**	24·95	28·41	**5·53**	4·93	6·21
**Period**	**RR**	**95%UI**		**RR**	**95%UI**		**RR**	**95%UI**		**RR**	**95%UI**		**RR**	**95%UI**		**RR**	**95%UI**	
1994	**0·96**	0·52	1·76	**0·89**	0·64	1·23	**1·04**	0·67	1·62	**0·96**	0·85	1·08	**0·88**	0·83	0·94	**1·05**	0·96	1·14
1999	**1·01**	0·58	1·78	**0·89**	0·66	1·19	**1·02**	0·68	1·55	**1·02**	0·91	1·14	**0·90**	0·85	0·96	**1·03**	0·95	1·11
2004	***1·00***	*1·00*	*1·00*	***1·00***	*1·00*	*1·00*	***1·00***	*1·00*	*1·00*	***1·00***	*1·00*	*1·00*	***1·00***	*1·00*	*1·00*	***1·00***	*1·00*	*1·00*
2009	**0·95**	0·56	1·62	**0·95**	0·73	1·23	**0·92**	0·62	1·37	**0·95**	0·86	1·06	**0·94**	0·89	1·00	**0·92**	0·85	1·00
2014	**0·96**	0·58	1·60	**0·92**	0·72	1·19	**0·96**	0·65	1·42	**0·96**	0·87	1·07	**0·91**	0·87	0·97	**0·96**	0·89	1·04
2019	**1·02**	0·63	1·68	**0·92**	0·72	1·18	**0·93**	0·62	1·38	**1·02**	0·92	1·14	**0·93**	0·88	0·98	**0·93**	0·86	1·02

Cohort	RR	95%UI		RR	95%UI		RR	95%UI		RR	95%UI		RR	95%UI		RR	95%UI	
1914	1·04	0·40	2·72	0·94	0·58	1·51	0·93	0·40	2·16	1·07	0·83	1·37	0·97	0·86	1·10	0·95	0·76	1·19
1919	1·02	0·47	2·22	0·91	0·62	1·34	0·96	0·49	1·88	1·03	0·84	1·25	0·92	0·84	1·02	0·97	0·81	1·15
1924	1·02	0·53	1·95	0·94	0·69	1·29	0·98	0·55	1·73	1·02	0·87	1·19	0·94	0·87	1·02	0·98	0·85	1·12
1929	0·99	0·53	1·85	0·99	0·74	1·34	1·00	0·59	1·70	0·99	0·85	1·15	0·99	0·92	1·07	1·00	0·88	1·13
1934	1·00	1·00	1·00	1·00	1·00	1·00	1·00	1·00	1·00	1·00	1·00	1·00	1·00	1·00	1·00	1·00	1·00	1·00
1939	0·97	0·55	1·69	1·01	0·77	1·32	0·97	0·61	1·55	0·97	0·85	1·10	1·01	0·95	1·08	0·97	0·88	1·08
1944	0·96	0·54	1·71	1·06	0·79	1·40	0·98	0·62	1·55	0·97	0·85	1·10	1·07	1·00	1·14	0·98	0·89	1·09
1949	0·98	0·55	1·75	1·10	0·82	1·48	0·97	0·62	1·53	0·98	0·87	1·11	1·11	1·04	1·18	0·98	0·88	1·07
1954	1·00	0·54	1·84	1·08	0·78	1·49	0·98	0·62	1·56	1·00	0·88	1·14	1·08	1·01	1·16	0·99	0·90	1·09
1959	1·02	0·55	1·91	0·96	0·68	1·38	0·98	0·62	1·56	1·03	0·91	1·17	0·97	0·91	1·05	0·99	0·89	1·09
1964	1·02	0·51	2·04	0·86	0·56	1·32	0·98	0·59	1·61	1·03	0·89	1·18	0·86	0·80	0·94	0·98	0·88	1·09
1969	1·03	0·35	3·03	0·73	0·33	1·60	0·97	0·48	1·97	1·04	0·85	1·27	0·73	0·64	0·84	0·96	0·84	1·11
Andean Latin America																		
	Mortality									DALY								
	Ovarian			Lung			Mesothelioma			Ovarian			Lung			Mesothelioma		
Age-group	MR	95%UI		MR	95%UI		MR	95%UI		DR	95%UI		DR	95%UI		DR	95%UI	
50-54	0·06	0·03	0·10	0·09	0·06	0·14	0·18	0·12	0·26	2·22	1·98	2·48	3·37	3·12	3·64	6·80	6·31	7·34
55-59	0·13	0·08	0·21	0·21	0·15	0·30	0·28	0·20	0·40	4·33	3·93	4·78	7·19	6·74	7·66	9·45	8·79	10·16
60-64	0·19	0·12	0·31	0·45	0·34	0·60	0·49	0·35	0·67	5·69	5·18	6·25	13·20	12·48	13·95	14·10	13·18	15·08
65-69	0·29	0·19	0·45	0·79	0·61	1·02	0·47	0·33	0·66	7·18	6·53	7·90	19·37	18·33	20·47	11·38	10·58	12·25
70-74	0·51	0·33	0·77	1·62	1·28	2·06	0·70	0·50	0·99	10·31	9·39	11·33	32·80	31·10	34·58	14·16	13·14	15·27
75-79	0·81	0·54	1·21	3·25	2·62	4·05	1·12	0·80	1·56	13·16	11·98	14·45	52·59	49·97	55·35	18·01	16·69	19·43
80 plus	1·30	0·90	1·89	5·94	4·84	7·30	1·37	1·01	1·87	14·04	12·85	15·34	63·51	60·49	66·68	15·21	14·13	16·38
Period	RR	95%UI		RR	95%UI		RR	95%UI		RR	95%UI		RR	95%UI		RR	95%UI	
1994	0·91	0·58	1·44	1·35	1·06	1·72	1·24	0·91	1·70	0·92	0·84	1·01	1·37	1·30	1·44	1·25	1·17	1·33
1999	0·92	0·62	1·37	0·99	0·79	1·23	1·19	0·89	1·59	0·92	0·85	1·01	0·99	0·95	1·04	1·19	1·12	1·27
2004	1·00	1·00	1·00	1·00	1·00	1·00	1·00	1·00	1·00	1·00	1·00	1·00	1·00	1·00	1·00	1·00	1·00	1·00
2009	0·95	0·67	1·35	0·91	0·75	1·10	0·89	0·67	1·17	0·95	0·88	1·03	0·91	0·87	0·95	0·89	0·84	0·94
2014	0·95	0·68	1·34	0·85	0·70	1·03	0·89	0·69	1·17	0·95	0·88	1·02	0·85	0·81	0·89	0·89	0·84	0·94
2019	1·06	0·77	1·46	0·90	0·74	1·09	1·04	0·81	1·34	1·06	0·99	1·14	0·91	0·87	0·95	1·05	0·99	1·10
Cohort	RR	95%UI		RR	95%UI		RR	95%UI		RR	95%UI		RR	95%UI		RR	95%UI	
1914	0·89	0·43	1·80	0·88	0·63	1·24	0·99	0·58	1·70	0·91	0·75	1·09	0·89	0·82	0·97	1·00	0·86	1·15
1919	0·99	0·58	1·66	0·96	0·74	1·26	0·98	0·65	1·48	0·99	0·87	1·13	0·96	0·90	1·02	0·98	0·89	1·09
1924	0·99	0·64	1·52	0·99	0·79	1·24	0·96	0·67	1·37	0·99	0·89	1·10	0·98	0·93	1·04	0·96	0·88	1·05
1929	0·98	0·67	1·44	1·00	0·82	1·22	0·98	0·71	1·35	0·98	0·90	1·08	1·00	0·96	1·05	0·98	0·91	1·06
1934	1·00	1·00	1·00	1·00	1·00	1·00	1·00	1·00	1·00	1·00	1·00	1·00	1·00	1·00	1·00	1·00	1·00	1·00
1939	0·96	0·69	1·35	0·96	0·80	1·16	0·98	0·74	1·29	0·97	0·90	1·05	0·97	0·92	1·01	0·98	0·92	1·05
1944	0·99	0·68	1·42	1·00	0·81	1·24	0·99	0·74	1·33	0·99	0·91	1·08	1·00	0·95	1·05	1·00	0·94	1·06
1949	1·02	0·69	1·49	1·03	0·81	1·30	1·01	0·75	1·35	1·02	0·94	1·10	1·02	0·97	1·08	1·01	0·95	1·08

1954	**0·98**	0·65	1·49	**0·98**	0·75	1·29	**0·99**	0·73	1·33	**0·98**	0·90	1·07	**0·98**	0·93	1·04	**0·99**	0·93	1·05
1959	**0·94**	0·59	1·50	**0·94**	0·68	1·30	**0·97**	0·70	1·34	**0·95**	0·86	1·04	**0·94**	0·88	1·00	**0·97**	0·91	1·04
1964	**0·97**	0·54	1·74	**0·97**	0·62	1·52	**0·97**	0·65	1·44	**0·97**	0·87	1·09	**0·97**	0·89	1·05	**0·97**	0·90	1·05
1969	**0·97**	0·37	2·55	**0·95**	0·43	2·10	**0·96**	0·54	1·73	**0·97**	0·82	1·15	**0·94**	0·82	1·07	**0·96**	0·86	1·07
**Tropical LatinAmerica**																		
	**Mortality**									**DALY**								
	**Ovarian**			**Lung**			**Mesothelioma**			**Ovarian**			**Lung**			**Mesothelioma**		
**Age-group**	**MR**	**95%UI**		**MR**	**95%UI**		**MR**	**95%UI**		**DR**	**95%UI**		**DR**	**95%UI**		**DR**	**95%UI**	
50-54	**0·13**	0·10	0·15	**0·28**	0·24	0·31	**0·47**	0·42	0·54	**4·91**	4·73	5·09	**10·51**	10·28	10·74	**18·13**	17·67	18·60
55-59	**0·27**	0·23	0·32	**0·66**	0·60	0·72	**0·75**	0·66	0·84	**9·26**	8·96	9·58	**21·97**	21·55	22·40	**25·12**	24·50	25·76
60-64	**0·20**	0·17	0·24	**0·56**	0·51	0·62	**0·41**	0·36	0·47	**5·87**	5·65	6·09	**16·26**	15·93	16·60	**11·87**	11·54	12·21
65-69	**0·42**	0·36	0·49	**1·29**	1·18	1·41	**0·69**	0·60	0·78	**10·43**	10·08	10·80	**31·49**	30·89	32·11	**16·83**	16·36	17·30
70-74	**0·72**	0·62	0·84	**2·34**	2·15	2·54	**0·96**	0·84	1·09	**14·65**	14·15	15·16	**46·95**	46·07	47·85	**19·33**	18·78	19·89
75-79	**1·95**	1·70	2·23	**6·73**	6·25	7·25	**2·42**	2·16	2·71	**31·71**	30·73	32·72	**108·09**	106·26	109·96	**39·08**	38·07	40·12
80 plus	**3·79**	3·34	4·29	**13·76**	12·85	14·73	**4·21**	3·79	4·67	**41·12**	39·93	42·35	**146·67**	144·34	149·04	**45·56**	44·46	46·70
**Period**	**RR**	**95%UI**		**RR**	**95%UI**		**RR**	**95%UI**		**RR**	**95%UI**		**RR**	**95%UI**		**RR**	**95%UI**	
1994	**0·98**	0·84	1·14	**0·88**	0·80	0·97	**0·95**	0·84	1·07	**0·98**	0·95	1·01	**0·88**	0·86	0·90	**0·94**	0·92	0·96
1999	**0·99**	0·87	1·13	**0·92**	0·86	1·00	**0·98**	0·89	1·09	**1·00**	0·97	1·03	**0·93**	0·91	0·95	**0·99**	0·97	1·01
2004	***1·00***	*1·00*	*1·00*	***1·00***	*1·00*	*1·00*	***1·00***	*1·00*	*1·00*	***1·00***	*1·00*	*1·00*	***1·00***	*1·00*	*1·00*	***1·00***	*1·00*	*1·00*
2009	**0·99**	0·88	1·11	**1·08**	1·01	1·15	**1·00**	0·91	1·10	**0·99**	0·97	1·02	**1·09**	1·07	1·10	**1·00**	0·99	1·02
2014	**0·97**	0·87	1·10	**1·15**	1·08	1·23	**0·98**	0·90	1·08	**0·98**	0·95	1·00	**1·15**	1·13	1·17	**0·98**	0·96	1·00
2019	**0·95**	0·85	1·07	**1·18**	1·11	1·25	**0·93**	0·85	1·02	**0·95**	0·93	0·98	**1·17**	1·16	1·19	**0·92**	0·90	0·94
**Cohort**	**RR**	**95%UI**		**RR**	**95%UI**		**RR**	**95%UI**		**RR**	**95%UI**		**RR**	**95%UI**		**RR**	**95%UI**	
1914	**0·94**	0·76	1·17	**0·94**	0·83	1·07	**0·97**	0·81	1·17	**0·97**	0·91	1·02	**0·98**	0·95	1·01	**1·01**	0·96	1·06
1919	**0·97**	0·82	1·14	**0·96**	0·88	1·06	**0·99**	0·86	1·13	**0·98**	0·94	1·02	**0·98**	0·95	1·00	**1·01**	0·97	1·04
1924	**0·96**	0·84	1·11	**0·95**	0·88	1·02	**0·96**	0·85	1·08	**0·97**	0·93	1·00	**0·95**	0·94	0·97	**0·97**	0·94	1·00
1929	**1·01**	0·90	1·14	**0·98**	0·91	1·04	**0·99**	0·89	1·10	**1·01**	0·98	1·04	**0·98**	0·96	1·00	**0·99**	0·96	1·02
1934	***1·00***	*1·00*	*1·00*	***1·00***	*1·00*	*1·00*	***1·00***	*1·00*	*1·00*	***1·00***	*1·00*	*1·00*	***1·00***	*1·00*	*1·00*	***1·00***	*1·00*	*1·00*
1939	**0·98**	0·88	1·09	**1·01**	0·95	1·07	**0·98**	0·89	1·08	**0·98**	0·95	1·00	**1·01**	0·99	1·02	**0·98**	0·96	1·01
1944	**0·95**	0·83	1·08	**0·97**	0·90	1·04	**0·94**	0·84	1·04	**0·95**	0·93	0·98	**0·97**	0·96	0·99	**0·95**	0·92	0·97
1949	**0·94**	0·82	1·09	**0·98**	0·90	1·06	**0·95**	0·85	1·06	**0·95**	0·92	0·97	**0·98**	0·97	1·00	**0·96**	0·94	0·98
1954	**0·98**	0·85	1·14	**1·05**	0·96	1·14	**1·00**	0·90	1·12	**0·99**	0·96	1·02	**1·06**	1·04	1·07	**1·01**	0·99	1·03
1959	**1·00**	0·85	1·18	**1·07**	0·98	1·18	**1·02**	0·91	1·13	**1·01**	0·98	1·04	**1·08**	1·06	1·10	**1·03**	1·01	1·05
1964	**1·00**	0·83	1·20	**0·92**	0·82	1·03	**0·98**	0·87	1·11	**1·00**	0·97	1·04	**0·93**	0·91	0·95	**0·99**	0·97	1·02
1969	**0·96**	0·70	1·34	**0·74**	0·59	0·92	**0·94**	0·78	1·14	**0·97**	0·92	1·03	**0·75**	0·72	0·78	**0·96**	0·93	0·99
**Southern Latin America**																		
	**Mortality**									**DALY**								
	**Ovarian**			**Lung**			**Mesothelioma**			**Ovarian**			**Lung**			**Mesothelioma**		
**Age-group**	**MR**	**95%UI**		**MR**	**95%UI**		**MR**	**95%UI**		**DR**	**95%UI**		**DR**	**95%UI**		**DR**	**95%UI**	
50-54	**0·13**	0·10	0·18	**0·22**	0·18	0·27	**0·30**	0·24	0·38	**5·09**	4·82	5·37	**8·41**	8·10	8·73	**11·41**	10·91	11·93

55-59	**0·26**	0·20	0·33	**0·46**	0·39	0·54	**0·44**	0·36	0·54	**8·78**	8·37	9·20	**15·32**	14·85	15·82	**14·88**	14·27	15·52
60-64	**0·51**	0·42	0·63	**1·06**	0·93	1·21	**0·74**	0·61	0·89	**15·03**	14·41	15·68	**30·69**	29·86	31·53	**21·49**	20·66	22·35
65-69	**1·04**	0·86	1·24	**2·33**	2·07	2·61	**1·10**	0·92	1·32	**25·59**	24·60	26·62	**56·60**	55·19	58·04	**26·95**	25·92	28·02
70-74	**1·64**	1·38	1·94	**3·82**	3·43	4·26	**1·46**	1·22	1·74	**33·26**	32·00	34·56	**76·69**	74·84	78·59	**29·43**	28·30	30·61
75-79	**2·25**	1·90	2·66	**5·67**	5·11	6·29	**1·71**	1·43	2·04	**36·35**	34·95	37·80	**90·83**	88·64	93·07	**27·56**	26·45	28·71
80 plus	**2·48**	2·10	2·92	**6·88**	6·22	7·61	**1·88**	1·59	2·22	**27·91**	26·84	29·02	**76·17**	74·37	78·02	**21·20**	20·37	22·07
**Period**	**RR**	**95%UI**		**RR**	**95%UI**		**RR**	**95%UI**		**RR**	**95%UI**		**RR**	**95%UI**		**RR**	**95%UI**	
1994	**0·62**	0·50	0·76	**0·54**	0·47	0·62	**0·54**	0·44	0·66	**0·62**	0·59	0·64	**0·53**	0·52	0·55	**0·54**	0·52	0·57
1999	**0·78**	0·66	0·92	**0·72**	0·64	0·80	**0·74**	0·63	0·88	**0·78**	0·75	0·81	**0·71**	0·69	0·73	**0·74**	0·71	0·77
2004	***1·00***	*1·00*	*1·00*	***1·00***	*1·00*	*1·00*	***1·00***	*1·00*	*1·00*	***1·00***	*1·00*	*1·00*	***1·00***	*1·00*	*1·00*	***1·00***	*1·00*	*1·00*
2009	**1·04**	0·90	1·21	**1·21**	1·10	1·33	**1·09**	0·94	1·26	**1·05**	1·02	1·09	**1·22**	1·19	1·24	**1·10**	1·07	1·14
2014	**1·04**	0·90	1·21	**1·31**	1·20	1·44	**1·22**	1·06	1·40	**1·04**	1·00	1·07	**1·31**	1·28	1·33	**1·22**	1·18	1·25
2019	**0·93**	0·81	1·08	**1·21**	1·10	1·32	**1·13**	0·98	1·30	**0·93**	0·90	0·96	**1·20**	1·17	1·22	**1·13**	1·10	1·17
**Cohort**	**RR**	**95%UI**		**RR**	**95%UI**		**RR**	**95%UI**		**RR**	**95%UI**		**RR**	**95%UI**		**RR**	**95%UI**	
1914	**0·95**	0·67	1·35	**1·12**	0·90	1·40	**1·01**	0·68	1·49	**0·98**	0·89	1·08	**1·16**	1·09	1·22	**1·01**	0·91	1·12
1919	**0·98**	0·77	1·24	**1·04**	0·89	1·21	**0·95**	0·73	1·24	**1·00**	0·94	1·06	**1·06**	1·02	1·10	**0·96**	0·89	1·02
1924	**1·02**	0·85	1·23	**1·03**	0·92	1·16	**1·01**	0·82	1·23	**1·03**	0·99	1·08	**1·05**	1·02	1·08	**1·01**	0·96	1·06
1929	**1·03**	0·87	1·20	**1·01**	0·92	1·12	**1·00**	0·84	1·19	**1·04**	1·00	1·08	**1·02**	1·00	1·05	**1·01**	0·96	1·05
1934	***1·00***	*1·00*	*1·00*	***1·00***	*1·00*	*1·00*	***1·00***	*1·00*	*1·00*	***1·00***	*1·00*	*1·00*	***1·00***	*1·00*	*1·00*	***1·00***	*1·00*	*1·00*
1939	**0·98**	0·85	1·14	**1·03**	0·95	1·13	**1·02**	0·87	1·18	**0·99**	0·95	1·02	**1·04**	1·01	1·06	**1·02**	0·98	1·05
1944	**1·00**	0·85	1·18	**1·11**	1·01	1·22	**1·02**	0·88	1·20	**1·02**	0·98	1·05	**1·12**	1·10	1·15	**1·03**	0·99	1·06
1949	**1·02**	0·85	1·21	**1·17**	1·05	1·30	**1·02**	0·87	1·20	**1·03**	0·99	1·07	**1·18**	1·16	1·21	**1·03**	0·99	1·06
1954	**0·99**	0·81	1·21	**1·18**	1·05	1·33	**1·03**	0·87	1·22	**1·01**	0·97	1·05	**1·20**	1·17	1·23	**1·04**	1·00	1·08
1959	**1·01**	0·80	1·29	**1·11**	0·95	1·29	**1·03**	0·85	1·25	**1·03**	0·98	1·07	**1·12**	1·09	1·16	**1·03**	0·99	1·07
1964	**0·99**	0·71	1·37	**0·91**	0·72	1·14	**0·95**	0·74	1·21	**1·00**	0·94	1·06	**0·92**	0·88	0·95	**0·95**	0·90	1·00
1969	**0·97**	0·55	1·72	**0·72**	0·47	1·12	**0·89**	0·60	1·31	**0·99**	0·89	1·08	**0·74**	0·69	0·79	**0·89**	0·83	0·95

Table S8. Results from the age-period-cohort analysis of mortality and disability-adjusted life years (DALY) age-specific groups rates for each cancer in women attributable to occupational asbestos exposure in the Americas regions from 1994 to 2023.

MR: mortality age-specific groups rates; DR: DALY age-specific groups rates; RR: rate ratio. Lung: trachea, bronchi and lungs cancer; 95%UI: uncertainty interval 95% (significant: 95%UI excluding one). Reference categories for period and cohort are highlighted in blue italics.

Elaborated by the authors (2025).
