# Supplementary material for: Burden of cancer attributable to occupational asbestos exposure in the Americas, 1990–2023: an analysis using the Global Burden of Disease Study 2023
Source: Lancet Reg Health Am. 2026 Apr 2;58:101463. doi: 10.1016/j.lana.2026.101463 (PMC13085095; doi:10.1016/j.lana.2026.101463)
Supplement: Appendix 1 [file mmc2.pdf]

APPENDIX 1

AUTHORS' CONTRIBUTIONS

Managing the overall research enterprise

Michael Brauer, Lisa Force, Simon I Hay, Katrin Burkart

Writing the first draft of the manuscript

Flavia Araujo Girardi, Michael Brauer, Deborah Carvalho Malta, Maria Teresa Bustamante-Teixeira, Mário Círio Nogueira, Maximiliano Ribeiro Guerra, Katrin Burkart

Primary responsibility for applying analytical methods to produce estimates

Flavia Araujo Girardi, Lisa Force, Sandra Spearman, Katrin Burkart

Primary responsibility for seeking, cataloguing, extracting, or cleaning data; designing or coding figures and tables

Sandra Spearman

Providing data or critical feedback on data sources

Kamoru Ademola Adedokun, Mustafa Alkhawam, Intima Alrimawi, Arushee Bhatnagar, Michael Brauer, Katrin Burkart, Maria Teresa Bustamante-Teixeira, Carlos A Castañeda-Orjuela, Vijay Kumar Chattu, Sunghyun Chung, Xiaochen Dai, Wendel Mombaque dos Santos, Ibrahim Farahat El Bayoumy, Abdelrahman Gamil Gad, Flavia Araujo Girardi, Simon I Hay, Muhammad Hamza Ilyas, Mohamed Jalloh, Armaan Jamal, Ramat T. Kamorudeen, Ibraheem M Karaye, Khaled Khatab, Farbod Khosravi, Adnan Kisa, Deborah Carvalho Malta, Lokesh Manjani, Ali H Mokdad, Seyed Mohamad Sadegh Mousavi Kiasary, Christopher J L Murray, Mahmoud Nassar, Mario Cirio Nogueira, Andrew T Olagunju, Atakan Orscelik, Parinaz Paranjkhoo, Neel Navinkumar Patel, Shrikant Pawar, Jagadeesh Puvvula, Maximiliano Ribeiro Guerra, Jefferson Antonio Buendia Rodriguez, Sharmistha Roy, Cameron John Sabet, Allen Seylani, Samendra P Sherchan, Jasvinder A Singh, Chen-Yang Su, Jabeen Taiba, Aliscia Vieira

Developing methods or computational machinery

Katrin Burkart, Xiaochen Dai, Flavia Araujo Girardi, Simon I Hay, Ali H Mokdad, Christopher J L Murray, Mario Cirio Nogueira, Sandra Spearman

Providing critical feedback on methods or results

Lisa C Adams, Kamoru Ademola Adedokun, Oluwatobi E Adegbile, Ali M Alfalki, Mustafa Alkhawam, Intima Alrimawi, Demelash Areda, Sina Azadnajafabad, Franca Barbic, Abiye Assefa Berihun, Arushee Bhatnagar, Alejandro Botero Carvajal, Michael Brauer, Katrin Burkart, Maria Teresa Bustamante-Teixeira, Carlos A Castañeda-Orjuela, Vijay Kumar Chattu, Sunghyun Chung, Xiaochen Dai, Wendel Mombaque dos Santos, Osamudiamen Ebohon, Ibrahim Farahat El Bayoumy, Elochukwu Ezenwankwo, Xiangning Fan, Abdelrahman Gamil Gad, Ali Gholamrezanezhad, Flavia Araujo Girardi, Simon I Hay, Muhammad Hamza Ilyas, Mohamed Jalloh, Armaan Jamal, Nathan T Jibat, Ramat T. Kamorudeen, Samuel Berchi Kankam, Ibraheem M Karaye, Khaled Khatab, Farbod Khosravi, Adnan Kisa, Deborah Carvalho Malta, Lokesh Manjani, Tomislav Mestrovic, Ali H Mokdad, Seyed Mohamad Sadegh Mousavi Kiasary, Christopher J L Murray, Mahmoud Nassar, Abigia Ashenafi Negash, Meti T Negassa, Mario Cirio

Nogueira, Andrew T Olagunju, Atakan Orscelik, Parinaz Paranjkhoo, Neel Navinkumar Patel, Shrikant Pawar, Farzad Pourghazi, Jagadeesh Puvvula, Mamunur Rashid, Maximiliano Ribeiro Guerra, Jefferson Antonio Buendia Rodriguez, Sharmistha Roy, Cameron John Sabet, Samendra P Sherchan, Jasvinder A Singh, Sandra Spearman, Sebastian Straube, Chen-Yang Su, Jabeen Taiba, Aliscia Vieira

Drafting the work or revising it critically for important intellectual content

Kamoru Ademola Adedokun, Oluwatobi E Adegbile, Ali M Alfalki, Mustafa Alkhawam, Intima Alrimawi, Sina Azadnajafabad, Arushee Bhatnagar, Alejandro Botero Carvajal, Michael Brauer, Katrin Burkart, Maria Teresa Bustamante-Teixeira, Carlos A Castañeda-Orjuela, Vijay Kumar Chattu, Sunghyun Chung, Wendel Mombaque dos Santos, Osamudiamen Ebohon, Ibrahim Farahat El Bayoumy, Elochukwu Ezenwankwo, Xiangning Fan, Lisa M Force, Flavia Araujo Girardi, Simon I Hay, Muhammad Hamza Ilyas, Mohamed Jalloh, Armaan Jamal, Arun Kamireddy, Ramat T. Kamorudeen, Samuel Berchi Kankam, Khaled Khatab, Adnan Kisa, Deborah Carvalho Malta, Lokesh Manjani, Tomislav Mestrovic, Ali H Mokdad, Christopher J L Murray, Nassar, Mario Cirio Nogueira, Andrew T Olagunju, Atakan Orscelik, Parinaz Paranjkhoo, Neel Navinkumar Patel, Shrikant Pawar, Jagadeesh Puvvula, Maximiliano Ribeiro Guerra, Jefferson Antonio Buendia Rodriguez, Sharmistha Roy, Cameron John Sabet, Allen Seylani, Samendra P Sherchan, Jasvinder A Singh, Sebastian Straube, Chen-Yang Su, Aliscia Vieira

Managing the estimation or publications process

Katrin Burkart, Flavia Araujo Girardi, Simon I Hay, Ali H Mokdad, Christopher J L Murray
