# Supplementary material for: Burden of cancer attributable to occupational asbestos exposure in the Americas, 1990–2023: an analysis using the Global Burden of Disease Study 2023
Source: Lancet Reg Health Am. 2026 Apr 2;58:101463. doi: 10.1016/j.lana.2026.101463 (PMC13085095; doi:10.1016/j.lana.2026.101463)
Supplement: PubMed [file mmc3.pdf]

GBD 2023 Americas Occupational Exposure Collaborators

Given Names	Last Name
Flavia Araujo*	Girardi [A]
Michael	Brauer [B, C]
Lisa M	Force [C, D, E]
Simon I	Hay [C, D]
Sandra	Spearman [C]
Deborah Carvalho	Malta [F]
Maria Teresa	Bustamante-Teixeira [G]
Mario Cirio	Nogueira [G]
Maximiliano	Ribeiro Guerra [G]
Lisa C	Adams [H, I]
Kamoru Ademola	Adedokun [J, K]
Oluwatobi E	Adegbile [L, M]
Ali M	Alfalki [N]
Mustafa	Alkhawam [O]
Intima	Alrimawi [P]
Demelash	Areda [Q]
Sina	Azadnajafabad [R]
Franca	Barbic [S, T]
Abiye Assefa	Berihun [U]
Arushee	Bhatnagar [V]
Alejandro	Botero Carvajal [W]
Carlos A	Castañeda-Orjuela [X, Y]

Given Names	Last Name
Vijay Kumar	Chattu [AA, Z]
Sunghyun	Chung [AB]
Xiaochen	Dai [C, D]
Wendel Mombaque	dos Santos [AC, AD]
Osamudiamen	Ebohon [AE, AF]
Ibrahim Farahat	El Bayoumy [AG, AH]
Elochukwu	Ezenwankwo [AI]
Xiangning	Fan [AJ]
Abdelrahman Gamil	Gad [AK]
Ali	Gholamrezanezhad [AL]
Muhammad Hamza	Ilyas [AM]
Mohamed	Jalloh [AN, AO]
Armaan	Jamal [AP]
Nathan T	Jibat [AQ]
Arun	Kamireddy [AK]
Ramat T.	Kamorudeen [AR, AS]
Samuel Berchi	Kankam [AT]
Ibraheem M	Karaye [AU, AV]
Khaled	Khatab [AW, AX]
Farbod	Khosravi [AY, AZ]
Adnan	Kisa [BA, BB]
Lokesh	Manjani [BC]
Tomislav	Mestrovic [BD, C]
Ali H	Mokdad [C, D]
Seyed Mohamad Sadegh	Mousavi Kiasary [BE, BF]
Christopher J L	Murray [C, D]

Given Names	Last Name
Mahmoud	Nassar [BG]
Abigia Ashenafi	Negash [BH, BI]
Meti T	Negassa [BJ]
Andrew T	Olagunju [BK, BL]
Atakan	Orscelik [BM]
Parinaz	Paranjkhoo [BN]
Neel Navinkumar	Patel [BO]
Shrikant	Pawar [BP]
Farzad	Pourghazi [BQ]
Jagadeesh	Puvvula [BR]
Mamunur	Rashid [BS]
Jefferson Antonio Buendia	Rodriguez [BT, BU]
Sharmistha	Roy [BV]
Cameron John	Sabet [BW]
Allen	Seylani [BX]
Samendra P	Sherchan [BY, BZ]
Jasvinder A	Singh [CA, CB]
Sebastian	Straube [CC, CD]
Chen-Yang	Su [CE]
Jabeen	Taiba [CF]
Aliscia	Vieira [CG]
Katrin**	Burkart [C, D]

*Lead author

**Senior author

Affiliations

[A] Public Health Department, Federal University of Juiz de Fora, Juiz de Fora, Brazil

[B] School of Population and Public Health, University of British Columbia, Vancouver, BC, Canada

[C] Institute for Health Metrics and Evaluation, University of Washington, Seattle, WA, USA

[D] Department of Health Metrics Sciences, School of Medicine, University of Washington, Seattle, WA, USA

[E] Division of Pediatric Hematology-Oncology, St. Jude Children's Research Hospital, Seattle, WA, USA

[F] Department of Maternal-Child Nursing and Public Health, Federal University of Minas Gerais, Belo Horizonte, Brazil

[G] Department of Public Health, Federal University of Juiz de Fora, Juiz de Fora, Brazil

[H] Department of Diagnostic and Interventional Radiology, Technical University of Munich, Munich, Germany

[I] Stanford University, Palo Alto, CA, USA

[J] Department of Immunology, Roswell Park Comprehensive Cancer Center, Buffalo, NY, USA

[K] Graduate Program Division, University at Buffalo, Buffalo, NY, USA

[L] Department of Pediatrics, East Tennessee State University, Johnson City, TN, USA

[M] Center for Cardiovascular Risk Research, Center for Cardiovascular Risk Research, Johnson City, TN, USA

[N] Department of Epidemiology and Biostatistics, University of South Carolina, Columbia, SC, USA

[O] Cardiovascular Division, University of Alabama, Birmingham, AL, USA

[P] Department of Nursing, Georgetown University, Washington, DC, USA

[Q] College of Art and Science, Ottawa University, Surprise, AZ, USA

[R] Non-Communicable Diseases Research Center, Tehran University of Medical Sciences, Tehran, Iran

[S] Department of Biomedical Sciences, Humanitas University, Milan, Italy

[T] Department of Epidemiology and Biostatistics, Western University, London, ON, Canada

[U] School of Public Health, Johns Hopkins University, Baltimore, MD, USA

[V] Family Medicine Department, Texas Tech University, El Paso, TX, USA

[W] Facultad de Salud (Faculty of Health), Universidad Santiago de Cali, Cali, Colombia

[X] Public Health Department, National University of Colombia, Bogota, Colombia

[Y] Epidemiology and Public Health Evaluation Group, National University of Colombia, Bogota, Colombia

[Z] Department of Epidemiology and Biostatistics, Semey Medical University (SMU), Semey, Kazakhstan

[AA] Department of Community Medicine, Datta Meghe Institute of Medical Sciences, Sawangi, India

[AB] Department of Health Behavior, Texas A&M University, College Station, TX, USA

[AC] Departamento de Responsabilidade Social (Department of Social Responsibility), Oswaldo Cruz German Hospital, São Paulo, Brazil

[AD] Brazilian Centre for Evidence-based Healthcare, Joanna Briggs Institute, São Paulo, Brazil

[AE] Department of Microbiology and Immunology, Northwestern University, Chicago, IL, USA

[AF] Department of Biological and Chemical Sciences, Michael and Cecilia Ibru University, Delta State, Nigeria

[AG] Department of Public Health and Community Medicine, Tanta University, Tanta City, Egypt

[AH] School of Public Health, Texila American University, Guyana, Guyana

[AI] Drexel Dornsife School of Public Health, Drexel University, Philadelphia, PA, USA

[AJ] Department of Medicine, University of Alberta, Edmonton, AB, Canada

[AK] Russell H. Morgan Department of Radiology and Radiological Science, Johns Hopkins University, Baltimore, MD, USA

[AL] Department of Radiology, University of Southern California, Los Angeles, CA, USA

[AM] Department of Orthopaedic Surgery, Massachusetts General Hospital, Boston, MA, USA

[AN] Department of Neurosurgery, Medical College of Wisconsin, Milwaukee, WI, USA

[AO] Sina Trauma and Surgery Research Center, Tehran University of Medical Sciences, Tehran, Iran

[AP] Department of Public Health Sciences, University of Chicago, Chicago, IL, USA

[AQ] Health Economics and Policy Management, Johns Hopkins University, baltimore, MD, USA

[AR] Department of Public Health, South Wales University, Treforest, UK

[AS] Osun State Hospital Management Board

[AT] T. H. Chan School of Public Health, Harvard University, Boston, MA, USA

[AU] School of Health Professions and Human Services, Hofstra University, Hempstead, NY, USA

[AV] Department of Anesthesiology, Montefiore Medical Center, Bronx, NY, USA

[AW] College of Health, Wellbeing and Life Sciences, Sheffield Hallam University, Sheffield, UK

[AX] College of Arts and Sciences, Ohio University, Zanesville, OH, USA

[AY] Department of Radiology, University of Washington, Seattle, WA, USA

[AZ] Cardiothoracic Imaging Section, University of Washington, Seattle, WA, USA

[BA] School of Health Sciences, Kristiania University College, Oslo, Norway

[BB] Department of International Health and Sustainable Development, Tulane University, New Orleans, LA, USA

[BC] Internal Medicine Department, MedStar Health, Washington, DC, USA

[BD] University Centre Varazdin, University North, Varazdin, Croatia

[BE] Department of Physical and Environmental Sciences, Texas A&M University, Corpus Christi, TX, USA

[BF] Shiraz University of Medical Sciences, Shiraz, Iran

[BG] Division of Endocrinology and Diabetes, University of Vermont, South Burlington, VT, USA

[BH] Department of Public Health, Johns Hopkins University, Baltimore, MD, USA

[BI] MyungSung Medical College, Addis Ababa, Ethiopia

[BJ] School of Public Health, Johns Hopkins University, Rockville, MD, USA

[BK] Department of Psychiatry and Behavioural Neurosciences, McMaster University, Hamilton, ON, Canada

[BL] Department of Psychiatry, University of Lagos, Lagos, Nigeria

[BM] Department of Neurosurgery, University of California San Francisco, San Francisco, CA, USA

[BN] Ottawa Hospital Research Institute, Ottawa, ON, Canada

[BO] Department of Cardiovascular Medicine, University of Tennessee, Nashville, TN, USA

[BP] Department of Genetics, Yale University, New Haven, CT, USA

[BQ] Department of Physiology and Biomedical Engineering, Mayo Clinic, Rochester, MN, USA

[BR] Department of Biostatistics, Epidemiology, and Informatics, University of Pennsylvania, Philadelphia, PA, USA

[BS] Pharmacy Practice and Science, University of Nebraska Medical Center, Omaha, NE, USA

[BT] Department of Pharmacology and Toxicology, University of Antioquia, Medellin, Colombia

[BU] Warwick Medical School, University of Warwick, Coventry, UK

[BV] Department of Public Health, New Mexico State University, Las Cruces, NM, USA

[BW] Department of Medicine, Georgetown University, Washington, DC, USA

[BX] National Heart, Lung, and Blood Institute, National Institutes of Health, Rockville, MD, USA

[BY] Department of Biology, Morgan State University, Baltimore, MD, USA

[BZ] Department of Environmental Health Sciences, Tulane University, New Orleans, LA, USA

[CA] School of Medicine, Baylor College of Medicine, Houston, TX, USA

[CB] Department of Medicine Service, US Department of Veterans Affairs (VA), Houston, TX, USA

[CC] Division of Preventive Medicine, University of Alberta, Edmonton, AB, Canada

[CD] School of Public Health, University of Alberta, Edmonton, AB, Canada

[CE] McGill University, Montreal, QC, Canada

[CF] Department of Environmental, Agricultural and Occupational Health, University of Nebraska Medical Center, Omaha, NE, USA

[CG] Department of Exact and Applied Social Sciences, Federal University of Health Science of Porto Alegre, Porto Alegre, Brazil
